# Potyviruses recruit host eIF4A3 to block m^6^A-mediated RNA decay by steric hindrance of viral RNA methylation in plants

**DOI:** 10.1093/nar/gkaf1432

**Published:** 2026-01-08

**Authors:** Dezhi Peng, Laihua Dong, Pei Wang, Lianyi Zang, Jinhao Xie, Hao Wang, Xiangdong Li, Zaifeng Fan, Tao Zhou, Kaitong Du

**Affiliations:** State Key Laboratory of Maize Bio-breeding and Department of Plant Pathology, China Agricultural University, Beijing 100193, China; State Key Laboratory of Maize Bio-breeding and Department of Plant Pathology, China Agricultural University, Beijing 100193, China; State Key Laboratory of Maize Bio-breeding and Department of Plant Pathology, China Agricultural University, Beijing 100193, China; State Key Laboratory of Maize Bio-breeding and Department of Plant Pathology, China Agricultural University, Beijing 100193, China; Department of Plant Pathology, College of Plant Protection, Shandong Agricultural University, Taian 271018, Shandong, China; State Key Laboratory of Maize Bio-breeding and Department of Plant Pathology, China Agricultural University, Beijing 100193, China; State Key Laboratory of Maize Bio-breeding and Department of Plant Pathology, China Agricultural University, Beijing 100193, China; Department of Plant Pathology, College of Plant Protection, Shandong Agricultural University, Taian 271018, Shandong, China; State Key Laboratory of Maize Bio-breeding and Department of Plant Pathology, China Agricultural University, Beijing 100193, China; State Key Laboratory of Maize Bio-breeding and Department of Plant Pathology, China Agricultural University, Beijing 100193, China; State Key Laboratory of Maize Bio-breeding and Department of Plant Pathology, China Agricultural University, Beijing 100193, China

## Abstract

*N* ^6^-methyladenosine (m^6^A), a critical epitranscriptomic modification, regulates RNA metabolism and antiviral defenses. However, how pathogens evade m^6^A-mediated RNA decay in plants remains poorly understood. Here, we uncover a dynamic m^6^A modification arms race during infection of sugarcane mosaic virus (SCMV), a prevalent potyvirus that infects maize and causes 20%–80% yield loss. We demonstrate that maize m^6^A methyltransferase (ZmMTA) specifically deposits m^6^A at A^6556^ of the SCMV genomic RNA, enabling recognition by the m^6^A reader EVOLUTIONARILY CONSERVED C-TERMINAL REGION 23 (ZmECT23). ZmECT23 directly recruits the ZmCCR4-NOT (carbon catabolite repressor 4–negative on TATA) complex to facilitate viral RNA decay. Strikingly, SCMV counters the defense via its nuclear inclusion protein a protease (NIa-Pro), which hijacks maize eukaryotic initiation factor 4A-III (ZmeIF4A3) into viral replication complexes. ZmeIF4A3 sterically blocks ZmMTA-mediated m^6^A deposition, thereby preventing viral RNA from degradation. Mechanistic conservation is observed in potato virus Y and turnip mosaic virus, two other potyviruses that are modified with m^6^A. Our study identifies eIF4A3 as a key m^6^A regulator in plants and reveals a strategy used by potyviruses to subvert m^6^A-based immunity via exploiting host RNA helicases. These findings provide mechanistic insights into host-pathogen interactions as mediated by m^6^A and suggest eIF4A3 as a potential target for engineering m^6^A-based antiviral crops.

## Graphical Abstract

**Graphical Abstract:**
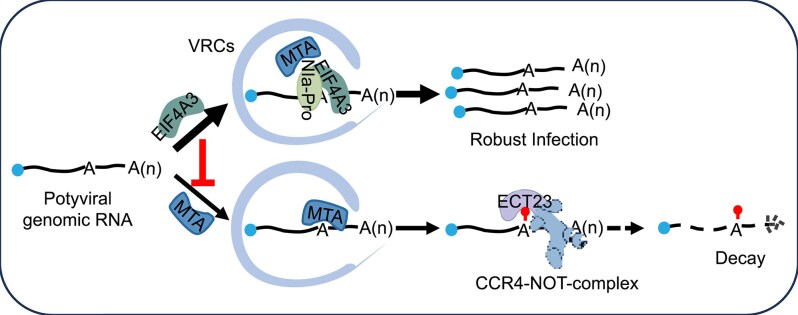

## Introduction

*N* ^6^-methyladenosine (m^6^A), a common modification of messenger RNA (mRNA) in eukaryotes, plays a crucial role in numerous physiological, and is explored by pathogens during host-pathogen interactions [1–3]. The m^6^A modification is meticulously targeted to the DRACH (D = A, G, or U; R = A or G; H = A, C or U) sequence motif, which is predominantly found in 3′ untranslated region (3′ UTR), within long exons, and in regions near stop codons [4–5]. In plants, the dynamics of m^6^A on mRNAs are tightly regulated by methyltransferases (known as “writers”) and demethylases (referred to as “erasers”). The outcomes of m^6^A modification are determined by m^6^A-binding proteins, also called “readers” [6–9]. The diversity of reader proteins underlies the multifaceted roles of m^6^A, including its prominent function in mediating RNA decay [10, 11]. However, molecular mechanisms by which m^6^A reader proteins govern RNA fate in plants remain largely unknown.

The eukaryotic initiation factor 4A3 (eIF4A3), also known as DEAD-box RNA helicase 2 (RH2), is a specialized and central component of the exon junction complex (EJC) and a key player in mRNA processing [12]. Together with MLN51 (metastatic lymph node 51) and Magoh/Y14 as a heterodimer, eIF4A3 orchestrates crucial mRNA functions, including RNA splicing, transport, translation, and decay [13–17]. In rice, two eIF4A3 homologs, OsRH2 and OsRH34 (RNA helicase 34), have been shown to regulate plant height as well as pollen and seed development [18]. Furthermore, levels of *Arabidopsis eIF4A3* (*AteIF4A3*) transcripts are markedly upregulated under abiotic stress, with *AteIF4A3*-deficient mutants exhibiting increased vulnerability to temperature fluctuations [19], highlighting its importance in stress response.

Most notably, recent studies highlight the critical role of eIF4A3 in precluding m^6^A deposition in unmethylated regions of the transcriptome in mammalian cells [20–22]. The silencing of *HseIF4A3* expression results in a substantial elevation in m^6^A levels across the transcriptome, which is attributed to its inhibitory effect on m^6^A methylation by sterically hindering METTL3 [21, 22]. However, the conservation and functional implications of this regulatory mechanism in plants are still unknown. Moreover, the role of eIF4A3 as an RNA m^6^A suppressor in the stress response of eukaryotes has yet to be explored.

Potyviruses, the largest and most economically devastating group of plant RNA viruses, belong to the family *Potyviridae* [23–25]. Among these viruses, sugarcane mosaic virus (SCMV) stands out as a particularly devastating member, infecting three major crops: maize (resulting in 20%–80% yield loss), sugarcane (causing 10%–50% yield loss), and sorghum [26]. Recognized as one of the top-10 most economically damaging plant viruses worldwide, SCMV has been reported in 84 countries across six continents [24, 27]. Successful viral infection in plants is contingent upon compatible interactions between the virus and its hosts [25]. Given their compact genomes and limited protein-coding capacity, viruses rely heavily on host factors to complete their infection cycle, leading to diseases in host crops. Despite their agricultural importance, recessive resistance genes and molecular mechanisms of potyvirus-host interactions remain largely unknown.

The presence of m^6^A in viral genomic RNAs was first documented over four decades ago [28]. Yet, the functions of m^6^A on viral genomic RNA remained largely unexplored. Recent technical advancements in the detection of m^6^A modifications have markedly enhanced our understanding of its roles in regulating viral infections [29–31]. For instance, YTHDF proteins, which serve as “readers” of m^6^A modifications, were re-localized to lipid droplets, the assembly sites of hepatitis C virus (HCV) viral particles, to recognize HCV genomic RNA with m^6^A, leading to a reduction in HCV particle production [32]. Conversely, the nuclear inclusion protein b of wheat yellow mosaic virus recruits the methyltransferase B (TaMTB), known as the “writer,” to enhance m^6^A modification on genomic RNA1, thereby stabilizing the viral RNA and promoting infection [33]. These findings underscore the critical role of m^6^A modification in host-virus interactions. Most recently, methylation RNA immunoprecipitation sequencing (MeRIP-seq) has revealed the presence of m^6^A modifications in potyviruses, including plum pox virus (PPV), potato virus Y (PVY), and turnip mosaic virus (TuMV), with notable enrichment in specific regions [34, 35]. However, the precise locations and functional importance of these m^6^A modifications in the regulation of potyviral infections remain to be elucidated.

In this study, we identified A^6556^ on SCMV genomic RNA as the site catalyzed for m^6^A modification by methyltransferase ZmMTA, using MeRIP-seq and single-base elongation and ligation-based quantitative polymerase chain reaction amplification method (SELECT-qPCR). This modification enables m^6^A reader ZmECT23 to recruit the ZmCCR4-NOT complex, promoting viral RNA degradation and reducing the infection rate. SCMV counteracts this defense via its NIa-Pro protein, which hijacks ZmeIF4A3 into viral replication complexes (VRCs). Using RNA-protein competition assays and electrophoretic mobility shift assays (EMSAs), we demonstrate that binding of eIF4A3 sterically blocks ZmMTA-mediated m^6^A deposition at A^6556^, thereby protecting viral RNA from degradation. Gene silencing of *ZmeIF4A3* in maize elevated m^6^A levels in viral genomic RNA and inhibited viral infection, while its overexpression promoted infection and exacerbated symptoms. Importantly, eIF4A3 played a similar role in PVY and TuMV infection: transgenic expression of *eIF4A3* in *Nicotiana benthamiana* significantly enhanced viral infection. This study reveals that eIF4A3 is a key suppressor of m^6^A methylation in plants and that potyviruses hijack eIF4A3 to evade epitranscriptomic immunity, pointing out the potential of targeting eIF4A3 for engineering crops with resistance to potyviruses.

## Materials and methods

### Plant growth conditions and virus inoculation

Maize seedlings of the inbred line B73, *ZmeIF4A3-*KD *Mutator* transposon insertion mutant, and *ZmeIF4A3*-EMS induced heterozygous mutant were germinated in darkness at 25°C for 3 days. After germination, seedlings were transplanted into pots and grown in a growth chamber under a 16-h light period at 25°C and an 8-h dark period at 22°C. *Nicotiana benthamiana* plants were grown under the same conditions as described earlier. For CMV-based gene silencing assays, maize seedlings were grown in growth chambers maintained at 20°C and 18°C (day/night) under the same photoperiod conditions. SCMV-GFP and its derivatives, including SCMV-G^6556^-GFP, SCMV-GFP/ΔGDD, SCMV-G^6556^-GFP/ΔGDD, SCMV-GUS-3Flag, and SCMV-ZmeIF4A3-3Flag, were propagated separately in maize seedlings. The upper leaves of infected maize seedlings, confirmed to contain the intended virus mutations or insertions, were used for subsequent virus inoculations. Virus inoculation was performed as previously described [36].

### Extraction of SCMV particles

SCMV particles were purified following the method described by Berger and Shiel [37]. Briefly, ~100 g of SCMV-infected maize leaves displaying clear symptoms were used for viral particles extraction. The leaves were cut into small pieces and ground into powder using liquid nitrogen or a juicer. The powdered material was then added to 400 ml of Extraction Buffer I [0.5 M potassium phosphate buffer (pH 7.2), 0.01 M copper reagent, 1% *β*-mercaptoethanol], and thoroughly mixed. The mixture was filtered through a single layer of nylon mesh, and 20% (v/v) CCl_4_ was added to the filtrate. After stirring for 20–30 min, the solution was centrifuged at 8500 rpm for 25 min. The supernatant was adjusted with 0.25 M NaCl and 6% (w/v) PEG 6000, stirred until completely dissolved, and incubated at 4°C for 2 h. The solution was centrifuged at 8500 rpm for 30 min, and the pellet was resuspended in Extraction Buffer II (0.05 M potassium phosphate buffer, pH 7.2; 1% Triton X-100) and incubated overnight at 4°C. Following incubation, the suspension was centrifuged at 7000 rpm for 10 min to remove any precipitate. The supernatant containing crudely purified virus particles was aliquoted into 100 µl portions and stored at −80°C for future use.

### Dot blot assays

The dot blot assays were performed as previously reported [11]. Total RNA was extracted using the TRIzol reagent (TransGen Biotech, Q30704). mRNA was purified from total RNA using the Poly(A) RNA Isolation Kit (Thermo Fisher Scientific, Cat. No. 61002) according to the manufacturer’s instructions, with two successive rounds of oligo(dT)-conjugated magnetic bead selection to maximize purity. Briefly, 50 μg of total RNA was adjusted to 100 μl with DEPC-treated water, mixed with an equal volume of Binding Buffer, and heated at 65°C for 2 min to disrupt secondary structures, followed by immediate cooling on ice. The RNA mixture was incubated with 0.5 mg of washed oligo(dT) magnetic beads (resuspended in 100 μl Binding Buffer) for 15 min at room temperature with gentle rotation. Beads were separated on a magnetic stand, washed twice with Washing Buffer B, and poly(A) RNA was eluted with 10–20 μl of cold 10 mM Tris–HCl by heating at 80°C for 2 min. The eluted mRNA was subjected to a second round of binding, washing, and elution to further remove residual ribosomal RNAs and transfer RNAs. Then, the purified mRNA was diluted to final concentrations of 500, 200, and 50 ng/μl in RNase-free water, while SCMV particles were diluted to 10, 5, and 2 µg/μl in sodium phosphate buffer. Both mRNA and SCMV samples were denatured at 95°C for 5 min, immediately flash-frozen on ice, and 3 µl of each sample was spotted onto a Hybond-N+ membrane. Samples were crosslinked using UV irradiation at 254 nm with an energy dose of 0.12 J/cm^2^ for 1 min. The membrane was washed with 10 ml of washing buffer [1 × phosphate buffered saline (PBS), 0.02% Tween-20] and blocked in 10 ml of blocking buffer (1 × PBS, 0.02% Tween-20, 5% non-fat milk) for 1 h at room temperature with gentle agitation. Subsequently, the membrane was incubated with anti-m^6^A antibody (1:200 diluted, Beyotime, AF7407) in antibody dilution buffer (1 × PBS, 0.02% Tween-20, 5% non-fat milk) at room temperature with gentle shaking for at least 2 h. After three washes with TBST, the membrane was incubated with an HRP-conjugated secondary antibody for 1 h before imaging. RNA was stained with 0.1% methylene blue (Bio Basic Inc, MB0342), and SCMV particles were immunoblotted using SCMV CP rabbit polyclonal antibody (1:5000, produced in our laboratory) to confirm equal loading.

### RNA extraction and quantitative real-time PCR analysis

Total RNA was extracted from plant leaves using the TRIzol reagent in accordance with the manufacturer’s instructions. Equal amounts of each RNA sample were then reverse transcribed into complementary DNA (cDNA) using the TRUE Reaction Mix (Aidlab, PC18). Quantitative real time-PCR analysis was conducted on an ABI QuantStudio 6 Flex Real Time PCR system (Applied Biosystems Inc.) using the ChamQ Blue Universal SYBR qPCR Master Mix (Vazyme, Q312-02). The maize *ubiquitin* gene (*ZmUbi*) was used as an internal control to normalize gene expression levels, and relative expression levels of the target genes were calculated using the 2^−∆∆CT^ method. Primers for qRT-PCR are provided in Supplementary Table S1.

### Methylation RNA immunoprecipitation sequencing

MeRIP-seq was performed according to previously described [38]. Purified mRNA (25 µg) was fragmented into 100–200 nucleotide fragments by incubating at 94°C for 5 min in RNA fragmentation buffer (10 mM Tris–HCl, pH 7.0, 10 mM ZnCl_2_), followed by termination of the reaction with 50 mM ethylenediaminetetraacetic acid (EDTA). The fragmented mRNA was incubated with 0.5 mg/ml of m^6^A antibody (202003, SYSY, Goettingen, Germany) in IP buffer [50 mM Tris–HCl, pH 7.4, 150 mM NaCl, 0.5% (vol/vol) Nonidet P-40] supplemented with RNase inhibitor for 2 h at 4°C. The RNA-antibody mixture was crosslinked with the UV light (254 nm, 0.12 J/cm^2^) for 2 min using a UV-light crosslinker. Protein G beads (Cell Signaling Technplogy, 37478S) were washed twice in 1 ml 1 × IP buffer, resuspended in 200 µl 1 × IP buffer, resuspended in 200 µl IP buffer, and added to the RNA-antibody solution, followed by incubation for 2 h at 4°C. Subsequently, the beads were collected and washed five times with 1 × IP buffer followed by vigorous shaking in elution competition buffer [50 mM Tris–HCl (pH 7.4), 150 mM NaCl, 0.5% (vol/vol) Nonidet P-40, 200 U RNasin, and 6.7 mM m^6^A] for 1 h to elute and enrich m^6^A-containing RNA fragments. The enriched m^6^A RNA was precipitated overnight at −80°C with 3 M sodium acetate (pH 5.2) and 3 × v/v 100% ethanol. Libraries were prepared using the TruSeq Stranded mRNA Library Prep Kit and sequenced on the Illumina NovaSeq™ 6000 platform (LC Bio Technology Co., Ltd., Hangzhou, China).

### MeRIP-qPCR

The MeRIP-qPCR experiments were conducted according to the principles of MeRIP-seq. Input and IP mRNAs were reverse transcribed using the TRUE Reaction Mix. Relative mRNA enrichment levels were measured using qRT-PCR and normalized to input levels. The primers used are listed in Supplementary Table S1.

### MeRIP of viral genomic RNAs

m^6^A RIP of viral genomic RNAs was performed according to a previously described method [33]. Total RNA (200 µg) extracted from SCMV-infected maize plants was incubated with m^6^A antibody and protein G beads at 4°C following the procedure for MeRIP-qPCR. A reaction lacking the specific m^6^A antibody served as a negative control. The eluted RNA was diluted into different concentration gradients, directly spotted onto a Hybond-N+ membrane, and hybridized with a specific Bio-SCMV probe (synthesized by Huada Biological Technology, Beijing, China; see Supplementary Table S1).

### Single-base elongation and ligation-based qPCR amplification method

The SELECT-qPCR is a single-base elongation and ligation-based qPCR amplification method that can distinguish single m^6^A site from A site [39]. A 17 µl pre-mixture solution was prepared containing 150 ng of mRNA, 5 µM dNTPs, 1 × CutSmart Buffer, 20 mM Tris–HAc (pH 7.9), 50 mM KAc, 10 mM MgAc_2_, 40 nM Up-Primer, 40 nM Down-Primer, and 100 µg/ml bovine serum albumin (BSA). The pre-mixture was incubated under the following temperature gradient: 90°C for 1 min, 80°C for 1 min, 70°C for 1 min, and 60°C for 1 min, followed by 50°C for 1 min, and 40°C for 6 min. Subsequently, 0.5 U of SplintR Ligase (NEB, M0375S), 0.01 U of *Bst* 2.0 DNA Polymerase (NEB, M0537S), and 10 nmol ATP are added to the mixture with a final volume of 20 µl. The reaction was incubated at 40°C for 20 min and denatured at 80°C for 20 min. Finally, qRT-PCR analysis was performed using the SELECT universal primers listed in Supplementary Table S1.

### Direct RNA sequencing

Direct RNA sequencing (DRS) libraries were prepared from poly(A)+ RNA enriched from SCMV-infected maize samples using the SQK-RNA004 kit (Oxford Nanopore Technologies, ONT). For adapter ligation, 9 μl poly(A)+ RNA, 3 μl NEBNext Quick Ligation Reaction Buffer (NEB, B6058), 1 μl RT Adapter (ONT, SQK-RNA004), and 2 μl T4 DNA ligase (NEB, M0202) were mixed and incubated at 25°C for 10 min. Reverse-transcription reagents were then added to the 15 μl ligation mixture: 8 μl 5 × First-Strand Buffer (NEB, M0681), 2 μl 10 mM dNTPs (NEB, N0447), 9 μl nuclease-free water, 4 μl 0.1 M dithiothreitol (DTT), and 2 μl Induro Reverse Transcriptase (NEB, M0681). The reaction was incubated at 50°C for 50 min, followed by 70°C for 10 min. The reverse-transcribed RNA was purified with 1.8 × Agencourt RNAClean XP beads (Beckman Coulter, A63987) and eluted in 23 μl nuclease-free water. Sequencing adapters were ligated by adding 8 μl NEBNext Quick Ligation Reaction Buffer, 6 μl RNA Adapter (ONT, SQK-RNA004), and 3 μl T4 DNA ligase; the product was bead-purified and eluted as above. Finally, 50 μl RNA Running Buffer (ONT, SQK-RNA004) was mixed with 35 μl nuclease-free water, and the library was loaded onto a PromethION flow cell (ONT) and sequenced for 48–72 h on a PromethION instrument. Library preparation and data analysis were performed by Seqhealth Technology Co., Ltd., Wuhan, China.

### *Agrobacterium*-mediated transient expression and immunoblotting analysis

The *Agrobacterium*-mediated transient expression assays were conducted according to the previously described method [40]. Briefly, *Agrobacterium tumefaciens* cultures containing specific construct combinations were infiltrated into the leaves of 4-week-old *N. benthamiana* plants. The leaves were harvested at 3 days post-infiltration, and total protein was extracted using a buffer containing 220 mM Tris–HCl (pH 7.4), 250 mM sucrose, 1 mM MgCl_2_, 50 mM KCl, and 10 mM *β*-mercaptoethanol. The extracted proteins were mixed with sodium dodecyl sulfate (SDS) sample buffer (100 mM Tris–HCl, pH 6.8, 10% SDS), boiled, and separated by 10% SDS–polyacrylamide gel electrophoresis (PAGE). Detection signals were developed with an ECL reagent as instructed (YEASEN, Shanghai, China; 36208ES76) and visualized using an Azure c400 Imager (Azure Biosystems), followed by quantification with ImageJ software. Antibodies used included GFP monoclonal antibody (TransGen Biotech, Beijing, China; HT801, 1:5000 diluted), anti-Flag mouse monoclonal antibody (Sigma–Aldrich, A8592, 1:8000 diluted), anti-c-Myc rabbit monoclonal antibody (Sigma–Aldrich, A5598, 1:8000 diluted), anti-GST mouse monoclonal antibody (CWBIO, CW0084M, 1:5000 diluted), anti-β-actin mouse monoclonal antibody (EASYBIO, Beijing, China; BE0028), and anti-SCMV CP antibody (1:5000 diluted), as previously described [41].

### Cucumber mosaic virus-based gene silencing in maize

The cucumber mosaic virus-based gene silencing (CMV-VIGS) experiments were conducted according to the previous description [42]. *Agrobacterium* cultures carrying pCMV101, pCMV301, or one of the four new plasmids (pCMV201-2b_N81_:*GUS*, pCMV201-2b_N81_:*ZmeIF4A3/ZmeIF4A3-like*, pCMV201-2b_N81_:*ZmeIF4A3-like*, pCMV201-2b_N81_:*ZmMTA*, pCMV201-2b_N81_:*ZmECT23*) were mixed in a 1:1:1 ratio and infiltrated into *N. benthamiana* leaves. Five days later, crude leaf extracts were prepared from the infiltrated leaves and used to inoculate maize seeds individually via the vascular puncture method. Eight days post CMV inoculation, challenge inoculation with SCMV-GFP-infected maize crude extracts was performed on the first true leaf.

### Electrophoretic mobility shift assay

The EMSA assays were performed using the Chemiluminescent EMSA Kit (Beyotime Biotechnology, D3308) according to the manufacturer’s instructions. Briefly, biotin-labeled RNA probes were synthesized (Huada Biological Technology, Beijing, China), and unlabeled RNA probes with the same sequence were used as cold probes for competitive analysis. His-tagged ZmeIF4A3, His-TF-tagged ZmMTA protein were purified from *Escherichia coli* BL21. A total of 2 µg of ZmeIF4A3 or ZmMTA protein was incubated with 0.01 µM biotin-labeled probe. Increasing amounts of unlabeled cold probes were added to the reaction mixture, bringing the final volume to 20 µl. The samples were then subjected to 8% native polyacrylamide gel electrophoresis, transferred to a Hybond-N+ membrane, and analysis by immunoblot using anti-Streptavidin-HRP from the EMSA Kit.

### RNA immunoprecipitation-qPCR assays

The RNA immunoprecipitation (RIP)-qPCR assays were performed as previously reported [43]. Plant samples were collected and ground in liquid nitrogen. PEB buffer [20 mM Tris–HCl, pH 7.5, 100 mM KCl, 5 mM MgCl_2_, 0.5% (vol/vol) Nonidet P-40, 1 × cocktail protease inhibitor (Abmole, M5293), and 200 U/ml RNase inhibitor (Accurate Biology, AG11608)] was then added to the lysate in a 1:1 ratio, mixed thoroughly by vortexing, and incubated on ice for 30 min. The mixture was centrifuged at 10 000 × *g* for 15 min at 4°C, and this process was repeated five times before the supernatant was collected. Additionally, 200 µl of the supernatant was taken as the input control, while the remaining supernatant was subjected to IP with 30 µl of anti-Flag beads (Sigma–Aldrich, A4596) or 30 µl of protein G beads at 4°C for 4 h. For ZmMTA-specific RIP-qPCR, the supernatant was incubated with anti-ZmMTA antibody overnight at 4°C with rotation, followed by isolation of immune complexes using protein A beads. After that, the beads were washed six times with NT2 buffer (50 mM Tris–HCl, pH 7.5, 150 mM NaCl, 1 mM MgCl_2_, 0.05% (vol/vol) Nonidet P-40), and the supernatant was removed. Proteinase K (Beyotime Biotechnology, ST533) was used to digest RNA–protein complexes at 55°C for 25 min. The IP and input RNAs were then reverse transcribed for qRT-PCR detection and normalized to input levels. RNA immunoprecipitated with IgG or IgA serves as the negative control. The primers used are listed in Supplementary Table S1.

### RNA stability assays

RNA stability assays in *N. benthamiana* were primarily conducted as previously reported [44, 45]. Briefly, recombinant expression plasmids were infiltrated into the leaves by *Agrobacterium*. After 72 h of incubation, the infiltration parts in the leaves were injected with 20 μg/ml actinomycin D (Sigma, A4262) dissolved in ddH_2_O. After culture for 30 min, leaf discs were taken and considered as time 0 controls, and subsequent samples were harvested every 3 h in triplicate. The mRNA levels were determined using qRT-PCR assays with specific primers (Supplementary Table S1). *NbActin* served as the internal control for data normalization. Degradation curves were modeled using the equation Y = exp(−A × X), where X represents time and A denotes the decay constant. All data were normalized to the 0 h value (set to 1). Data fitting and statistical analyses were performed with GraphPad Prism 8.0 software.

## Surface sensing of translation assay

Surface sensing of translation (SUnSET) assay was modified from a published protocol [46]. Fully expanded leaves from 3-week-old B73 or *ZmeIF4A3*-KD maize seedlings were excised; proximal and distal ends were removed to obtain central segments of 5–6 cm. For each sample, 6–8 segments were placed in 5 ml centrifuge tubes containing distilled water and supplemented with 10 ng/ml puromycin (Beyotime, ST551). The tubes were left open and placed in a vacuum desiccator connected to a vacuum pump. When the pressure reached −0.09 MPa, the vacuum was maintained for 40 min and then released. Leaf tissue was frozen and ground in liquid nitrogen, to which 2 × SDS loading buffer was subsequently added. After vigorous mixing, the lysates were boiled at 95°C for 10 min. Puromycin-labeled proteins were detected by immunoblotting with an anti-puromycin antibody (Sigma–Aldrich, MABE343; 1:5000 dilution).

### Polysome profiling

Polysome profiling was carried out as previously described with minor modifications [47]. Three-week-old B73 or *ZmeIF4A3*-KD maize seedlings (∼5 g per sample) were ground to a fine powder in liquid nitrogen and homogenized in 10 ml ice-cold polysome extraction buffer (200 mM Tris–HCl, pH 9.0, 200 mM KCl, 35 mM MgCl_2_, 25 mM ethylene glycol tetraacetic acid (EGTA), 1% Triton X-100, 1% Brij-35, 1% Nonidet P-40, 1% Tween-20, 5 mM DTT, 100 μg/ml cycloheximide, 100 μg/ml chloramphenicol, and 1 mM phenylmethylsulfonyl fluoride (PMSF)). The homogenate was incubated on ice for 30 min and then filtered sequentially through two layers of gauze and two layers of Miracloth. The filtrate was centrifuged at 8000 rpm for 20 min at 4°C, passed through a 70 μm cell strainer, and centrifuged again at 8000 rpm for 20 min at 4°C. The supernatants were loaded on the top of a 1.7 M of sucrose cushion and centrifuged at 50000 rpm (SW 70 Ti Swinging Bucket rotor; Beckman Coulter) for 3 h at 4°C. The ribosome pellet was resuspended in 600 ml of resuspension buffer (200 mM Tris–HCl, pH 9.0, 200 mM KCl, 35 mM MgCl_2_, 25 mM EGTA, 5 mM DTT, 100 μg/ml chloramphenicol, and 100 μg/ml CHX). Then the solution was layered over a 20%–60% sucrose density gradient and centrifuged at 50000 rpm (SW 55 Ti Swinging Bucket rotor; Beckman Coulter) for 90 min at 4°C. Gradients were fractionated into 15 equal aliquots while continuously monitoring absorbance at 260 nm using a Gradient Fractionator (Biocomp) equipped with an in-line UV detector.

### Yeast two-hybrid assays

The yeast two-hybrid (Y2H) assays were performed according to the manufacturer’s instructions (Clontech, San Francisco, CA). In brief, full-length coding regions of *ZmeIF4A3, ZmeIF4A3-like, ZmUPF2, ZmUPF3, ZmCCR4, ZmECT23*, SCMV *NIa-Pro*, and its derivatives were individually cloned into the pGADT7 or pGBKT7 vector, followed by co-transformation, in different combinations, into the yeast Gold strain. The transformants were cultured on selective media lacking tryptophan and leucine (SD/-Trp-Leu) to confirm the transformation. Subsequently, yeast cells were grown on high-stringency selective media lacking tryptophan, leucine, histidine, and adenine (SD/-Trp-Leu-His-Ade) at 30°C for 3–5 days to screen for interactions between co-transformed proteins.

### Luciferase complementation imaging assay

The luciferase complementation imaging (LCI) assay was performed as previously described [48]. Pairs of corresponding plasmids were agroinfiltrated into *N. benthamiana* leaves. After 3 days of infiltration, leaf samples were collected and sprayed with 1 mM luciferin (Vazyme, DD1210). Imaging was conducted using a cold charge-coupled device camera (iXon, Andor Technology, Belfast, UK), and photographs were taken after a 20 min exposure.

### Subcellular localization assays

The subcellular localization assays were conducted as described previously [49]. *Agrobacterium* strain containing expression plasmids was mixed and infiltrated into *N. benthamiana* leaves. After 72 h of infiltration, leaf samples (1–2 cm^2^) were collected, sliced, and examined for epidermal cell fluorescence using confocal microscopy (Zeiss LSM 800) (GFP was excited at 488 nm and captured at 510–550 nm; RFP was excited at 543 nm and captured at 590–630 nm).

### Co-immunoprecipitation and pull-down assays

Co-immunoprecipitation (Co-IP) assays were conducted with minor modifications as previously described [50]. GUS-3Flag, ZmeIF4A3-like-3Flag, ZmeIF4A3-3Flag, and its derivatives were co-expression with EGFP-NIa-Pro in *N. benthamiana* leaves via *Agrobacterium*-mediated infiltration. Three days post infiltration, the leaves were harvested, frozen in liquid nitrogen, and ground into a powder. Total proteins were extracted from 3 g of the powdered tissue using 3 ml of IP buffer (150 mM NaCl, 50 mM Tris–HCl, pH 7.5, 1 mM EDTA, 10% glycerol, 0.1% TritonX-100, 1 mM protease inhibitor cocktail). The extract was centrifuged at 12000 rpm and 4°C for 10 min, repeated four times, and the supernatant was collected. Thirty microliters of anti-Flag beads were added to the supernatant, and the mixture was incubated at 4°C with rotation for 3–4 h. After six washes with IP buffer at 4°C, immunoblot analysis was performed using anti-Flag and anti-GFP antibodies.

The pull-down assays were carried out as described previously [51]. Briefly, the pET-30a (+) vector was used to express His-GFP-ZmECT23 and His-ZmeIF4A3-like-3Flag, the pGEX-4T-3 vector was employed to express GST-NIa-Pro and GST, and the pCold-His-TF vector was used to express His-TF-GFP, His-TF-ZmeIF4A3-3Myc, His-ZmeIF4A3-like-3Flag, and His-TF-3Flag-ZmCCR4 fusion proteins. Approximately 5 µg of purified GFP-ZmECT23 and GFP fusion proteins were incubated with 3Flag-ZmCCR4, while GST-NIa-Pro and GST were incubated with His-TF-ZmeIF4A3-3Myc or His-ZmeIF4A3-like-3Flag. All reactions were conducted in 500 µl of incubation buffer (50 mM Tris–HCl, pH 6.8, 300 mM NaCl, 1.5% glycerol, 0.6% Triton X-100, and 0.1% Tween) at 4°C for 3 h. The beads were washed six times with the incubation buffer. Subsequently, the washed beads were boiled in 2 × SDS loading buffer, and the proteins were separated by SDS–PAGE for subsequent immunoblot analysis.

### RNA pull-down assay

The RNA pull-down assay was conducted as previously described [52]. Briefly, biotin-labeled SCMV RNA fragments were synthesized by Huada Biological Technology (Beijing, China). Subsequently, 500 pmol of 5ʹ-biotinylated RNA was incubated with purified protein samples. Streptavidin magnetic beads (Thermo Fisher Scientific, 21344) were then added to the RNA–protein complexes and incubated at 4°C for 4 h. The complexes were washed five times with a buffer containing 10 mM Tris–HCl (pH 7.5), 10 mM MgCl_2_, 150 mM NaCl, 0.05% Tween-20, 1 mM protease inhibitor cocktail, and 100 U/ml RNase inhibitor, followed by immunoblot analysis.

### Tobacco rattle virus-based gene silencing

Tobacco rattle virus-based gene silencing (TRV-VIGS) was performed as described by Liu *et al.* [53]. A 300-bp *NbeIF4A3* fragment was inserted into pTRV2 (pTRV2:*NbeIF4A3*). *Agrobacteria* carrying pTRV1 and either pTRV2:*mCherry* or pTRV2:*NbeIF4A3* were mixed 1:1 and infiltrated into 4-week-old *N. benthamiana* leaves. Seven or eight days later, TuMV or PVY sap was inoculated onto the upper leaves. At 12 days post–inoculation (dpi), qRT–PCR was performed to evaluate the silencing efficacy of *NbeIF4A3* and to quantify viral accumulation.

## Results

### m^6^A modification at A^6556^ in SCMV genomic RNA attenuates viral replication

The precise locations and possible functions of m^6^A modifications on potyviral genomic RNA are yet to be fully elucidated [34, 35]. In this study, we utilized SCMV as a model to identify the precise locations of m^6^A modifications on its genomic RNA, aiming to shed light on their potential regulatory roles in the viral life cycle.

**Figure 1.**
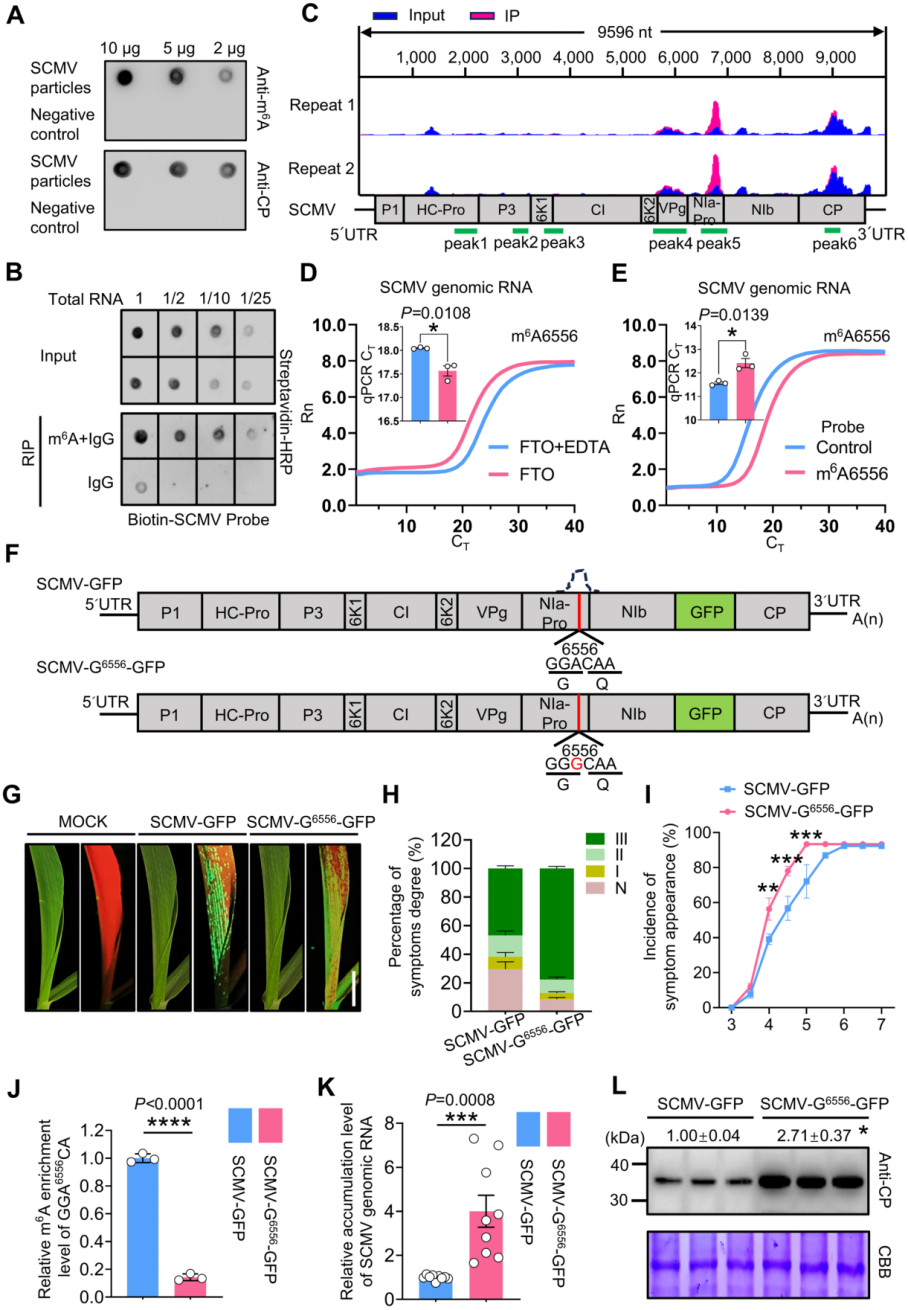
The m^6^A modification at A^6556^ of the SCMV genomic RNA reduces viral pathogenesis. (**A**) Dot blot analysis of SCMV particles showed the presence of m^6^A modification on SCMV genomic RNA. Unmodified *in vitro*-transcribed RNAs (IVT RNAs) were used as the negative control. (**B**) RIP followed by dot blotting showed that SCMV genomic RNA undergoes m^6^A modification during viral infection. RIP assays were performed using an m^6^A-specific antibody to immunoprecipitate m^6^A-modified RNAs from SCMV-infected maize leaves. The immunoprecipitated RNAs were subsequently spotted onto a Hybond-N+ membrane and then detected using a biotin-labeled SCMV RNA-specific probe. (**C**) Methylated RNA immunoprecipitation with next-generation sequencing (MeRIP-seq) (representative of two independent biological replicates) of SCMV genomic RNA illustrated the distribution of m^6^A peaks (red bars) mapped to the SCMV genomic RNA. The signal from input samples was represented by blue bars. A schematic diagram of the SCMV genomic RNA was shown below, with green lines indicating the location of the m^6^A-enriched sequences. The strongest enrichment was observed within the *NIa-Pro* region. (**D, E**) Fluorescence amplification curves and bar plot of the threshold cycle (CT) from qPCR indicated that the presence of m^6^A modification at A^6556^ in the SCMV genomic RNA. The m^6^A levels at position 6556 were assessed following treatment with the fat mass and obesity-associated protein (FTO), as shown in panel (D). In panel (E), the control probe targets a non-m^6^A site; the m^6^A6556 probe targets A^6556^. The significantly higher CT value detected by the m^6^A6556 probe demonstrated the presence of m^6^A modification at A^6556^. Rn represents the raw fluorescence of the associated well normalized to the fluorescence of the passive reference dye (ROX). Values are means ± SE (two-tailed Student’s *t* test, *n* = 3 independent experiments, *P < .05). (**F**) Schematic diagram of the SCMV-G^6556^-GFP mutation. The mutated nucleotide is marked in red. The black peak corresponds to the m^6^A peak. (**G**) SCMV-G^6556^-GFP caused stronger GFP fluorescence compared with SCMV-GFP. Photographs were taken at 5 days post-infection (dpi). Scale bar, 5 cm. (**H**) Percentages of maize seedlings infected with SCMV-GFP or SCMV-G^6556^-GFP were assessed based on different disease symptom grades. Seedlings infected with SCMV-G^6556^-GFP showed more severe mosaic symptoms, with an increased proportion of severe disease symptoms (grade III) and a decreased proportion of asymptomatic cases (grade N), relative to SCMV-GFP-infected seedlings. (**I**) Maize seedlings infected with SCMV-G^6556^-GFP displayed mosaic symptoms earlier, from 4 to 5 dpi, compared to those infected with SCMV-GFP. Data are presented as means ± SE (*n* = 3 independent experiments). Statistical significance at same time point was determined by a two-tailed Student’s *t*-test (**P < .01; ***P < .001). (**J**) MeRIP-qPCR assays indicated reduced m^6^A levels at peak5 in SCMV-G^6556^-GFP-infected maize seedlings compared to SCMV-GFP-infected seedlings. Values are means ± SE (two-tailed Student’s *t* test, *n* = 3 independent experiments, **P < .01). (**K, L**) SCMV-G^6556^-GFP exhibited higher levels of SCMV genomic RNA and coat protein (CP) compared to the SCMV-GFP. Quantitative reverse transcription polymerase chain reaction (qRT-PCR) was used to assess SCMV genomic RNA levels, with *ZmUbi* transcripts serving as internal controls. Immunoblotting assay showed viral CP levels at 5 dpi. Band intensity was quantified using ImageJ software, and values below the top panel represent the relative quantification of band intensity. The CP band intensity of SCMV-GFP was normalized to 1.00. In panel (K) and (L), data are presented as means ± SE (Student’s *t* test, *n* = 9 plants from three independent experiments, *P < .05; **P < .01).

We purified viral particles from SCMV-infected maize tissues and performed dot blot assays to detect m^6^A modifications. The purified viral particles exhibited a positive reaction with anti-m^6^A polyclonal antibodies, whereas the negative control of *in vitro*-transcribed RNA did not, indicating the presence of m^6^A modifications in SCMV genomic RNA (Fig. 1A). Furthermore, SCMV genomic RNA extracted from particles and subjected to dot blot assays verified this modification (Supplementary Fig. S1A). We further employed RIP assays by anti-m^6^A polyclonal antibodies to enrich for m^6^A-modified RNAs from SCMV-infected maize plants. Dot blot hybridization of the RIP products with a biotinylated SCMV-specific probe (Bio-SCMV_6543–6565 nt_) confirmed the enrichment of SCMV genomic RNA (Fig. 1B).

To precisely identify the specific m^6^A modification sites, we conducted MeRIP-seq on SCMV genomic RNA extracted from purified particles. The clean reads obtained from m^6^A-seq were aligned to the SCMV genomic RNA sequence, revealing six m^6^A peaks located within coding region (Fig. 1C and Supplementary Fig. S1B and C). Utilizing the sequence-based RNA adenosine methylation site predictor (SRAMP) tool, we predicted eleven potential m^6^A sites across these six peaks (Supplementary Table S2), which were subsequently validated using SELECT-qPCR (Supplementary Fig. S2). Additionally, MeRIP-qPCR assays and fluorescence measurements of fat mass and obesity-associated protein (FTO, to remove m^6^A modifications) treated samples jointly confirmed the m^6^A modification at A^6556^ within SCMV’s peak5 RNA sequence (Fig. 1D and E; Supplementary Fig. S1D). To estimate the m^6^A modification proportion at A^6556^, we performed Nanopore DRS on total RNA extracted from SCMV-infected B73 maize leaves. Specifically, the estimated methylation fraction was ~1.1% (112 modified reads out of 9972 viral reads, probability threshold >0.5) (Supplementary Table S3). These results collectively demonstrated that the A^6556^ in SCMV genomic RNA is subject to m^6^A modification.

To assess the role of m^6^A at A^6556^ during SCMV infection, we introduced a specific synonymous mutation (mutated A^6556^ to G, without changing the amino acid sequence) into the SCMV-GFP infectious clone, designated SCMV-G^6556^-GFP (Fig. 1F and Supplementary Fig. S1E). Maize plants infected with SCMV-G^6556^-GFP exhibited more severe mosaic symptoms compared to those infected with SCMV-GFP, with a higher proportion of severe symptoms (grade III) and a lower proportion of asymptomatic cases (grade N) (Fig. 1G and H) [49]. The infection rates of SCMV-G^6556^-GFP were higher than those of SCMV-GFP at the indicated time points (Fig. 1I). MeRIP-qPCR assays revealed a significant reduction in m^6^A modification levels in SCMV-G^6556^-GFP genomic RNA compared to that of SCMV-GFP (Fig. 1J). Furthermore, the accumulated levels of SCMV-G^6556^-GFP and CP were significantly higher than those of SCMV-GFP, with respective increases to ~4.0-fold and 2.7-fold (Fig. 1K and L). However, this enhancement was abolished when the A^6556^–G mutation was introduced into the replication-defective SCMV-GFP/ΔGDD backbone, as SCMV-G^6556^-GFP/ΔGDD and SCMV-GFP/ΔGDD mutants accumulated to comparable levels of viral RNA and CP (Supplementary Fig. S3) [54].

### ZmMTA directs m^6^A deposition at A^6556^ to restrict SCMV infection

**Figure 2.**
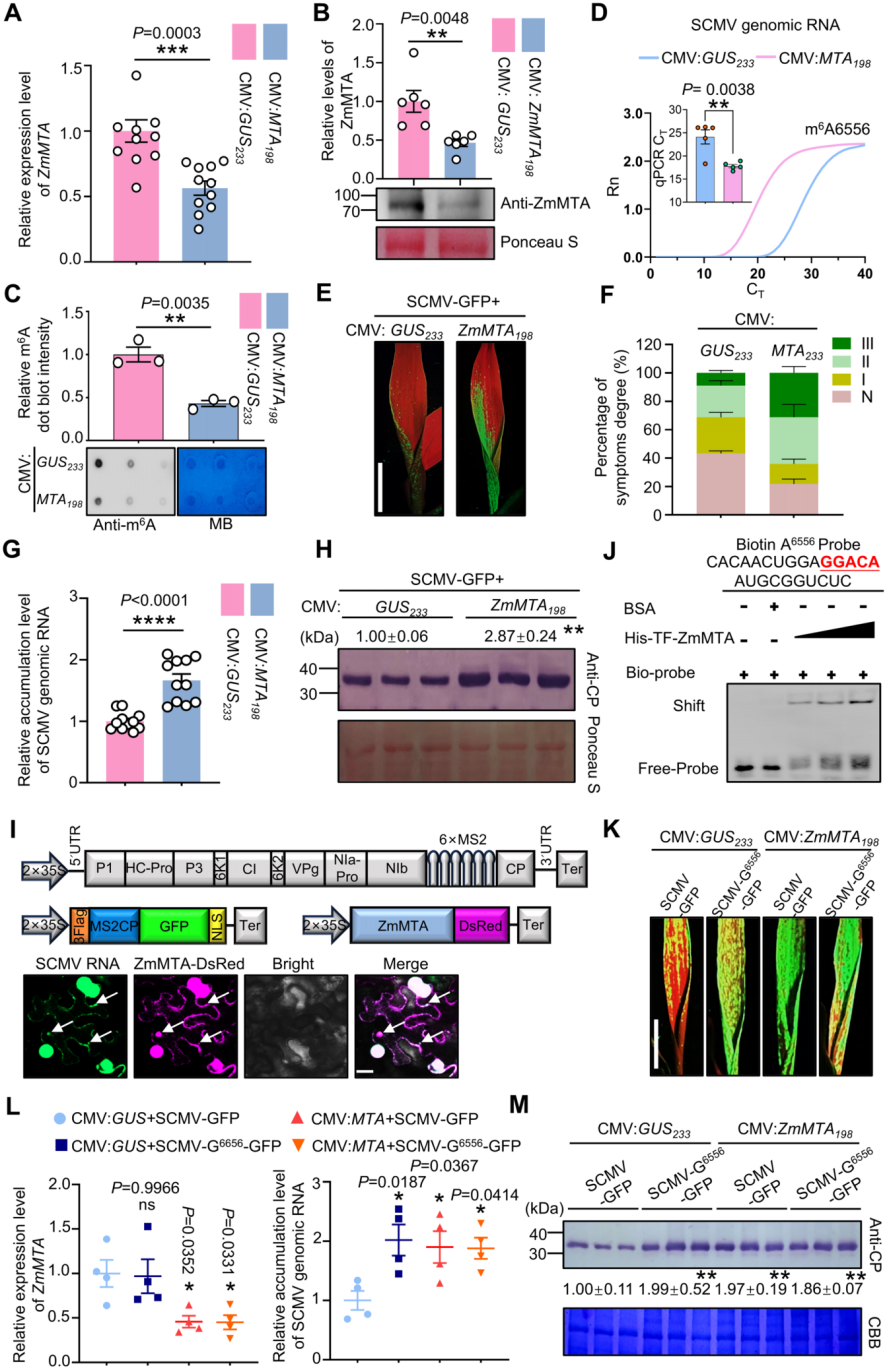
ZmMTA inhibits SCMV infection by catalyzing m^6^A modifications at A^6556^ site on SCMV genomic RNA. (**A**) *ZmMTA* expression levels were significantly reduced in the 1 SL of CMV:*ZmMTA*-infected plants compared to CMV:*GUS*-infected (control) plants using qRT-PCR analysis at 18 days post vascular puncture inoculation (VPI). (**B**) Immunoblotting analysis revealed the decreased the ZmMTA protein levels using anti-ZmMTA antibody. (**C**) Dot blot analysis indicated a significant reduction in m^6^A modification levels of total RNA in CMV:*ZmMTA*-infected plants, compared to control plants. MB staining used as a loading control. ImageJ software was used to quantify the m^6^A dot blot intensity. Data are presented as means ± SE (*n* = 3 independent experiments). (**D**) Knockdown of *ZmMTA* expression in maize plants inhibited m^6^A level at position A^6556^ of SCMV genomic RNA compare to control plants. Data are shown as means ± SE (two-tailed Student’s *t* test, *n* = 5 plants, **P < .01). (**E**) Stronger GFP fluorescence was observed in the 1 SL of SCMV-GFP-infected plants compared to control plants. Scale bar, 2 cm. (**F**) Plants infected with CMV:*ZmMTA* showed more severe mosaic symptoms relative to CMV:*GUS*-infected plants at 7 dpi. (**G**) SCMV genomic RNA accumulation levels in the 1 SL of *ZmMTA*-silenced or control plants were determined through qRT-PCR at 7 dpi. Data are expressed as the means ± SE (*n* = 12 plants from three independent experiments). (**H**) Immunoblotting analysis showed SCMV CP accumulation in *ZmMTA*-silenced and control plant leaves (*n* = 3 plants). (**I**) Co-localization of ZmMTA with SCMV RNA in cytoplasmic aggregates revealed by MS2-tethering assay. ZmMTA-DsRed (red) and MS2-tagged SCMV genomic RNA, labeled by MS2-CP-GFP (green), show clear co-localization in cytoplasmic aggregates (indicated by white arrows). Scale bar, 20 μm. (**J**) His-TF-ZmMTA directly bound to the biotinylated A^6556^ probe through EMSA. A biotin-labeled 25 bp fragment of the peak5 containing GGA^6556^CA is highlighted in bold, red, and underlined. BSA served as a negative control. Bound and shifted probes were detected with Streptavidin-HRP (upper). (**K**) Upper leaves of plants infected with SCMV-G^6556^-GFP displayed similar GFP fluorescence in *ZmMTA-*silenced plants compared to control plants. Scale bar, 2 cm. (**L, M**) No significant differences were observed in the accumulation levels of SCMV-G^6556^-GFP genomic RNA and CP between CMV:*ZmMTA* and CMV:*GUS*-infected maize plants (*n* = 4 plants). Statistical significance in panels (A–D) and (G, H) was assessed with two-tailed Student’s *t*-tests; panels (L, M) were analyzed by one-way analysis of variance (*P < .05; **P < .01; ***P < .001; ****P < .0001; ns, no significant difference).

To investigate the writer-mediated m^6^A deposition at position A^6556^ in the SCMV genomic RNA, we focused on ZmMTA, a core m^6^A methyltransferase in maize [55, 56]. We used CMV-VIGS to silence *ZmMTA* and evaluated its role in site-specific methylation at A^6556^. *ZmMTA*-silenced maize plants (CMV:*ZmMTA*) exhibited significantly reduced the accumulated levels of *ZmMTA* and m^6^A in total RNA compared to those in CMV:*GUS* controls (Fig. 2A–C). Following SCMV infection, m^6^A modification level at the A^6556^ site was significantly reduced in *ZmMTA*-silenced plants (Fig. 2D). Concurrently, these plants exhibited stronger GFP fluorescence and more severe mosaic symptoms in the upper leaves compared to CMV:*GUS* control plants (Fig. 2E and F). Consistent with these observations, accumulated levels of SCMV genomic RNA and CP were significantly increased to ~1.7-fold and 2.9-fold, respectively, in *ZmMTA*-silenced plants relative to controls (Fig. 2G and H).

Moreover, we showed that ZmMTA-DsRed co-localized with SCMV genomic RNA in cytoplasmic aggregates using MS2-tethering assays (Fig. 2I), and recombinant ZmMTA protein directly binds to GGA^6556^CA motif located within peak5 of SCMV genomic RNA using EMSA (Fig. 2J). These data suggested that ZmMTA could add a methyl at A^6556^. To further validate the specificity of A^6556^ methylation, we inoculated *ZmMTA*-silenced plants with SCMV-G^6556^-GFP. Notably, the accumulated levels of GFP fluorescence, SCMV genomic RNA, and CP were similar to those in control plants inoculated with SCMV-GFP or SCMV-G^6556^-GFP (Fig. 2K–M). Collectively, these results indicate that ZmMTA contributes to the installation of m^6^A at A^6556^ to restrict SCMV propagation.

### ZmECT23 destabilizes m^6^A-containing viral RNA through direct recruitment of the CCR4-NOT complex

**Figure 3.**
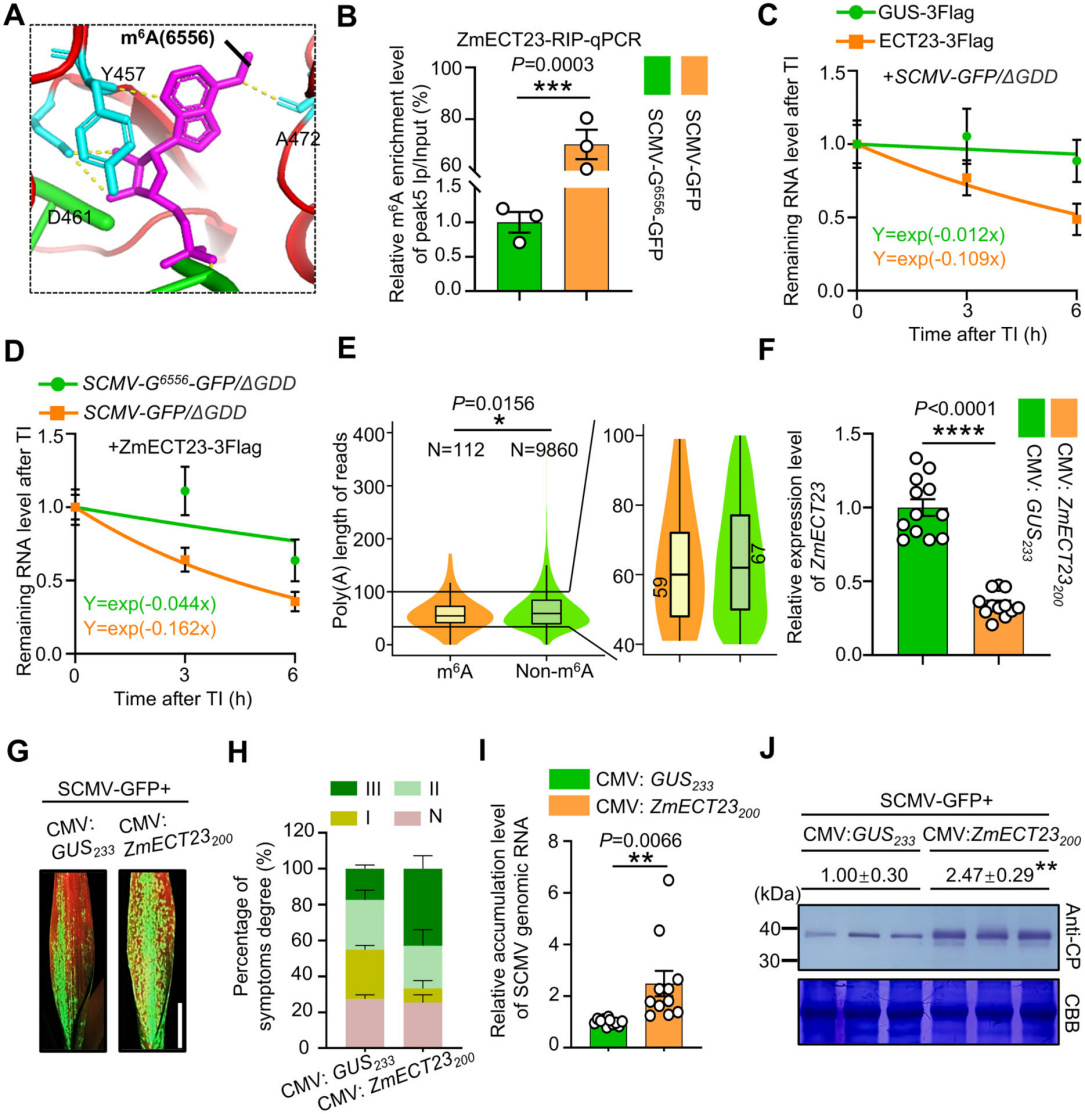
ZmECT23 recognizes the m^6^A modification at position A^6556^ of SCMV genomic RNA, leading to the destabilization of viral RNA. (**A**) AlphaFold3 predicted that ZmECT23 binds to the A^6556^ site of the SCMV genomic RNA. Within the peak5 region, the central adenine in the GGm^6^ACA sequence was methylated. The amino acid residues Tyr-457, Asp-461, and Ala-472 of ZmECT23 may directly interact with m^6^A through hydrogen bonds. The peak5 RNA sequence was displayed in magenta, with the m^6^A modification at position A^6556^ highlighted in bold black, and hydrogen bonds represented by yellow dashed lines. (**B**) RIP-qPCR analysis indicated that ZmECT23 binds to the peak5 region of SCMV genomic RNA containing the A^6556^ site in *N. benthamiana* cells, but not to the corresponding region in the SCMV-G^6556^-GFP mutant. Protein-RNA complexes were isolated from *N. benthamiana* leaves (*n* = 3 independent experiments) co-infiltrated with SCMV-GFP and ZmECT23-3Flag, or with SCMV-G^6556^-GFP and ZmECT23-3Flag, followed by immunoprecipitation using anti-Flag antibodies. (C, D) Lifetimes of SCMV-GFP/ΔGDD and SCMV-G^6556^-GFP/ΔGDD RNA. (**C**) RNA stability assays following transcriptional inhibition with actinomycin D showed that ZmECT23 markedly accelerated the degradation of SCMV-GFP/ΔGDD genomic RNA compared with the GUS control. (**D**) In *N. benthamiana* cells heterologously expressing ZmECT23, the degradation rate of SCMV-G^6556^-GFP/ΔGDD genomic RNA was significantly delayed compared to that of SCMV-GFP/ΔGDD. Data are presented as means ± SD (*n* = 9 infiltrated leaf patches from three independent experiments). TI, transcription inhibition. (**E**) A^6556^-methylated reads carry significantly shorter poly(A) tails than unmethylated reads (probability threshold >0.5). Statistical significance was determined by Welch’s *t*-test (*P < .05). (**F**) *ZmECT23* expression level was significantly reduced in the 1 SL of CMV:*ZmECT23*-infected seedlings compared to the CMV:*GUS* (control) seedlings at 18 days VPI (*n* = 12 plants from three independent experiments). (**G**) Stronger GFP fluorescence was observed in the 1 SL of SCMV-GFP-infected maize seedlings compared to control seedlings at 7 dpi. Scale bar, 2 cm. (**H**) Percentages of maize seedlings infected by SCMV-GFP with different disease symptom grades. Disease degrees (N, I, II, III) were visually assessed at 7 dpi. *ZmECT23*-silenced seedlings infected with SCMV-GFP showed more severe mosaic symptoms, with an increased proportion of severe disease symptoms (grade III) and a decreased proportion of asymptomatic cases (grade N), relative to control plants. Data are presented as means ± SE from three independent experiments. (**I**) SCMV genomic RNA accumulation levels in the 1 SL of *ZmECT23*-silenced or control seedlings were determined through qRT-PCR. Data are expressed as the means ± SE (*n* = 11 plants from three independent experiments). (**J**) Immunoblotting analysis of SCMV CP accumulation in *ZmECT23*-silenced and control seedlings (*n* = 3 plants). The relative intensities of the CP bands were quantified using ImageJ. Statistical differences in panels (B, F) and (I, J) were determined using the two-tailed Student’s *t*-test (*P < .05; **P < .01; ***P < .001; ****P < .0001).

Given the inhibitory effect of m^6^A on A^6556^ during SCMV infection, we hypothesized that m^6^A-modified SCMV genomic RNA might be recognized by reader proteins and subsequently mediating its degradation. To test this hypothesis, we conducted a comparative evolutionary analysis of m^6^A reader proteins from maize, *A. thaliana*, and *N. benthamiana*. As shown in Supplementary Fig. S4A and B, ZmECT7, ZmECT13, and ZmECT23 are more closely related to ECT proteins in *A. thaliana* and *N. benthamiana*, suggesting them as potential functional candidates. Additionally, we found a 3.1-fold increase in *ZmECT23* expression following SCMV infection by a RNA-sequencing (RNA-seq) analysis, whereas no significant changes were observed for *ZmECT7* and *ZmECT13* (Supplementary Fig. S4C) [57]. We further found that ZmECT23 contains highly conserved sites characteristic of the YTHDF subfamily, including the aromatic cage structure, m^6^A, and RNA contact sites, as analyzed by ESPript 3 [58]. Structural prediction further revealed that three conserved tryptophan residues at positions 471, 528, and 533 in ZmECT23 form an aromatic cage for m^6^A binding, similar to that observed in other YTH domain proteins, such as YTHDF2 (Supplementary Fig. S4D and E). Furthermore, three other residues (457, 461, and 472) involved in hydrogen-bond interactions with m^6^A are also conserved between the two proteins (Fig. 3A and Supplementary Fig. S4D and E). These results suggest that ZmECT23 functions as a maize-specific m^6^A reader that may play a role in regulating SCMV infection.

We next investigated the binding of ZmECT23-3Flag to SCMV genomic RNA using RIP assays in *N. benthamiana* transient expression system. As shown in Fig. 3B, the m^6^A-modified A^6556^ site was significantly enriched in the anti-Flag antibody precipitate compared to non-methylated G^6556^ control. To determine the functional impact of this interaction, RNA stability assays were performed with replication-defective viruses SCMV-GFP/ΔGDD and SCMV-G^6556^-GFP/ΔGDD (Supplementary Fig. S3D). Time-course analysis following transcriptional inhibition with actinomycin D revealed markedly accelerated degradation of SCMV-GFP/ΔGDD genomic RNAs in ZmECT23 expressing *N. benthamiana* cells compared to GUS controls (Fig. 3C). In contrast, the degradation rate of SCMV-G^6556^-GFP/ΔGDD genomic RNA was substantially slower than that of SCMV-GFP/ΔGDD in ZmECT23-expressing cells (Fig. 3D). These results collectively demonstrate that ZmECT23 functions as an m^6^A reader protein that recognizes and destabilizes m^6^A-modified SCMV genomic RNA.

Whereas m^6^A-directed mRNA decay in mammals is mediated by the YTHDF2 protein (reader) through recruitment of the CCR4–NOT complex, a distinct mechanism in *N. benthamiana* involves the ortholog NbECT2, which promotes viral RNA degradation by recruiting UPF3 [59, 60]. We therefore investigated whether the maize ortholog, ZmECT23, associates with CCR4 or UPF3. Using Y2H assays, we found that ZmECT23 interacts with ZmCCR4, a key component of the CCR4-NOT complex (Supplementary Fig. S5A). The interaction between ZmECT23 and ZmCCR4 was further verified using LCI and pull-down assays (Supplementary Fig. S5B and C). Given that CCR4 catalyzes deadenylation and initiates deadenylation-dependent RNA decay, we examined whether m^6^A at A^6556^ is associated with poly(A) length. DRS analyses (nanopolish-polya; probability threshold >0.5) showed that A^6556^-methylated viral reads have significantly shorter poly(A) tails than unmethylated viral reads (Fig. 3E). Subsequently, we silenced expressions of *ZmECT23* in maize plants through CMV-VIGS. Compared with SCMV-infected control plants, SCMV-infected plants with silenced *ZmECT23* exhibited more severe mosaic symptoms (Fig. 3F–H). Additionally, the accumulated levels of viral genomic RNA and CP significantly increased to ~2.5- and 3.1-fold, respectively, in silenced plants compared to control plants (Fig. 3I and J). These findings indicate that the ZmECT23-ZmCCR4-NOT regulatory axis represents a key layer of host defense, where m^6^A modification marks viral RNA for targeted degradation.

### ZmeIF4A3 is a transcriptome-wide m^6^A suppressor in maize plants

**Figure 4.**
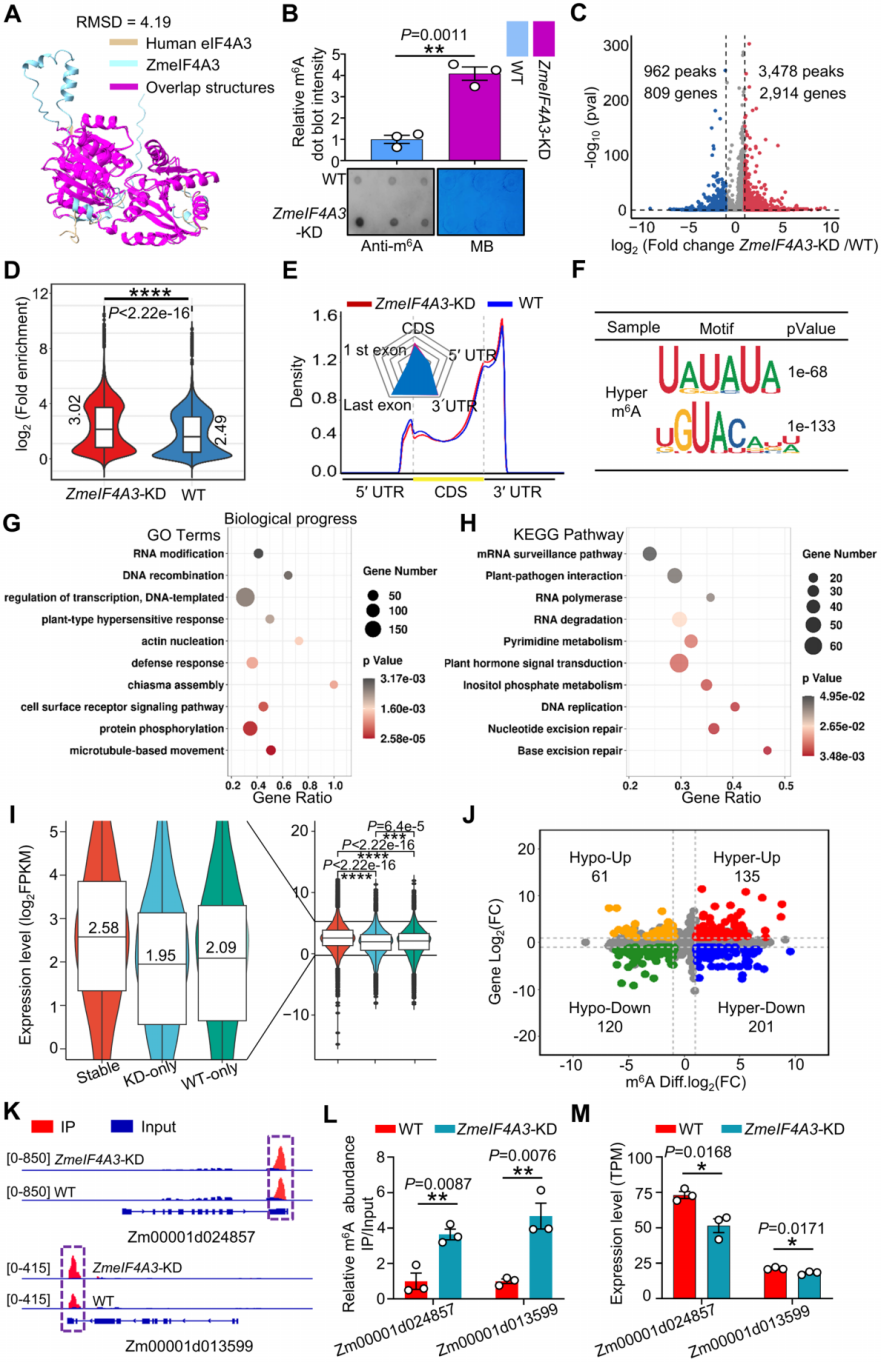
*ZmeIF4A3*-KD maize plants exhibit transcriptome-wide m^6^A hypermethylation. (**A**) Core helicase domain of ZmeIF4A3 and human eIF4A3 showed structural conservation. Crystal structure of the HseIF4A3 (PDB: 2HYI) was aligned with the AlphaFold2-predicted model of ZmeIF4A3 (A0A1D6HQ89). Regions of conserved structure, as indicated by low RMSD values, are highlighted in magenta. (**B**) Dot blot analysis revealed that the m^6^A modification levels in *ZmeIF4A3-*KD plants were significantly elevated relative to those in WT plants. MB staining was used as a loading control for total RNAs. Grayscale value was visualized using ImageJ software. Data from three independent experiments were subjected to statistical analysis using two tailed Student’s *t*-test (**P < .01). (**C**) A volcano plot illustrated the changes in m^6^A modification levels between *ZmeIF4A3-*KD and WT plants. (**D**) A violin plot illustrated the log_2_(fold enrichment) of m^6^A peaks in *ZmeIF4A3-*KD and WT plants. The median value was significantly higher in *ZmeIF4A3*-KD plants (3.02) than in WT plants (2.49) (Wilcoxon rank-sum test, *P* < 2.22 × 10^−16^). (**E**) Pie chart and radar plot showed m^6^A peak distribution in the indicated gene regions from the WT and *ZmeIF4A3-*KD plants. (**F**) Representative sequence motif identified from the 1000 most significant m^6^A peaks in the *ZmeIF4A3-*KD plants. 5′ UTR, 5′ untranslated region; CDS, coding sequence; 3′ UTR, 3′ untranslated region. (**G, H**) GO and KEGG enrichment analysis of the EHMGs (*ZmeIF4A3*-KD versus WT). The top 10 significantly enriched GO terms (G) and significantly enriched KEGG pathways (H) are presented. The size of the circles indicates the number of enriched transcripts, and the color indicates the enrichment significance –log_10_ (*P*-value). (**I**) Violin plots depicted the distribution of expression level of genes encoding mRNAs with stable, KD-only and WT-only m^6^As. Stable m^6^As are defined as m^6^A peaks that are consistently present in both WT and *ZmeIF4A3*-KD plants; KD-only and WT-only m^6^As are those peaks that are uniquely found in *ZmeIF4A3*-KD or WT plants, respectively. The left panel shows a magnified view of the *y*-axis (0–5). Median values are 2.58 (stable), 1.95 (KD-only) and 2.09 (WT-only). Statistical significance was determined by Wilcoxon rank sum test (***P < .001; ****P < .0001). (**J**) Quadrant diagram represented the integrated analysis of changes in m^6^A peaks (*x-*axis) and mRNA expression levels (*y-*axis) in WT versus *ZmeIF4A3*-KD plants. Colored points on the graph denote genes exhibiting significant alterations in both transcription and methylation levels. hypo-down, m^6^A peak downregulated, mRNA downregulated; hypo-up, m^6^A peak downregulated, mRNA upregulated; hyper-down, m^6^A peak upregulated, mRNA downregulated; hyper-up, m^6^A peak upregulated, mRNA upregulated. The numbers in the quadrants represent the count of peaks within each respective quadrant. (**K**) Integrative genomics viewer showed the m^6^A-seq results on *Zm00001d024857* and *Zm00001d013599* transcripts in both *ZmeIF4A3-*KD and WT plants. The purple dashed boxes indicate m^6^A peaks positions within both the Input and IP samples. (**L**) MeRIP-qPCR showed the relative m^6^A levels of *Zm00001d024857* and *Zm00001d013599* in WT and *ZmeIF4A3-*KD plants. Values are means ± SE (two-tailed Student’s *t* test, *n* = 3 independent experiments, **P < .01). (**M**) RNA-seq data showed the relative expression levels of *Zm00001d024857* and *Zm00001d013599* in WT and *ZmeIF4A3*-KD plants. Values are means ± SE (two-tailed Student’s *t* test, *n* = 3 independent experiments, *P < .05).

A previous study shows that the human homolog of ZmeIF4A3, known as HseIF4A3, functions as an m^6^A suppressor in mammalian cells [20]. Intriguingly, an interaction between the SCMV-encoded NIa-Pro and ZmeIF4A3 was identified in our prior TurboID-based proximity labeling analysis [41]. We speculate that SCMV might counteract the antiviral role of the ZmECT23-ZmCCR4-NOT complex by recruiting ZmeIF4A3 via NIa-Pro. To investigate this, we first assessed the role of ZmeIF4A3 in regulating m^6^A modifications. Three-dimensional structure superposition analysis indicated highly conserved folds and conformations between ZmeIF4A3 (A0A1D6HQ89) and HseIF4A3 (PDB: 2HYI) (Fig. 4A and Supplementary Fig. S6A–C). Multiple sequence alignment further revealed highly conserved motifs of eIF4A3 across maize and other species (Supplementary Fig. S6D). Next, a C-to-T substitution in the second exon of *ZmeIF4A3* was identified from an ethyl methanesulfonate (EMS)-mutagenized maize library. This substitution led to a premature translational termination (Supplementary Fig. S7A and B). No viable homozygous progeny (T/T) were obtained from self-crossing heterozygote (C/T) plants, indicating embryonic lethality upon complete loss of ZmeIF4A3 function (Supplementary Fig. S7C and D), consistent with the essential role of eIF4A3 in maintaining embryonic stem cell identity in mammals and zebrafish [61, 62]. *ZmeIF4A3-like* (Zm00001d051840) is a conserved homolog and shares 85% and 99% identity at the cDNA and amino acid sequences to those of *ZmeIF4A3*, respectively. However, its expression levels are only 26% of *ZmeIF4A3* in wild-type (WT) plants (Supplementary Fig. S8A–C), indicating an insufficient compensation to rescue embryonic lethality. Furthermore, we obtained a knockdown line (*ZmeIF4A3*-KD) with a *Mutator* insertion 24 bp upstream of the *ZmeIF4A3* start codon (Supplementary Fig. S7E and F), reducing the level of *ZmeIF4A3* transcripts by 30% but up-regulating the accumulated transcript levels of *ZmeIF4A3-like* (Supplementary Figs S7G and S8D).

Comparing transcriptomic profiles of WT and *ZmeIF4A3*-KD plants (Supplementary Fig. S9 and Supplementary Table S4) enabled us to identify 3258 differentially expressed genes (DEGs) (*P* < .05, fold enrichment > 2), with 1684 up-regulated and 1574 down-regulated DEGs. GO enrichment revealed significant over-representation of genes involved in chromatin assembly, nucleosome positioning, DNA packaging, and metabolic processes (including glucan, glycogen, and small-molecule metabolism). The gene expression changes of eight DEGs, selected at random from these categories, were further tested by qRT-PCR and agreed well with the RNA-seq results (Supplementary Fig. S9E–H).

Dot blot assays revealed the significantly elevated total levels of m^6^A in *ZmeIF4A3*-KD compared to that of WT plants (Fig. 4B). We further identified 3478 hyper-methylated m^6^A peaks corresponding to 2914 genes (*P* < .05, fold enrichment > 2), and 962 hypo-methylated peaks corresponding to 809 genes in *ZmeIF4A3-*KD (Fig. 4C and Supplementary Table S5). Consistent with these findings, the *ZmeIF4A3*-KD plants exhibited transcriptome-wide m^6^A accumulation (Fig. 4D). Metagene profiling revealed that hyper-methylated m^6^A peaks were predominantly enriched in the 3′ UTR and the coding sequences near the stop codon (Fig. 4E). The two motifs, UAUAUA and UGUAC, which were predominantly present in hyper-methylated peaks, were consistent with the previously reported URUAY motif [63] (Fig. 4F). These results suggest that knockdown of *ZmeIF4A3* induces global m^6^A hyper-methylation but does not affect the recognition patterns of modification sites. We were particularly interested in the genes regulated by eIF4A3, and in *ZmeIF4A3*-KD plants, the methylation levels of these genes should be upregulated. Therefore, we performed comprehensive GO and KEGG enrichment analyses on eIF4A3-regulated hypermethylated m^6^A-containing genes (hereafter referred to as EHMGs). GO analysis indicated that EHMGs were enriched in biological processes associated with microtubule-based movement, protein phosphorylation, and cell surface receptor signaling pathway (Fig. 4G). Moreover, KEGG pathway analysis revealed that EHMGs were significantly enriched in pathways related to DNA damage repair, DNA replication, and plant hormone signal transduction (Fig. 4H).

To explore the interplay between RNA methylation dynamics and gene expression changes, we categorized m^6^A peaks into three groups: “Common m^6^As”, “WT-only,” and “KD-only” (Supplementary Table S6). Notably, transcripts associated with KD-only displayed significantly lower expression levels than those with WT-only (Fig. 4I). Integrated m^6^A-seq and RNA-seq data identified transcripts from 336 genes exhibiting hyper-methylated peaks (*P* < .05, fold enrichment > 4). Among these, transcript levels of 201 genes were downregulated, while those from 135 genes were upregulated. Conversely, transcripts from 181 genes with hypo-m^6^A methylation were identified, with expression levels of 61 genes upregulated and of 120 genes downregulated (Fig. 4J and Supplementary Table S7). For instance, increased m^6^A peaks were observed in the exon of *Zm00001d024857* and the 3′ UTR of *Zm00001d013599*, two genes implicated in mRNA surveillance (Fig. 4K). MeRIP-qPCR confirmed hyper-methylation of these transcripts in *ZmeIF4A3*-KD compared to WT (Fig. 4L), and the expression of these genes significantly reduced in *ZmeIF4A3*-KD plants (Fig. 4M). Collectively, these findings indicate that ZmeIF4A3 functions as an m^6^A suppressor, inhibiting the decay of specific transcripts with m^6^A modification.

### ZmeIF4A3 acts as a pro-viral factor during SCMV infection

**Figure 5.**
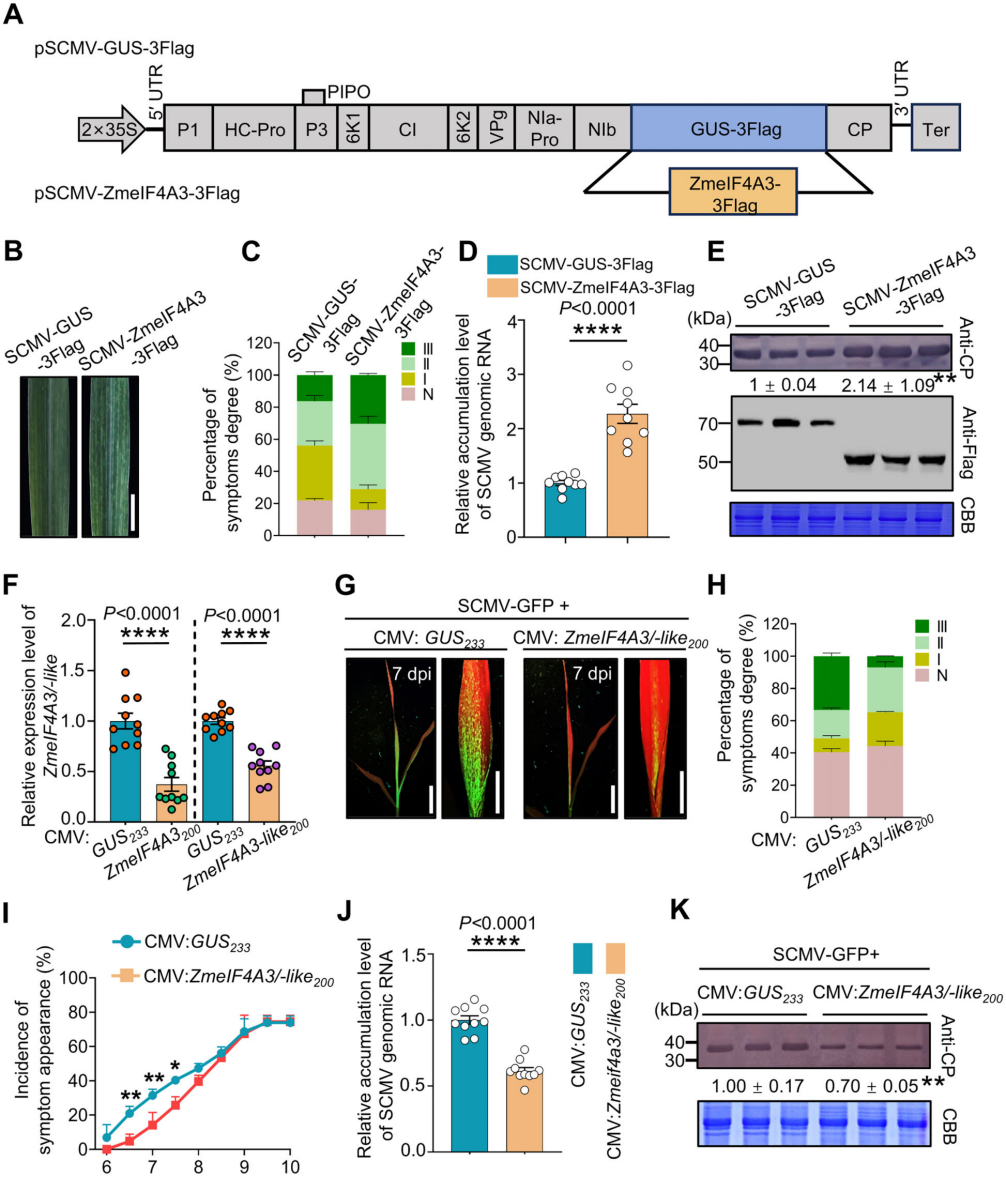
ZmeIF4A3 enhances SCMV infection. (**A**) Schematic diagrams of pSCMV-GUS-3Flag and pSCMV-ZmeIF4A3-3Flag. (**B**) Stronger mosaic symptoms were observed in the 1 SL of SCMV-ZmeIF4A3-3Flag-infected plants compared to SCMV-GUS-3Flag-infected (control) plants. Photographs were taken at 5 dpi. Scale bar, 2 cm. (**C**) Percentages of maize plants infected by SCMV-GUS-3Flag or SCMV-ZmeIF4A3-3Flag with different disease symptom grades at 5 dpi. (**D, E**) Relative viral genomic RNA and CP accumulation levels were higher in SCMV-ZmeIF4A3-3Flag-infected plants compared to control plants (*n* = 9 plants from three independent experiments), as determined by qRT-PCR and immunoblotting analysis. (**F**) Relative expression levels of *ZmeIF4A3/ZmeIF4A3-like* were reduced in *ZmeIF4A3/ZmeIF4A3-like*-silenced plants (*n* = 9 plants from three independent experiments) using qRT-PCR. (**G**) Reduced GFP fluorescence was observed in the 1 SL of SCMV-GFP-infected plants compared to control (CMV:*GUS*) plants at 7 dpi. Scale bars, 2 cm. (**H**) Percentages of maize plants infected by SCMV-GFP with different disease symptom grades. Data are presented means ± SE, derived from three independent experiments. (**I**) Symptom progression on individual plant was recorded from 6 to 10 dpi. In this study, 43 CMV:*ZmeIF4A3*-infected plants (three experiments) and 57 CMV:*GUS*-infected plants (three experiments) were inoculated by SCMV-GFP. The values represent the means ± SE. (**J, K**) SCMV genomic RNA and CP accumulation in the 1 SL of *ZmeIF4A3/ZmeIF4A3-like*-silenced or control plants (*n* = 9 plants from three independent experiments) were determined through qRT-PCR and immunoblotting analysis. Relative intensities of the CP bands were quantified using ImageJ software. CBB-stained gels were used to show sample loadings. In panels (D–F) and (I–K), data are presented as means ± SE (two-tailed Student’s *t* test, *P < .05; **P < .01; ***P < .001; ****P < .0001).

To elucidate the role of ZmeIF4A3 in SCMV infection, we transiently expressed ZmeIF4A3 in maize plants using an SCMV infectious clone, designated pSCMV-ZmeIF4A3-3Flag, with SCMV-GUS-3Flag serving as the control (Fig. 5A). Maize plants infected with pSCMV-ZmeIF4A3-3Flag exhibited more severe mosaic symptoms compared to those infected with SCMV-GUS-3Flag (Fig. 5B and C). We found a 2.3-fold increase in viral genomic RNA and a 2.1-fold increase in CP in plants infected with SCMV-ZmeIF4A3-3Flag relative to control plants (Fig. 5D and E), suggesting that ZmeIF4A3 functions as a susceptibility factor in facilitating SCMV infection.

In parallel, we infected WT and *ZmeIF4A3-*KD seedlings with SCMV-GFP. Both WT and *ZmeIF4A3-*KD plants showed severe mosaic symptoms at 5 dpi (Supplementary Fig. S10A), and no significant differences in the accumulated levels of SCMV genomic RNA and CP as detected by qRT-PCR and immunoblotting, respectively (Supplementary Fig. S10B and C). Given an ∼22% increase of *ZmeIF4A3-like* transcripts in *ZmeIF4A3*-KD plants (Supplementary Fig. S8D) and a high similarity between ZmeIF4A3 and ZmeIF4A3-like, we hypothesized that both proteins may suppress m^6^A modification on SCMV genomic RNA. To address this hypothesis, we employed the CMV-VIGS to silence both genes. We achieved 63% and 45% reduction in transcript levels of *ZmeIF4A3* and *ZmeIF4A3-like* respectively, relative to CMV:*GUS* control plants (Fig. 5F). Silencing of *ZmeIF4A3* and *ZmeIF4A3-like* led to fewer GFP fluorescent spots upon SCMV infection (Fig. 5G), a higher percentage of symptom-free plants (grade N), and a lower percentage of plants with severe disease symptoms (grade III) (Fig. 5H). Infection rates were also reduced in *ZmeIF4A3/ZmeIF4A3-like* -silenced plants compared to control plants from 5 to 8 dpi (Fig. 5I), with a ∼40% reduction in SCMV genomic RNA and ∼30% in CP in the upper leaves of silenced plants (Fig. 5J and K). Furthermore, in *ZmeIF4A3-*KD plants with additional silencing of *ZmeIF4A3-like*, GFP fluorescence was significantly reduced compared to CMV:*GUS* control plants, with SCMV genomic RNA and CP levels decreased to 56% and 43%, respectively (Supplementary Fig. S10D–G). Collectively, these results indicate that both ZmeIF4A3 and ZmeIF4A3-like facilitate SCMV infection.

### ZmeIF4A3 is associated with SCMV VRCs via binding with NIa-Pro

**Figure 6.**
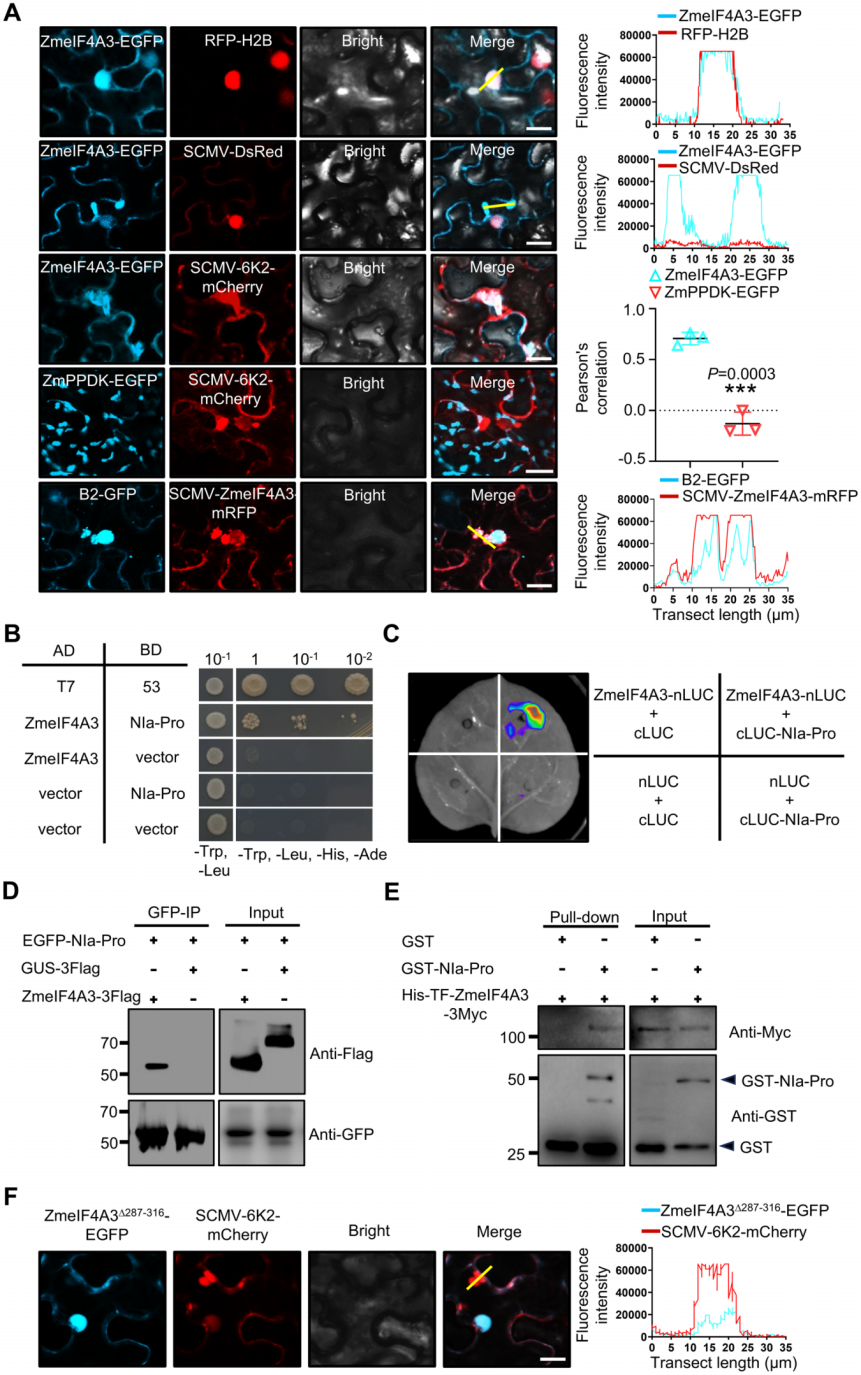
ZmeIF4A3 is recruited into SCMV VRCs via direct binding with NIa-Pro. (**A**) Subcellular localization of ZmeIF4A3-GFP in the leaf cells of H2B-RFP transgenic *N. benthamiana* by confocal microscopy at 3 days post-infiltration. Co-localization analysis of ZmeIF4A3-GFP with SCMV-DsRed or SCMV-6K2-mCherry, and ZmPPDK-GFP (negative control) with SCMV-6K2-mCherry in the leaf cells of *N. benthamiana*. The 6K2-mCherry served as a marker for SCMV VRCs. Confocal images of SCMV-ZmeIF4A3-mRFP in B2-GFP (a marker that binds double-stranded RNA (dsRNA) and labels active viral replication sites) transgenic *N. benthamiana* leaf cells at 5 days post-infiltration. Scale bars, 10 μm. The yellow line segment delineates the region for fluorescence intensity measurement. The line graph illustrates relative fluorescence intensity, with the *y*-axis depicting the relative fluorescence intensities of GFP or RFP and the *x*-axis representing transect length in micrometers. The blue line corresponds to fluorescence induced by ZmeIF4A3-GFP or B2-GFP, while the red line indicates fluorescence induced by H2B-RFP, SCMV-DsRed, or SCMV-ZmeIF4A3-mRFP. The graph shows Pearson’s correlation coefficients derived from three images, indicating significant co-localization between ZmeIF4A3-GFP and SCMV-6K2-mCherry. Data are presented as means ± SE (Student’s *t*-test; ***P < .001). (**B**) Y2H assays showed the interaction between ZmeIF4A3 and NIa-Pro. (**C**) LCI assay confirmed the ZmeIF4A3–NIa-Pro interaction. *Agrobacterium* strain GV3101 containing the LUC constructs was co-infiltrated into *N. benthamiana* leaves. Images of infiltrated leaves were captured after three days of incubation. (**D**) Co-IP analysis showed the interactions between SCMV-encoded NIa-Pro with ZmeIF4A3. *N. benthamiana* leaves were co-infiltrated with *A. tumefaciens* cells harboring expression vectors. Leaf protein extracts were incubated with anti-GFP nanobody agarose beads. Samples before (Input) and after (IP) were analyzed by immunoblotting using anti-GFP or Flag antibodies. (**E**) GST pull-down assays confirmed the direct interaction between SCMV NIa-Pro and ZmeIF4A3. Black arrowheads indicate GST-NIa-Pro or GST. (**F**) Deletion of 287–316 region (ZmeIF4A3^Δ287-316^) abolished co-localization with SCMV-6K2-mCherry in *N. benthamiana* leaf cells. Photographs were taken at 5 days post-infiltration. Scale bar, 10 μm.

Next, we set to investigate the molecular mechanisms underlying the proviral role of ZmeIF4A3 in SCMV infection. We first tested the subcellular localization of ZmeIF4A3 in the absence or presence of SCMV. ZmeIF4A3-EGFP localized to both the nucleus and cytoplasm in *N. benthamiana* cells, consistent with prior reports (Fig. 6A) [18]. Strikingly, in cells infected with SCMV-DsRed, ZmeIF4A3-EGFP formed irregular cytoplasmic aggregates (Fig. 6A), indicative of an association with VRCs. To probe this possibility, we co-expressed ZmeIF4A3-EGFP or chloroplast-localized pyruvate orthophosphate dikinase (ZmPPDK-GFP) [64] with SCMV-6K2-mCherry in *N. benthamiana* leaf cells [65]. It needs to note that 6K2-mCherry serves as a marker for SCMV VRCs. Confocal microscopy revealed pronounced co-localization of ZmeIF4A3-EGFP with 6K2-mCherry in aggregate structures, whereas ZmPPDK-GFP remained chloroplast-localized and did not co-localize with 6K2-mCherry (Fig. 6A and Supplementary Fig. S11). We also used B2-GFP, which binds replicative RNA intermediates, double-stranded RNA (dsRNA), and has been shown to label active SCMV VRCs [66]. As shown in Fig. 6A, B2-GFP and ZmeIF4A3 co-localized well, suggesting that ZmeIF4A3 was associated with viral replication sites or VRCs. Collectively, these observations establish that ZmeIF4A3 is likely recruited to VRCs during SCMV infection, providing a molecular basis for its role in modulating SCMV pathogenesis.

NIa-Pro, a component of VRCs, was previously identified as a potential interacting partner of ZmeIF4A3 [41]. We hypothesized that it mediates the incorporation of ZmeIF4A3 into VRCs. Y2H assays confirmed an interaction between ZmeIF4A3 and NIa-Pro (Fig. 6B), which was further validated by LCI and Co-IP assays (Fig. 6C and D). Moreover, GST pull-down assays affirmed the direct binding between NIa-Pro and ZmeIF4A3 (Fig. 6E). Similarly, ZmeIF4A3-like also exhibited interaction with NIa-Pro (Supplementary Fig. S12). LCI and Co-IP assays pinpointed the HELICc domain of ZmeIF4A3 (residues 287–368) as the region interacting with NIa-Pro *in planta* (Supplementary Fig. S13A–C), with further precision mapping to residues 287–316 (Supplementary Fig. S13D). Deletion of this region (ZmeIF4A3^Δ287-316^) abolished its co-localization with SCMV-6K2-mCherry, indicating that the NIa-Pro interaction is crucial for ZmeIF4A3’s integration into VRCs (Fig. 6F). Significantly, the ZmeIF4A3^∆287-316^ mutation also reduced the accumulated levels of SCMV genomic RNA and CP compared with ZmeIF4A3 (Supplementary Fig. S13E-S13G).

To ascertain that NIa-Pro directly recruits ZmeIF4A3 to 6K2-VPg-Pro-induced VRCs, we co-expressed NIa-Pro-3Flag or GUS-3Flag (as a control) with ZmeIF4A3-EGFP and 6K2-VPg-Pro-mCherry in *N. benthamiana* cells. Confocal microscopy revealed that ZmeIF4A3-EGFP localized to the nucleus and cytoplasm without co-localizing with 6K2-VPg-Pro-mCherry (Supplementary Fig. S14A). However, co-expression with NIa-Pro-3Flag resulted in remarkable co-localization of ZmeIF4A3-EGFP with 6K2-VPg-Pro-mCherry (Supplementary Fig. S14A). Notably, NIa-Pro_1-194_-3Flag, a variant deficient in interaction with ZmeIF4A3, did not cause ZmeIF4A3 co-localization with 6K2-VPg-Pro-mCherry, retaining ZmeIF4A3 in the nucleus and cytoplasm (Supplementary Fig. S14A–C). Together, these findings confirm that NIa-Pro directly recruits ZmeIF4A3 into VRCs, thereby facilitating viral infection.

### ZmeIF4A3 binds to SCMV genomic RNA to inhibit ZmMTA-medicated m^6^A methylation and promotes viral genomic replication

To further understand the role of ZmeIF4A3 within VRCs, we first examined whether it enhanced viral translation. SUnSET and polysome profiling revealed a marked global reduction in host translation in *ZmeIF4A3*-KD plants (Supplementary Fig. S15A and B). Furthermore, we found that during infection with replication-defective SCMV-GFP/ΔGDD, overexpression of ZmeIF4A3 did not significantly enhance the translational efficiency of SCMV-GFP/ΔGDD, despite increasing overall host translation rates (Supplementary Fig. S15C–E). Collectively, these results strongly suggest that ZmeIF4A3 is not involved in the translation of SCMV.

**Figure 7.**
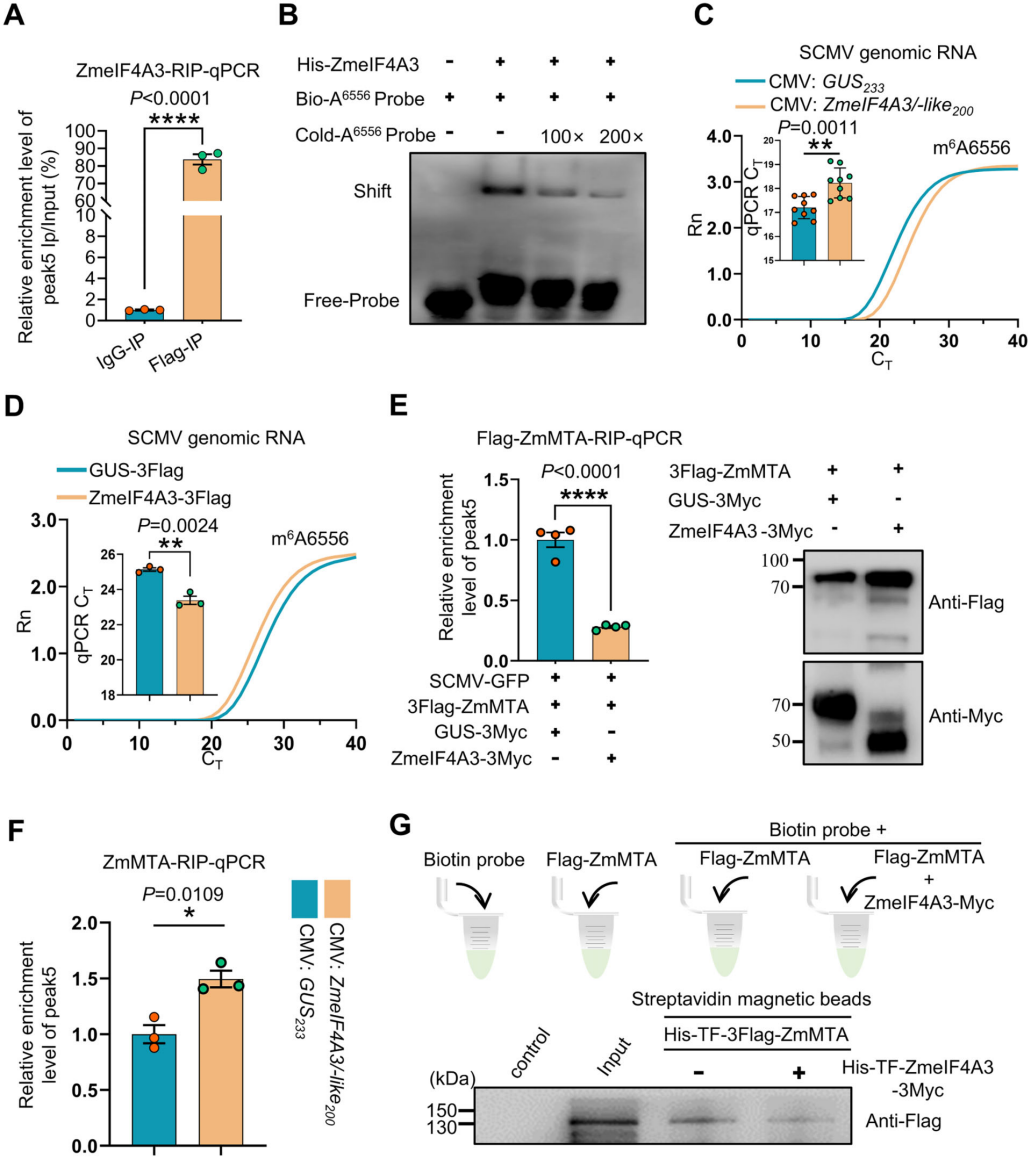
ZmeIF4A3 binds to SCMV genomic RNA and reduces its m^6^A methylation levels by hindering the attachment of ZmMTA. (**A**) RIP-qPCR assays revealed the binding of ZmeIF4A3 to the peak5 region. Protein-RNA complex was extracted from *N. benthamiana* leaves (*n* = 3 independent experiments) co-infiltrated with SCMV-GFP and ZmeIF4A3-3Flag, followed by immunoprecipitation with anti-Flag or mouse IgG (negative control). (**B**) EMSA results indicated that ZmeIF4A3 bound to the peak5 region of SCMV genomic RNA. The results showed that ZmeIF4A3 was capable of binding to the biotin-labeled probe, and the unlabeled probe could competitively interfere with the biotin-labeled probe binding to ZmeIF4A3. (**C**) SELECT-qPCR analysis revealed significantly elevated m^6^A levels of A^6556^ in *ZmeIF4A3/ZmeIF4A3-like*-silenced (CMV:*ZmeIF4A3/ZmeIF4A3-like*) plants compared to CMV:*GUS*-silenced (control) plants. Values are presented as means ± SD (*n* = 9 plants from three independent experiments, two-tailed Student’s *t* test). (**D**) SELECT-qPCR analysis showed that overexpression of ZmeIF4A3-3Flag significantly reduced the m^6^A modification levels at the A^6556^ of the SCMV genomic RNA (*n* = 3 independent experiments). (**E**) ZmeIF4A3 inhibits ZmMTA binding to SCMV peak5 region, as determined by Flag-ZmMTA-RIP-qPCR assays. pSCMV-GFP and 3Flag-ZmMTA were agroinfiltrated into *N. benthamiana* leaves (*n* = 4 independent experiments) along with GUS-3Myc or ZmeIF4A3-3Myc. Immunoblotting confirmed the expression of ZmMTA-3Flag, GUS-3Myc, and ZmeIF4A3-3Myc. Molecular weight markers are shown on the left. (**F**) ZmMTA-RIP-qPCR analysis revealed significantly enhanced ZmMTA occupancy on SCMV genomic RNA in CMV:*ZmeIF4A3/ZmeIF4A3-like-*infected plants compared to CMV:*GUS*-infected (control) plants. Data are means ± SE from three independent experiments. (**G**) ZmeIF4A3 inhibits ZmMTA binding to peak5 of SCMV genomic RNA validated by the biotinylated RNA pull-down assays. Bio-A^6556^ probe was incubated with His-TF-3Flag-ZmMTA, or with His-TF-3Flag-ZmMTA and His-TF-ZmeIF4A3-3Myc together. Components pulled down using streptavidin beads were detected by immunoblotting with anti-Flag antibody. In panels (A), (D), (E), and (F), values are presented as means ± SE (two-tailed Student’s *t* test, *P < .05; **P < .01; ***P < .001; ****P < .0001).

We next investigated whether ZmeIF4A3 can bind viral genomic RNA and suppress m^6^A modification, thereby preventing viral RNA degradation. RIP-qPCR analysis revealed that ZmeIF4A3 interacted with peak5 of the SCMV genomic RNA, which includes the GGA^6556^CA motif targeted by m^6^A modifications (Fig. 7A). This binding was confirmed by EMSA, where biotin-labeled probes corresponding to peak5 of ZmeIF4A3 was shifted when incubated with His-ZmeIF4A3 (Fig. 7B) and this shift was effectively competed by unlabeled probes (Fig. 7B). A significant increase in m^6^A modification was found in SCMV-infected maize plants with both *ZmeIF4A3* and *ZmeIF4A3-like* silenced (CMV:*ZmeIF4A3/ZmeIF4A3-like*), in comparison to control plants (CMV:*GUS*) (Fig. 7C). Conversely, m^6^A levels at A^6556^ were significantly reduced in ZmeIF4A3-3Flag-IP samples compared to GUS-3Flag-IP controls (Fig. 7D), confirming that ZmeIF4A3 inhibited m^6^A modification at this site.

Additionally, we found that ZmeIF4A3 competed with ZmMTA and inhibited its binding to SCMV peak5 in *N. benthamiana*. As shown by using RIP-qPCR, we found that ZmeIF4A3 interfered with ZmMTA’s binding to the RNA (Fig. 7E). Furthermore, in SCMV-infected maize plants where both *ZmeIF4A3* and *ZmeIF4A3-like* were silenced (CMV:*ZmeIF4A3/ZmeIF4A3-like*), we detected increased ZmMTA occupancy on SCMV genomic RNA (Fig. 7F). This finding was further validated by *in vitro* RNA pull-down assays, where the addition of recombinant ZmeIF4A3 diminished association of ZmMTA with a biotinylate-labeled SCMV genomic RNA fragment encompassing A^6556^ (Fig. 7G). Together, these findings indicate that ZmeIF4A3 suppresses ZmMTA-mediated m^6^A methylation at A^6556^, thereby contributing to the stabilization of SCMV genomic RNA and simultaneously facilitating viral replication.

### EIF4A3-mediated m^6^A suppression promotes potyviral infection

To test whether eIF4A3-mediated suppression of m^6^A modification is conserved among other potyviruses, we first generated *N. benthamiana* transgenic lines expressing ZmeIF4A3 (*ZmeIF4A3*-TE) (Supplementary Fig. S16A–C). These *ZmeIF4A3*-TE lines showed decreased m^6^A modification relative to non-transgenic *N. benthamiana*, reinforcing the role of eIF4A3 as an m^6^A suppressor (Supplementary Fig. S16D).

Moreover, these transgenic lines exhibited enhanced susceptibility to other potyviruses, including PVY and TuMV, as evidenced by significantly higher levels of GFP fluorescence intensity, viral genomic RNA accumulation, and GFP protein expression compared to non-transgenic plants (Supplementary Fig. S17A–C and G–I). In contrast, silencing of *NbeIF4A3* using TRV-induced gene silencing resulted in a 65% or 60% reduction in PVY and TuMV genomic RNA levels, respectively, and a 40% and 90% decrease in GFP accumulation relative to control plants (Supplementary Fig. S17D–F and J–L). Notably, in *NbeIF4A3*-silenced plants, the m^6^A levels in the RNA corresponding to the TuMV *CP* were significantly elevated compared to control plants (Supplementary Fig. S17M). Collectively, these data revealed that potyviruses employ a conserved strategy of recruiting the m^6^A suppressor eIF4A3 to inhibit m^6^A modification and thereby prevent viral RNA degradation during infection.

## Discussion

In eukaryotic cells, the m^6^A modification is crucial for RNA metabolism and viral life cycles, with recent evidence highlighting its role in antiviral responses [10, 11, 29, 30]. However, the strategies by which viruses overcome m^6^A-mediated host defenses are not well characterized. Our study reveals that the ZmECT23-ZmCCR4-NOT complex targets m^6^A-marked viral RNA for degradation, thereby suppressing SCMV infection. Notably, we uncover a functional role for eIF4A3 as an m^6^A suppressor, modulating m^6^A modifications in both host and viral RNAs. NIa-Pro, a key component of VRCs, with a putative role in viral RNA biogenesis [67], recruits ZmeIF4A3 into VRCs to reduce m^6^A modification of SCMV genomic RNA, protecting it from degradation. These findings highlight the role of ZmeIF4A3 in regulating m^6^A modifications during viral infection and shed light on how pathogens disrupt the m^6^A pathway to evade RNA-based defenses and to establish persistent infections.

In this study, we identified multiple m^6^A peaks in SCMV genomic RNA, including regions encoding *P3N-PIPO, 6K2, VPg, NIa-Pro*, and *CP*. These findings align with prior studies indicating that multiple m^6^A modification sites are a common feature of potyviruses [34]. Notably, m^6^A enrichment patterns exhibited virus-specific variation. By integrating bioinformatics predictions with SELECT analysis, we pinpointed A^6556^ as the predominant m^6^A modification site in SCMV genomic RNA, located within the *NIa-Pro* coding region, which displayed the highest m^6^A abundance across all peaks. DRS analysis revealed that the methylation rate at A^6556^ in the SCMV genomic RNA was ~1.1% in infected maize leaves. We propose that the low m^6^A modification level at this site is primarily attributable to a pathogenic mechanism whereby the SCMV-encoded NIa-Pro recruits the maize factor ZmeIF4A3 to bind near A^6556^, which suppresses m^6^A deposition and counteracts ZmECT23-mediated RNA decay. This active recruitment mechanism is likely the main cause of the low m^6^A level observed at A^6556^ in SCMV-infected maize leaves. Other factors may also contribute to the observed low modification level, including the limited sequencing depth of DRS, the suboptimal applicability of current training models to viral RNAs, and potential dilution of viral signals since RNA was extracted from infected maize tissue rather than purified virus. Additionally, m^6^A modification is a reversible process, and demethylase activity may further modulate methylation levels. Notably, such low level of modification is consistent with reports for other viruses. For example, in HCV only ∼0.16% of adenosines are m^6^A-modified (implying ∼6% of genomes carry m^6^A) [68], and similarly low (∼1%–5%) levels on HIV, ZIKV, and HBV RNAs [32, 69–71].

Interestingly, divergent m^6^A localization was observed in TuMV, another member of the *potyvirus* genus, with enrichment predominantly localized to the *CP* coding region rather than *NIa-Pro* [35]. Despite conserved genomic organization and shared writer-recognized DRACH motifs, this divergence in m^6^A distribution may reflect context-dependent factors such as RNA-binding proteins (RBPs) and RNA secondary structures. RBPs such as TARBP2 recruit methyltransferase complexes to specific RNA regions to promote methylation [72]. Structural studies further indicate that METTL3-METTL14 complex-mediated methylation depends not only on the DRACH consensus motif but also on RNA structural accessibility, favoring unstructured regions [73]. Similarly, erasers (e.g. FTO, ALKBH5) and reader proteins exhibit RNA structure-dependent binding efficiency [74]. Therefore, further investigation of site-specific m^6^A deposition at distinct genomic loci will provide critical insights into RNA modification biology.

Although m^6^A modifications have been detected in potyviral genomic RNA, their functional roles and regulatory mechanisms remain elusive [34]. Here, we uncover an m^6^A-mediated antiviral mechanism during SCMV infection, revealing that host-imposed m^6^A modifications accelerate viral RNA degradation to restrict pathogenesis. We establish that ZmMTA catalyzes m^6^A deposition at position A^6556^ on SCMV genomic RNA, while the m^6^A reader protein ZmECT23 recognizes and destabilizes methylated viral RNA. Rapid RNA degradation mediated by reader proteins has been previously reported [75, 76]. Notably, we found that ZmECT23 directly recruits the ZmCCR4, which mediated the degradation of m^6^A-modified SCMV genomic RNA through deadenylation. While the mammalian YTHDF2 reader interacts with CNOT1 to recruit the CCR4-NOT complex for deadenylation [59], the ZmECT23-ZmCCR4-NOT axis represents a previously unrecognized degradation pathway that bypasses the canonical CNOT1 scaffold. This mechanism may operate more efficiently than the YTHDF2-dependent system, as the direct coupling of m^6^A recognition (ZmECT23) and deadenylase activation (ZmCCR4) enables rapid viral RNA clearance, suggesting evolutionary optimization of m^6^A-mediated antiviral defenses in plants.

EIF4A3 is well-characterized in mammals for its roles in mRNA splicing, translation, degradation, and m^6^A modification [13–15, 20–22]. However, the role of eIF4A3 in plants and its involvement in stress responses remain poorly understood. In our study, we identified ZmeIF4A3 as an m^6^A suppressor and demonstrated its crucial role in plant-virus interactions. Through integrated genetic and molecular biology approaches, we observed a significant increase in genome-wide m^6^A modification levels in *ZmeIF4A3*-KD plants, suggesting evolutionary conservation of eIF4A3’s tertiary structure and its role in m^6^A suppression [20]. Unlike mammals, maize has a homolog, *ZmeIF4A3-like*, which partially compensates for ZmeIF4A3 functions. However, complete loss of ZmeIF4A3 function causes embryonic lethality, indicating insufficient functional redundancy and highlighting ZmeIF4A3’s dominance in suppressing m^6^A modifications. RNA-seq analysis further revealed a widespread disruption of nuclear functions, such as chromatin assembly and nucleosome positioning, in *ZmeIF4A3*-KD plants. This impairment of nuclear structure maintenance and gene regulatory functions likely contributes to the observed embryonic lethality. These findings broaden our understanding of eIF4A3 in plants by linking its epitranscriptomic role directly to embryogenesis.

Interestingly, we observed hyper-methylation in only a subset of transcripts in *ZmeIF4A3*-KD plants, suggesting that ZmeIF4A3 may selectively suppress m^6^A deposition. We hypothesize that this selectivity could be attributed to the spatial proximity between eIF4A3 and specific m^6^A sites. As a core component of the EJC, eIF4A3 binds to pre-mRNA ~24 nucleotides upstream of exon-exon junctions and remains deposited on mRNA after splicing [12]. If an m^6^A site is located within such a region, the binding of eIF4A3 could sterically hinder the recruitment of m^6^A writer complexes, thereby reducing modification efficiency at specific sites. In contrast, m^6^A sites distant from EJC binding sites are likely regulated by distinct mechanisms. This spatial constraint may account for the selective role of eIF4A3 in modulating m^6^A methylation. Furthermore, our findings demonstrated that ZmeIF4A3 also suppresses m^6^A modification on viral RNA. Specifically, we found that ZmeIF4A3 binds to the SCMV genomic RNA and reduces m^6^A levels at site A^6556^. SCMV is a positive-sense single-stranded RNA virus that utilizes a polyprotein processing strategy for gene expression and does not undergo splicing [23]. This indicates that eIF4A3 can regulate m^6^A modification not only on endogenous spliced mRNAs near junction regions but also on exogenous viral RNAs. However, whether eIF4A3 modulates m^6^A methylation on endogenous mRNAs away from splice junctions remains an open question worthy of further investigation. Additionally, our GO and KEGG analyses of EHMGs associated with viral infection—including those involved in defense response, hypersensitive response, RNA degradation, and mRNA surveillance—are affected, suggesting their potential contribution to SCMV infection. Moreover, EHMGs linked to nuclear homeostasis may indirectly influence the progression of SCMV infection by modulating gene expression. However, the altered expression of genes within these pathways does not necessarily indicate enhanced or impaired pathway functionality; further mechanistic studies are needed to clarify their exact roles during infection.

Our research expands the understanding of the molecular mechanism of DEAD-box RNA helicases in viral infections. As the largest family of RNA helicases, DEAD-box helicases function in nearly all RNA metabolic processes [77–79]. Several studies suggest that specific DEAD-box helicases function as pro-viral factors recruited into VRCs [80, 81]. For instance, Ded1p is a component of the tomato bushy stunt virus (TBSV) VRC, binds to the 3′-end of viral minus-stranded RNA to enhance plus-strand synthesis by the viral replicase. *Arabidopsis* eIF4A3-like helicase AtRH2, which enhances TBSV replication by binding to (−) RNA, destabilizing replication intermediates, and enhancing viral replicase cycling for multiple rounds of (+) strand synthesis. Despite these advances, the recruitment mechanisms of these helicases into VRCs remain largely unknown. In this study, we demonstrate that ZmeIF4A3 is specifically recruited into VRCs via direct interaction with the NIa-Pro. Truncation of NIa-Pro (residues 1-194) abolishes this interaction, preventing ZmeIF4A3 localization to 6K2-mCherry induced VRCs. Furthermore, we demonstrated that ZmeIF4A3 enhances SCMV replication via a mechanism different from that of AtRH2. By binding SCMV genomic RNA, ZmeIF4A3 prevents ZmMTA-mediated m^6^A deposition, thereby shielding viral RNA from ZmECT23–ZmCCR4–NOT-dependent decay and simultaneously enhancing replication. Strikingly, a single-amino-acid variant, ZmeIF4A3-like, exhibits similar pro-viral activity and also interacts with NIa-Pro, suggesting its potential recruitment into VRCs. This enables the cumulative suppression of the m^6^A-mediated antiviral response without severely disrupting the normal cellular functions of ZmeIF4A3. Given the functional parallels between AtRH2 and ZmeIF4A3, ZmeIF4A3 and related eIF4A3-like proteins may enhance viral infection beyond m^6^A suppression, possibly in a manner analogous to that of AtRH2.

Moreover, the proviral role of eIF4A3 is not limited to SCMV, as evidenced by its functional conservation in PVY and TuMV, two other potyviruses known to have m^6^A modifications. This suggests that the recruitment of eIF4A3 to suppress m^6^A modification might be a general strategy employed by potyviruses. Given the functional redundancy of ZmeIF4A3 and ZmeIF4A3-like in SCMV infection, editing both using CRISPR–Cas9 to disrupt their interaction with NIa-Pro and enhance m^6^A modification could restore the m^6^A-mediated antiviral pathway [82]. Notably, careful evaluation of potential trade-offs is crucial, as ZmeIF4A3 also regulates endogenous mRNA methylation and stability. These findings redefine eIF4A3 as dual-function arbiters in host-pathogen conflict, bridging RNA modification and antiviral immunity.

In summary, we describe the first plant host factor capable of suppressing m^6^A modification of viral genomic RNA and elucidate its underlying mechanism. SCMV NIa-Pro recruits ZmeIF4A3 into VRCs, where ZmeIF4A3 sterically hinders ZmMTA-mediated m^6^A deposition at position A^6556^ on viral genomic RNA. This suppression shields SCMV genomic RNA from recognition by the ZmECT23-ZmCCR4-NOT antiviral complex, which promotes m^6^A-dependent RNA decay. Remarkably, this eIF4A3-mediated suppression of m^6^A is conserved across potyviral infections. Our work advances mechanistic insights into RNA modification-driven plant antiviral immunity and identifies tractable targets for enhancing crop resistance to potyviral infections, thereby opening new avenues for agricultural biotechnology.

## Supplementary Material

gkaf1432_Supplemental_Files

## Data Availability

The raw sequence data reported in this paper have been deposited in the Genome Sequence Archive in National Genomics Data Center, China National Center for Bioinformation/Beijing Institute of Genomics, Chinese Academy of Sciences (GSA: CRA022276; CRA022277; CRA029175), and are publicly accessible at https://ngdc.cncb.ac.cn/gsa. The crystal structure of hseIF4A3 was retrieved from the RCSB Protein Data Bank (RCSB PDB) (https://rcsb.org/) under accession number 2HYI. The predicted 3D structure of ZmeIF4A3 has been deposited in the AlphaFold Protein Structure Database (https://alphafold.ebi.ac.uk/) under accession number A0A1D6HQ89. Potential m^6^A sites were predicted using SRAMP (http://www.cuilab.cn/m6asiteapp/old). Gene sequences and amino acid sequences used in this study were collected from the Sol Genomics Network (https://solgenomics.net/) and the MaizeGDB website (https://maizegdb.org/). Amino acid multiple sequence alignments were visualized using ESPript 3 (https://espript.ibcp.fr/ESPript/ESPript/). Source data are provided with this paper.
