# Supplementary material for: Potyviruses recruit host eIF4A3 to block m6A-mediated RNA decay by steric hindrance of viral RNA methylation in plants
Source: Nucleic Acids Res. 2026 Jan 8;54(1):gkaf1432. doi: 10.1093/nar/gkaf1432 (PMC12781882; doi:10.1093/nar/gkaf1432)
Supplement: gkaf1432_Supplemental_Files [file gkaf1432_supplemental_files.zip › 20251013 Clean Supplementary Figures 1-17 and Supplementary Tables 1-2.pdf]

**Supplementary Data**

# Potyviruses recruit host eIF4A3 to block $m^6A$-mediated RNA decay by steric hindrance of viral RNA methylation in plants

Authors

Dezhi Peng[1], Laihua Dong[1], Pei Wang[1], Lianyi Zang[1], Jinhao Xie[1], Hao Wang[1], Xiangdong Li[2], Zaifeng Fan[1], Tao Zhou[1], Kaitong Du[1*]

Affiliations

[1]State Key Laboratory of Maize Bio-breeding and Department of Plant Pathology, China Agricultural University, Beijing 100193, China

[2]College of Plant Protection, Shandong Agricultural University, Taian 271018, Shandong, China

*Author for correspondence: Kaitong Du, nongdadukaitong@cau.edu.cn

Supplementary Figures S1-S17

Supplementary Tables S1-S2

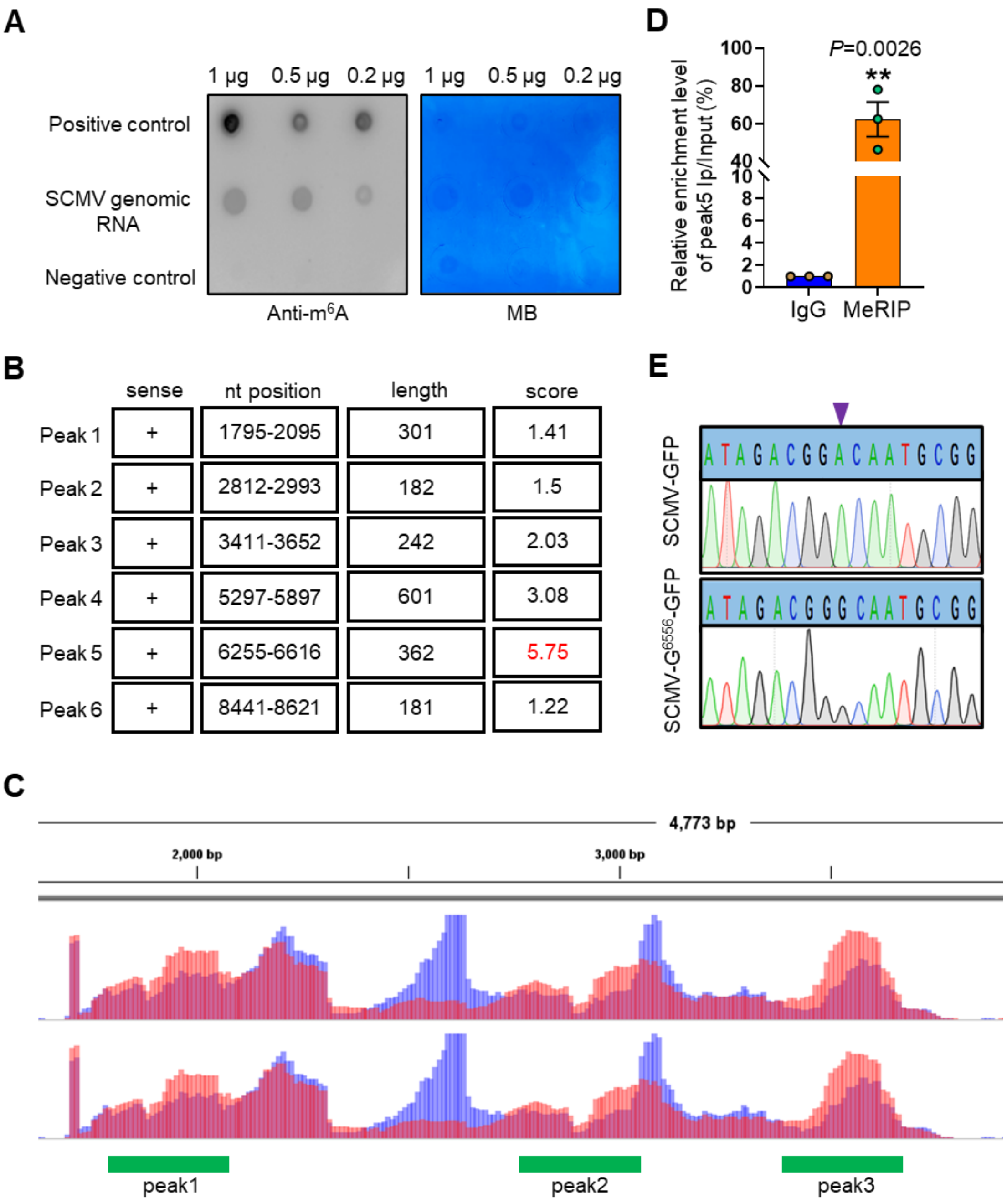


**Figure S1. m⁶A modifications occur at peak5 (nt 6255 to 6616) of SCMV genomic RNA.** (A) Dot blot analysis of SCMV genomic RNA extracted from particles suggested that the SCMV genomic RNA was modified by m⁶A. Total RNA extracted from B73 maize was used as the positive control, while unmodified *in vitro*-transcribed RNAs (IVT RNAs) served as the negative control. MB staining was conducted to show equal loading of total RNA. (B) Nucleotide localization and enrichment of the m⁶A peaks identified within the genomic RNA of SCMV were determined through MeRIP-seq. “score,” a statistic that quantifies the significance of MeRIP IP enrichment relative to input; higher values indicate stronger evidence for m⁶A in that region. Peak5, demonstrating the highest score, is represented in red. (C) Zoomed views of peak1–3. Enlarged genome-browser snapshots of the three m⁶A-enriched regions. Red bars indicate m⁶A-IP peaks; blue bars show matched input. Orange bars highlight the differential signal (IP minus input). Peak locations are

marked with green lines. (D) MeRIP-qPCR assays identified $m^6A$ modifications at nucleotides 6255 to 6616 of the SCMV genomic RNA. Total RNA extracted from SCMV-infected plants was incubated with either anti-$m^6A$ antibodies or IgG (as a negative control) for IP. The $m^6A$-methylated RNAs from IP were amplified by qRT-PCR. Values are means ± SE (two tailed Student's *t*-test, n = 3 independent experiments, $^{**}P < 0.01$). (E) DNA sequencing chromatograms confirmed the presence of an "A-to-G" mutation in the *NIa-Pro* sequence of maize plants infected with SCMV-$G^{6556}$-GFP. The mutation site marked by a purple arrow.

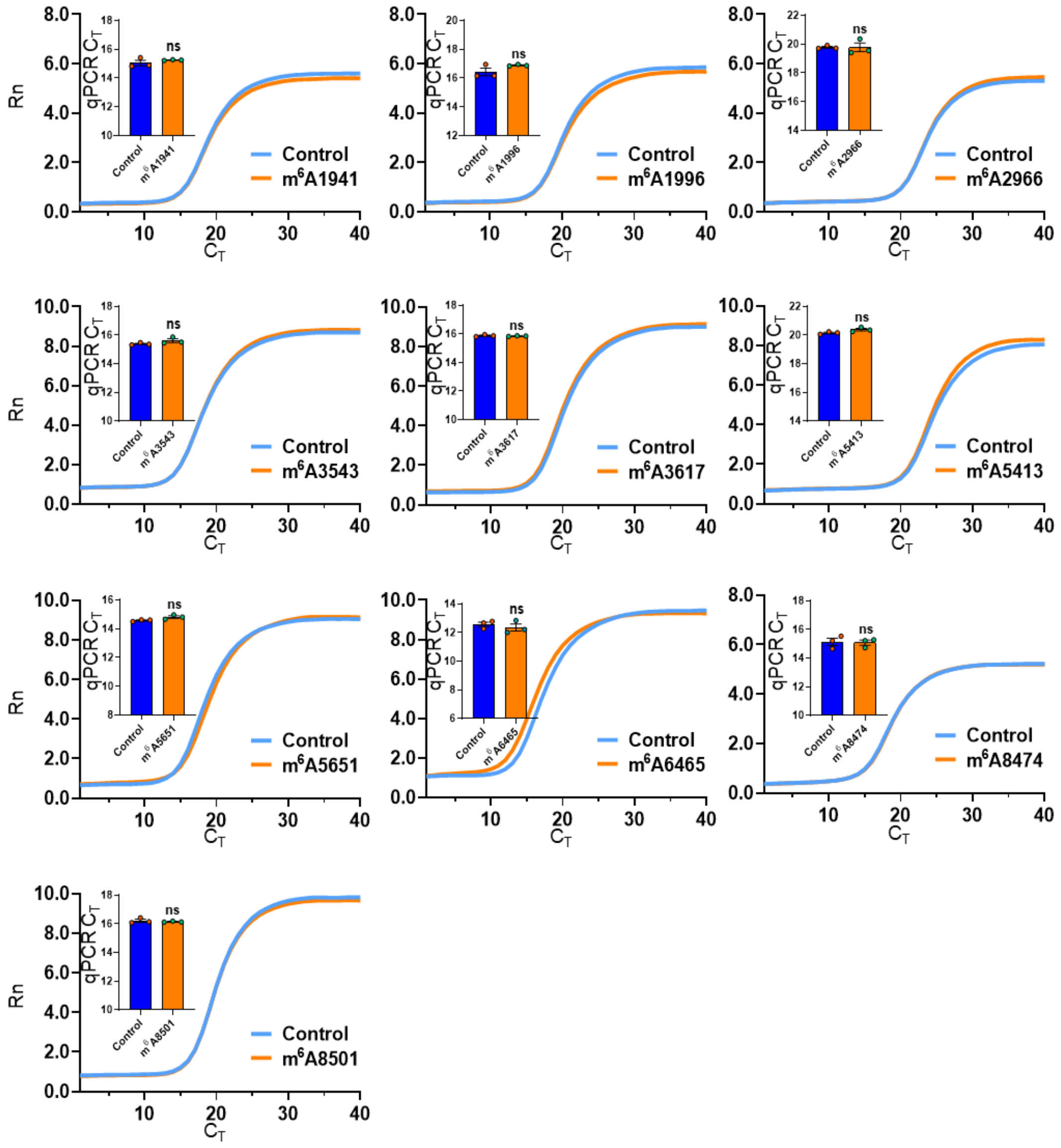


**Figure S2. Detection of potential m$^6$A sites via SELECT-qPCR analysis.** Fluorescence amplification curves and a bar plot of the qPCR CT value indicated that these potential sites were not modified by m$^6$A. Values are expressed as means ± SE (two tailed Student's *t*-test, n = 3 independent experiments). "ns" indicates no significance difference.

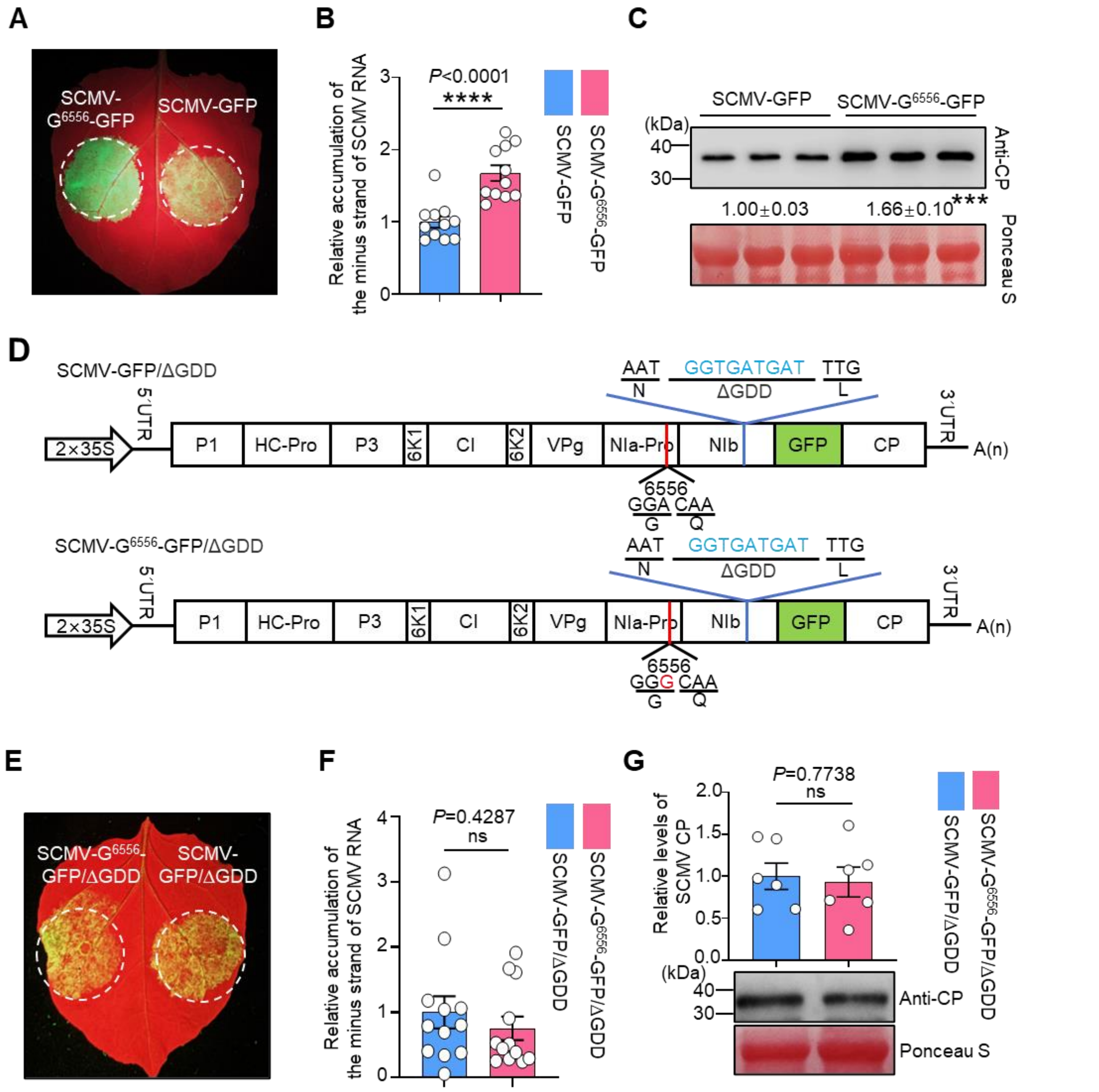


**Figure S3. m$^6$A modifications at A$^{6556}$ inhibits SCMV RNA replication.** (A) SCMV-G$^{6556}$-GFP mutant virus caused stronger GFP fluorescence in *N. benthamiana* leaves compared with SCMV-GFP. Photographs were taken at 5 days post-infiltration. (B–C) Quantification of SCMV negative-strand RNA and CP levels, respectively. (D) Schematic representation of the replication-deficient SCMV-GFP/ΔGDD and SCMV-G$^{6556}$-GFP/ΔGDD mutants. The conserved GDD motif (residues 352–354) within the RNA-dependent RNA polymerase (NIb) was deleted in both infectious clones; nucleotides corresponding to GDD are highlighted in blue. (E) GFP fluorescence in *N. benthamiana* leaves did not differ between SCMV-G$^{6556}$-GFP/ΔGDD and SCMV-GFP/ΔGDD mutant virus. (F–G) Quantification of SCMV negative-strand RNA, and CP accumulation from ΔGDD mutants. Data represent mean ± SE; in (B, F) n = 11 or 12 infiltrated leaf patches from 3 independent experiments; (C, G) n = 3 or 6 infiltrated leaf patches. Statistical significance was determined by two-tailed Student's *t*-test: ****P* < 0.001, *****P* < 0.0001, ns, not significant.

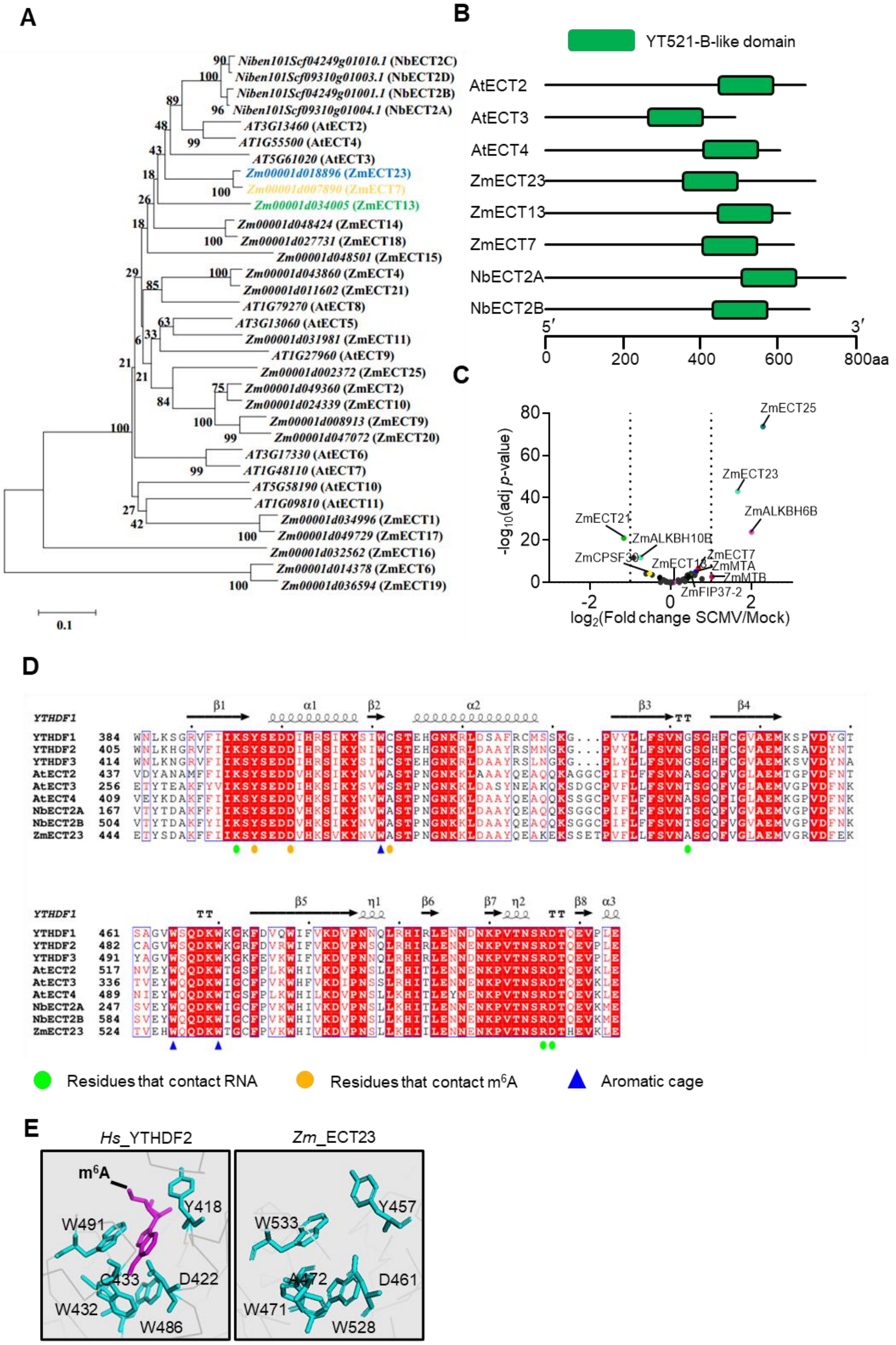

**Figure S4. ZmECT23 is a potential m⁶A reader protein in maize**. (A) Phylogenetic tree showed the relationship between m⁶A readers from *Arabidopsis thaliana* (At) and *Nicotiana benthamiana* (Nb). Sequence alignments and tree construction were performed using MEGA6, with bootstrap

values from 1000 replicates and displayed on internal nodes. (B) Domain compositions of ECT proteins, all of which contained a conserved YT521-B-like domain. (C) A filtered volcano plot showed the expression of genes involved in the $m^6A$ pathway in response to SCMV infection. (D) Multiple sequence alignment of maize ZmECT23 with $m^6A$ readers from *A. thaliana* (At), *N. benthamiana* (Nb), and *Homo sapiens* (Hs) is shown. The secondary structural elements of HsYTHDF1 (PDB: 4RCJ) are depicted at the top. (E) Aromatic cage structure responsible for $m^6A$ binding, along with the hydrogen bond interactions with $m^6A$, is conserved between HsYTHDF2 (PDB: 4RDN) and ZmECT23.

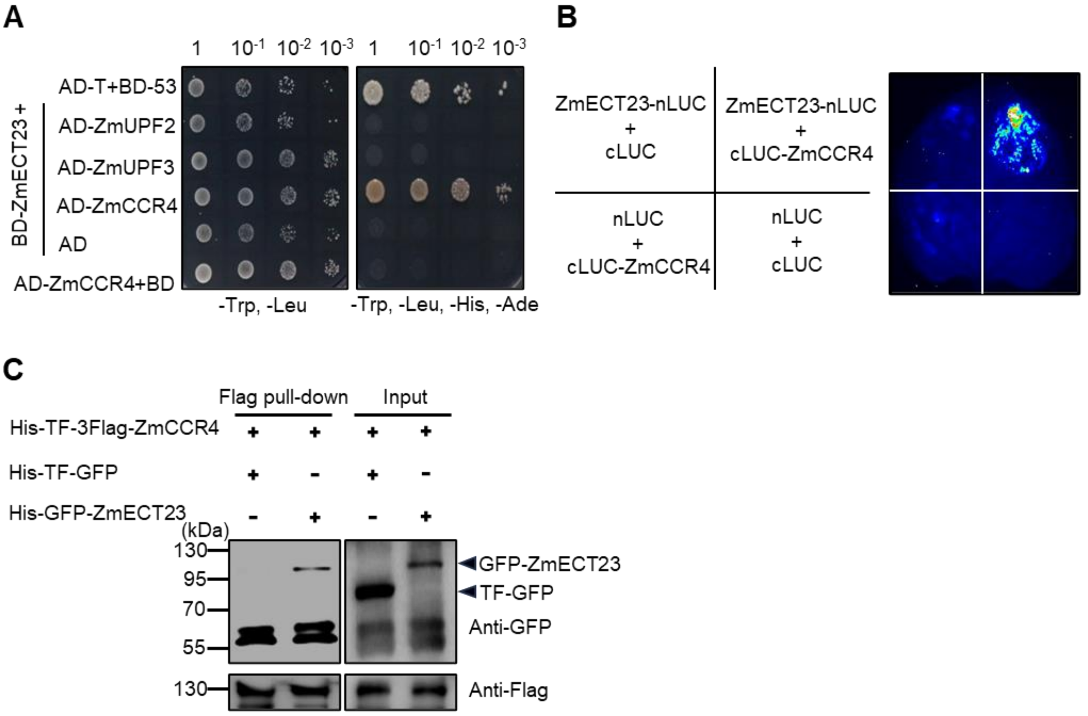


**Figure S5. Interactions between ZmECT23 and ZmCCR4 *in vivo* and *in vitro***. (A) Yeast two-hybrid (Y2H) assay showed the interaction between ZmECT23 and ZmCCR4. Positive control, yeast cells co-transformed with plasmids AD- T7 and BD- 53; negative control, AD- ZmCCR4 and empty BD, as well as AD and BD- ZmECT23. (B) Firefly luciferase complementation imaging (LCI) analysis of the ZmECT23 and ZmCCR4 interaction in *N. benthamiana* leaf cells. *Agrobacterium* strain GV3101 harbouring the LUC constructs were co-infiltrated into *N. benthamiana* leaves. Images of infiltrated leaves of *N. benthamiana* were taken three days later. (C) Physical interaction between ZmECT23 and ZmCCR4 was established using pull-down assays. Black arrowheads indicate bands of GFP-ZmECT23 or TF-GFP. Molecular weight markers are shown on the left of each panel.

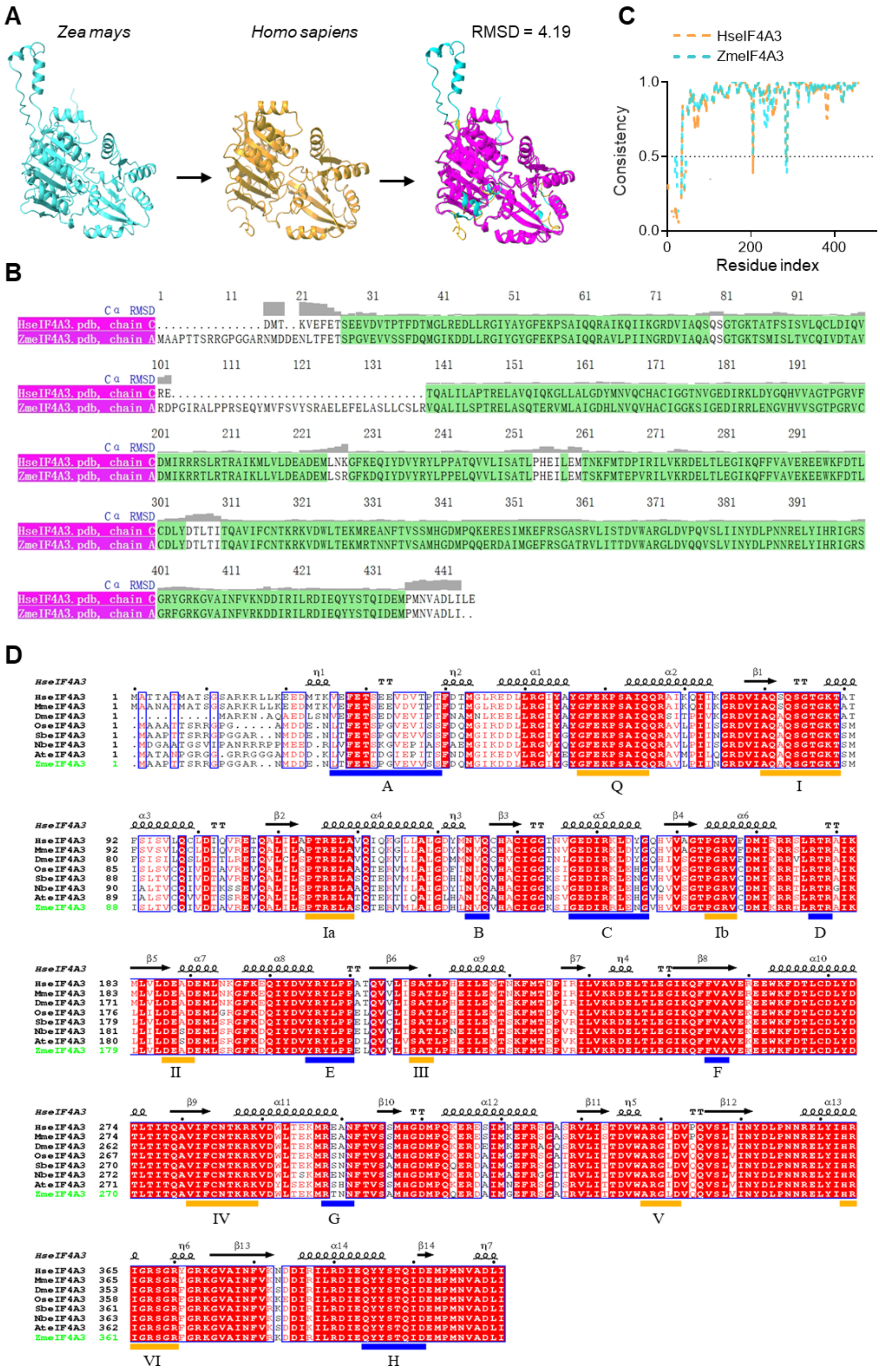


**Figure S6. The three-dimensional (3D) structures and the multiple sequence alignments of ZmeIF4A3 and HseIF4A3 proteins exhibit substantial similarity.** (A–B) 3D structures of ZmeIF4A3 (A0A1D6HQ89), predicted by AlphaFold2, is depicted in light blue, whereas the 3D structure of HseIF4A3 (PDB: 2HYI) is represented in pale yellow. Magenta regions denote areas of structural similarity between the two proteins, characterized by a relatively low root mean square deviation (RMSD). The RMSD values, calculated between aligned pairs of the backbone C-alpha

atoms in superposed structures, are displayed above the alignment in angstroms (Å). An RMSD value below 10 Å is generally considered to indicate structural similarity. (C) Scatter plot showed the consistency of amino acid residues between HseIF4A3 and ZmeIF4A3. (D) Multiple sequence alignment compared eIF4A3 across maize and other species, including *H. sapiens* (Hs), *Mus musculus* (Mm), *Drosophila melanogaster* (Dm), *Oryza sativa* (Os), *Sorghum bicolor* (Sb), *N. benthamiana* (Nb), *A. thaliana* (At). The secondary structural elements of HseIF4A3 (PDB: 2HYI) were illustrated at the top, with $3_{10}$-helices, α-helices and π-helices represented by small, medium and large squiggles, respectively. β-strands were depicted as arrows, and strict β-turns as "TT" letters. Regions highlighted in red indicate strict identity, red characters denote similar amino acids, blue frames indicate high similarity across groups, and black letters represent amino acids with low similarity. Positions of eIF4A family consensus motifs (Q–VI) and eIF4A3-specific motifs (A–H) are indicated by orange and blue bars, respectively.

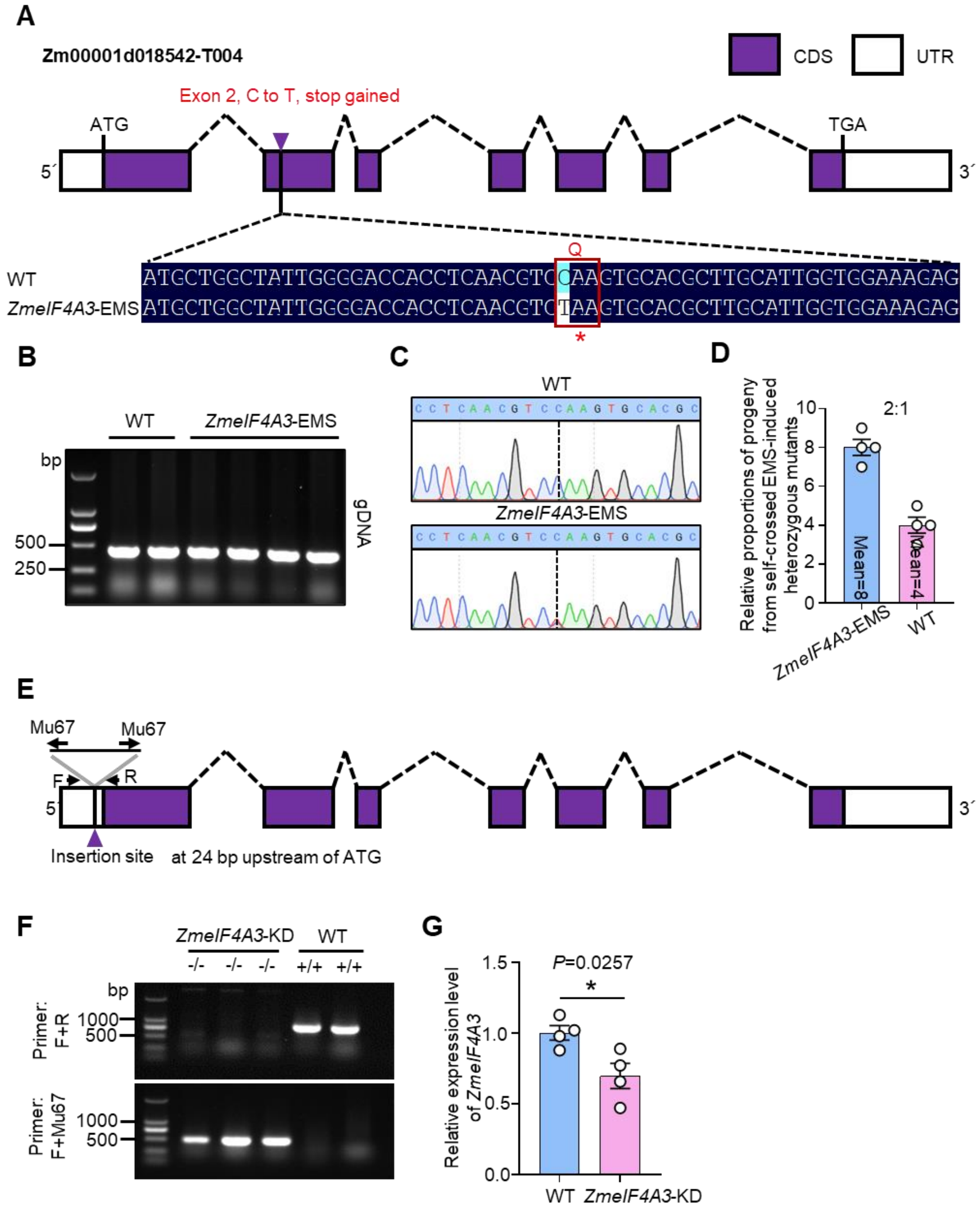


**Figure S7. Identification of EMS-induced and *Mutator*-insertion *ZmeIF4A3* mutations.** (A) Illustration of a mutation in *ZmeIF4A3* transcript T004. *ZmeIF4A3*-EMS line carries a single C to T substitution, resulting in a premature stop codon in *ZmeIF4A3* transcripts. Coding regions are represented by purple rectangles, introns by interconnecting black lines, and untranslated regions by black boxes. (B) Genomic DNA extracted from WT (B73) leaves and the *ZmeIF4A3*-EMS line leaves was subjected to PCR followed by sequencing analysis. (C) DNA sequencing chromatograms confirmed the presence of heterozygous mutation in the *ZmeIF4A3*-EMS line. (D) Self-crossing of the heterozygous *ZmeIF4A3*-EMS individuals, resulting in a segregation ratio of 2:1 for heterozygous to WT plants. (E) Illustration of a mutator insertion within 5′ untranslated regions (5′

UTR) of the *ZmeIF4A3* transcript T004, specifically at the 24 bp upstream of the ATG start codon. (F) PCR-based genotyping confirmed the homozygous *ZmeIF4A3*-KD mutant. WT refers to wild-type. F+R/F+Mu67: '+/+' indicates no insertion, and '-/-' indicates homozygous insertion. (G) *ZmeIF4A3* expression levels were significantly reduced in the *ZmeIF4A3*-KD mutant plants using qRT-PCR analysis. Data are expressed as the means ± SE (two-tailed Student's *t*-test, n = 4 plants, $^*P < 0.05$).

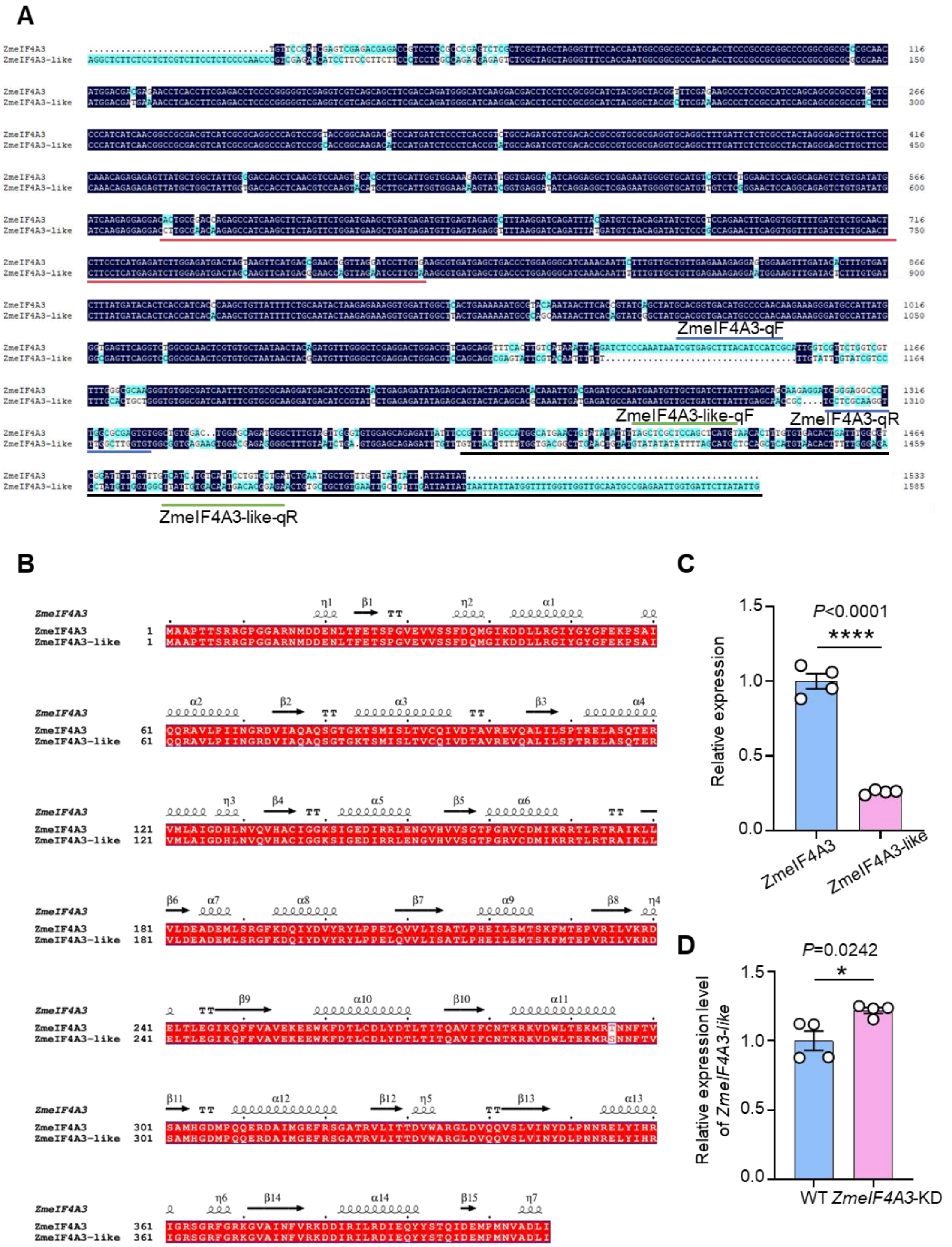


**Figure S8. Alignment of cDNA and protein sequences of *ZmeIF4A3* and *ZmeIF4A3-like*.** (A) The cDNA sequences of *ZmeIF4A3* and *ZmeIF4A3-like* were aligned using DNAMAN software. The red line indicates the 200 bp region, which was chosen to simultaneously silence both *ZmeIF4A3* and *ZmeIF4A3-like*. The black lines indicate partial fragments in 3′ untranslated regions (3′ UTR) of *ZmeIF4A3-like*, which was used for specific silencing of *ZmeIF4A3-like*. The blue and green segments represent the specific primers for *ZmeIF4A3* and *ZmeIF4A3-like*, respectively, designed to evaluate their expression levels. (B) Multiple sequence alignment using ESPript3 revealed highly conserved amino acid sequences in ZmeIF4A3 and ZmeIF4A3-like. The predicted

secondary structural elements of ZmeIF4A3 (A0A1D6HQ89), generated by AlphaFold2, are shown at the top. (C) qRT-PCR analysis revealed that the expression level of *ZmeIF4A3-like* in maize leaves was significantly lower than that of *ZmeIF4A3*. (D) Expression levels of *ZmeIF4A3-like* were slightly increased in *ZmeIF4A3*-KD plants, as determined by qRT-PCR analysis. Data are expressed as the means ± SE (n = 4 plants). Statistical differences in (C) and (D) were determined using the two-tailed Student's *t*-test (*$P < 0.05$).

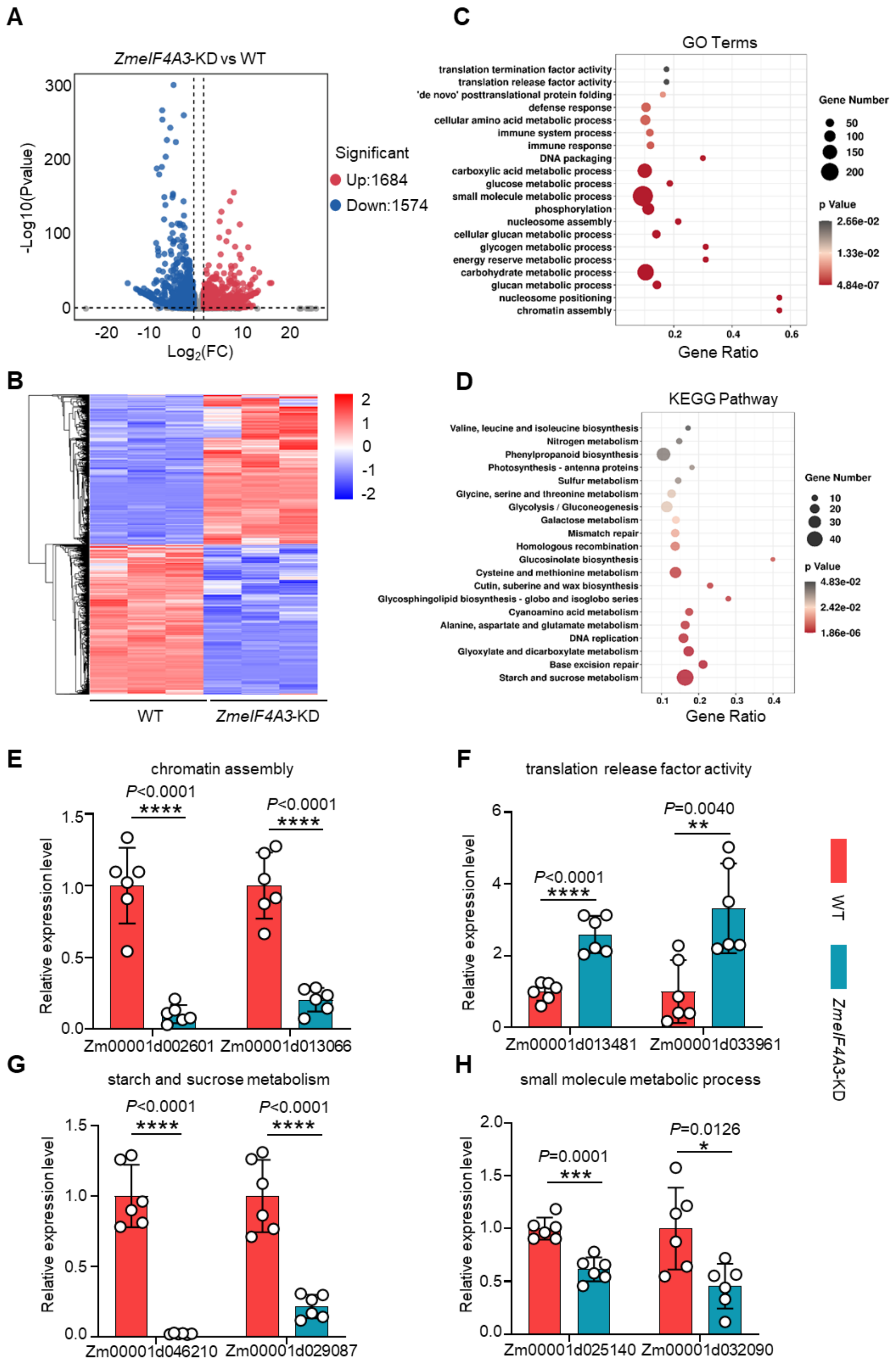


**Figure S9. Transcriptome analysis of WT and *ZmeIF4A3*-KD maize seedlings.** (A) Volcano plots representing the fold change in the expression of the DEGs in the WT versus *ZmeIF4A3*-KD comparisons ($p < 0.05$, fold change > 2.0). (B) Hierarchical clustered heatmap of 3,258 DEGs (1,684

upregulated genes and 1,574 downregulated genes). (C) GO analysis showing 20 representative enrichment terms of all the DEGs. The significance of the GO terms was using adjusted $p < 0.05$ (Fisher's exact test). (D) KEGG pathway analysis of all the DEGs in the *ZmeIF4A3*-KD plants. Top 20 significantly enriched ($p < 0.05$) categories in KEGG are presented. (E–H) qRT-PCR validation of eight DEGs from the RNA-seq data. Selected genes are involved in chromatin assembly, translation release factor activity, starch and sucrose metabolism, and small molecule metabolic process. *Ubiquitin* was used as an internal control. Values are means with SE; n = 6 plants from 2 independent experiments (**P* < 0.05; ***P* < 0.01; ****P* < 0.001; *****P* < 0.0001; two-tailed Student's *t* test).

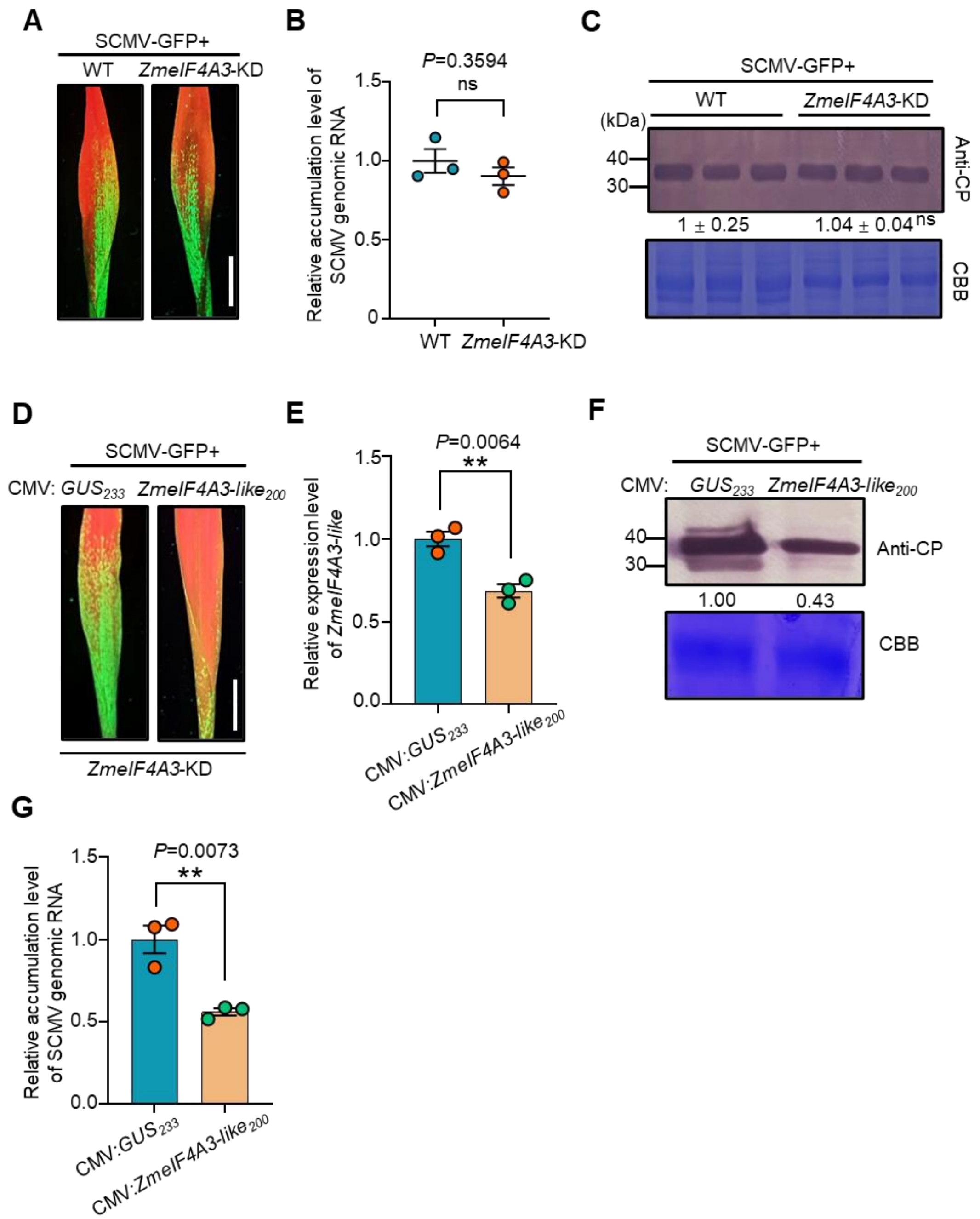


**Figure S10. Suppression of SCMV-GFP infection in *ZmeIF4A3*-KD mutant plants upon *ZmeIF4A3-like* knockdown through CMV-based gene silencing.** (A) Upper leaves of SCMV-GFP-infected *ZmeIF4A3*-KD plants showed similar GFP fluorescence compared to WT plants at 5 dpi. Scale bar, 2 cm. (B–C) Accumulation levels of viral genomic RNA and CP in the *ZmeIF4A3*-KD plants showed a modest reduction compared to WT plants, as assessed by qRT-PCR and immunoblotting analysis. (D) Milder GFP fluorescence were observed in the 1 SL of SCMV-GFP-infected plants compared to control plants at 7 dpi. Scale bar, 2 cm. (E) Relative expression levels of *ZmeIF4A3-like* were significantly reduced in *ZmeIF4A3*-KD plants compare to CMV:*GUS*-infected control plants (n = 3). (F) Immunoblotting analysis of SCMV CP accumulation in *ZmeIF4A3-like*-silenced and control plants (n = 3). Relative intensities of the CP bands were quantified. CBB-stained served as the loading control. (G) Quantification of SCMV genomic RNA

accumulation levels in the 1 SL of *ZmeIF4A3-like*-silenced or control plants using qRT-PCR. Data are expressed as the means ± SE. Statistical differences in (B–C), (E), and (G) were determined using the two-tailed Student's *t*-test (**$P$< 0.05; *$P$< 0.05; ns, no significant difference).

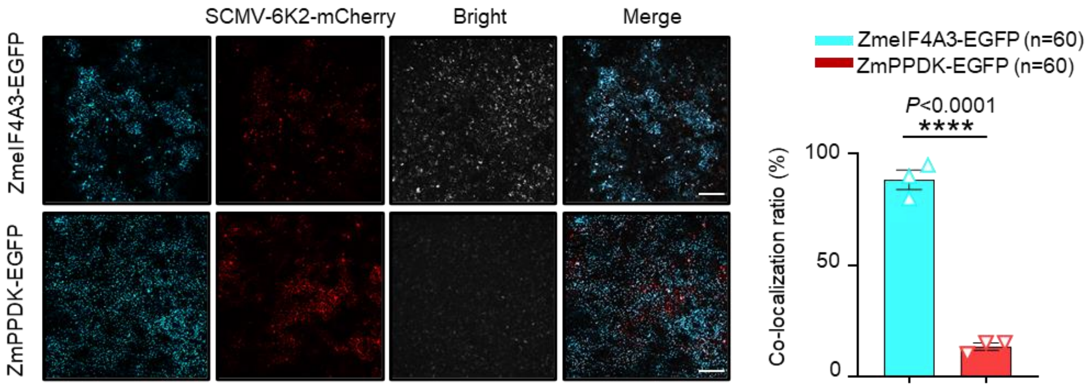


**Figure S11. ZmeIF4A3-EGFP co-localizes with SCMV-6K2-mCherry.** Co-localization of ZmeIF4A3-GFP and ZmPPDK-GFP (negative control) with SCMV-6K2-mCherry in the leaf cells of *N. benthamiana* at 5 days post-infiltration. Scar bars, 100 μm. Quantification (n = 60 cells from three independent images) showed significantly co-localization between ZmeIF4A3-EGFP and SCMV-6K2-mCherry. Data are presented as mean ± SE. Statistical differences was determined using the two-tailed Student's *t*-test (*****P* < 0.0001).

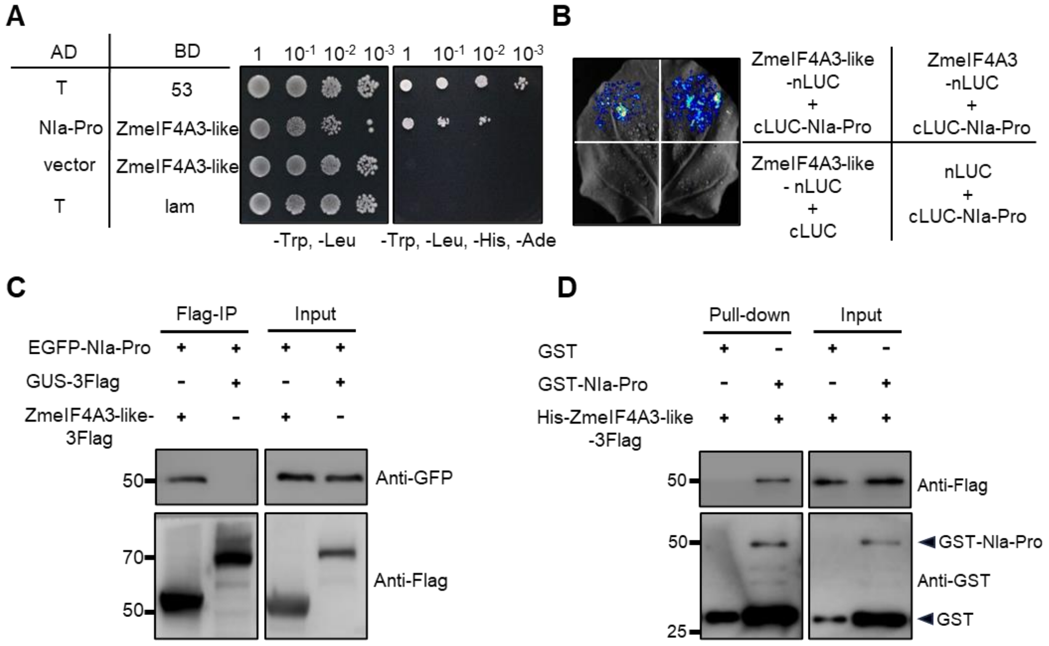


**Figure S12. ZmeIF4A3-like interacted with NIa-Pro *in vivo* and *in vitro*.** (A) Y2H assays showed the interaction between ZmeIF4A3-like and NIa-Pro. (B) LCI assay confirmed the ZmeIF4A3-like–NIa-Pro interaction. (C) Co-immunoprecipitation (Co-IP) analysis showed the interactions between NIa-Pro with ZmeIF4A3-like. (D) GST pull-down assays confirmed the direct interaction between NIa-Pro and ZmeIF4A3-like. Black arrowheads indicate GST-NIa-Pro or GST.

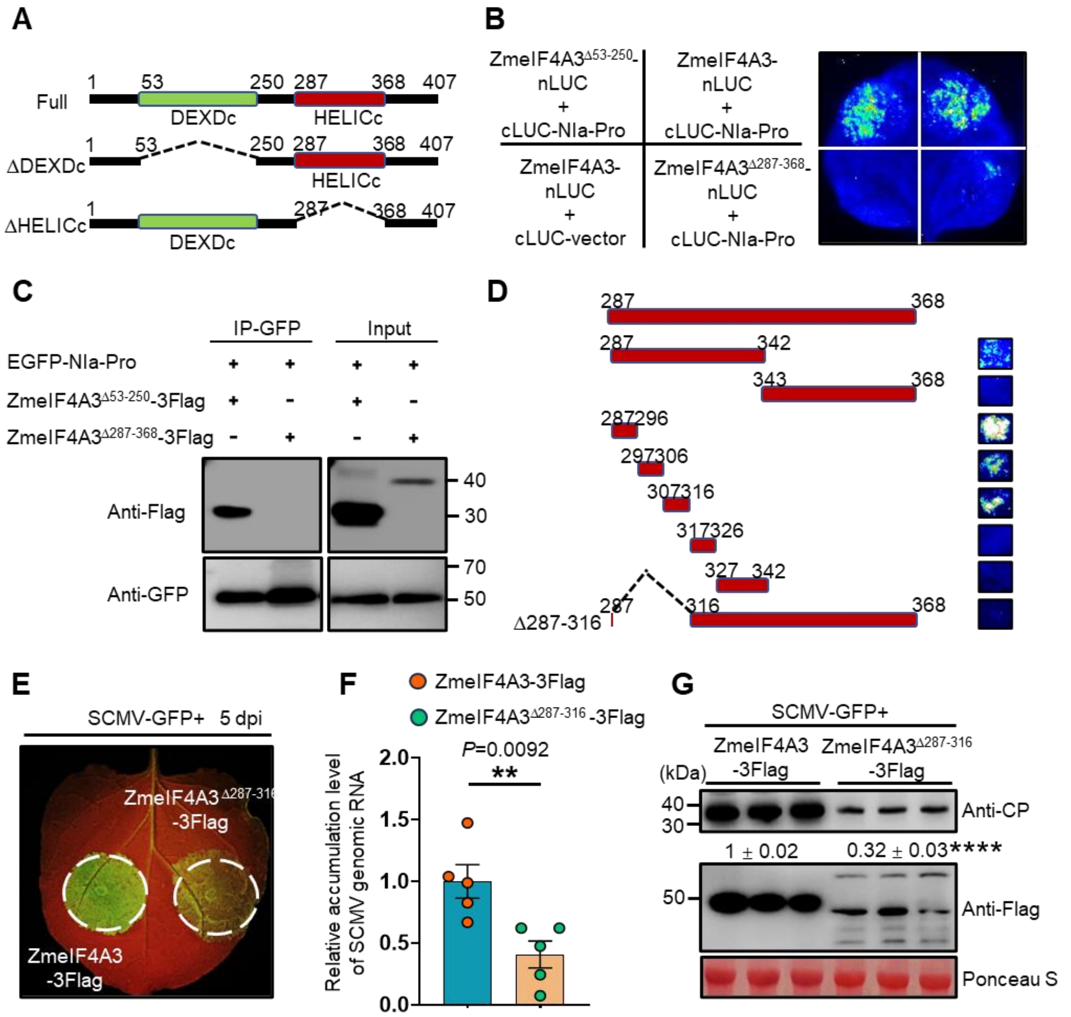


**Figure S13. ZmeIF4A3 promotes SCMV infection dependent on its interaction with NIa-Pro.** (A) Schematic diagram of the ZmeIF4A3 protein structure and its truncated mutants. The DEXDc domain is depicted in green box, the HELICc domain in red box, and the regions of deletion are indicated by interconnecting black lines. (B–C) *In vivo,* the 287-368 region of ZmeIF4A3 interacted with NIa-Pro using LCI assays and Co-IP analysis. (D) Interactions of NIa-Pro with ZmeIF4A3$^{287\text{-}342}$, ZmeIF4A3$^{343\text{-}368}$, ZmeIF4A3$^{287\text{-}296}$, ZmeIF4A3$^{297\text{-}306}$, ZmeIF4A3$^{307\text{-}316}$, ZmeIF4A3$^{317\text{-}326}$, ZmeIF4A3$^{327\text{-}342}$ and ZmeIF4A3$^{\Delta 287\text{-}316}$ fragments *in vivo* LCI assay. The 287-316 domain of ZmeIF4A3 interacted with NIa-Pro. (E) Expression of ZmeIF4A3$^{\Delta 287\text{-}316}$ inhibited SCMV replication in *N. benthamiana* leaves. pSCMV-GFP was agroinfiltrated into *N. benthamiana* leaves with ZmeIF4A3-3Flag or ZmeIF4A3$^{\Delta 287\text{-}316}$-3Flag. GFP intensity was monitored at 5 days post-infiltration. (F–G) qRT-PCR and immunoblotting analyses showed that the expression of ZmeIF4A3$^{\Delta 287\text{-}316}$-3Flag led to a significant reduction in the accumulation levels of SCMV genomic RNA and CP, compared to the expression of ZmeIF4A3-3Flag. Data are represented as means ± SE (n = 5 infiltrated leaf patches). Asterisks indicate significant differences calculated using two-tailed Student's *t*-test (****$P$ < 0.0001; **$P$ < 0.01). Relative band intensity of CP was quantified by ImageJ software. The Ponceau S-stained blots were used to show sample loadings.

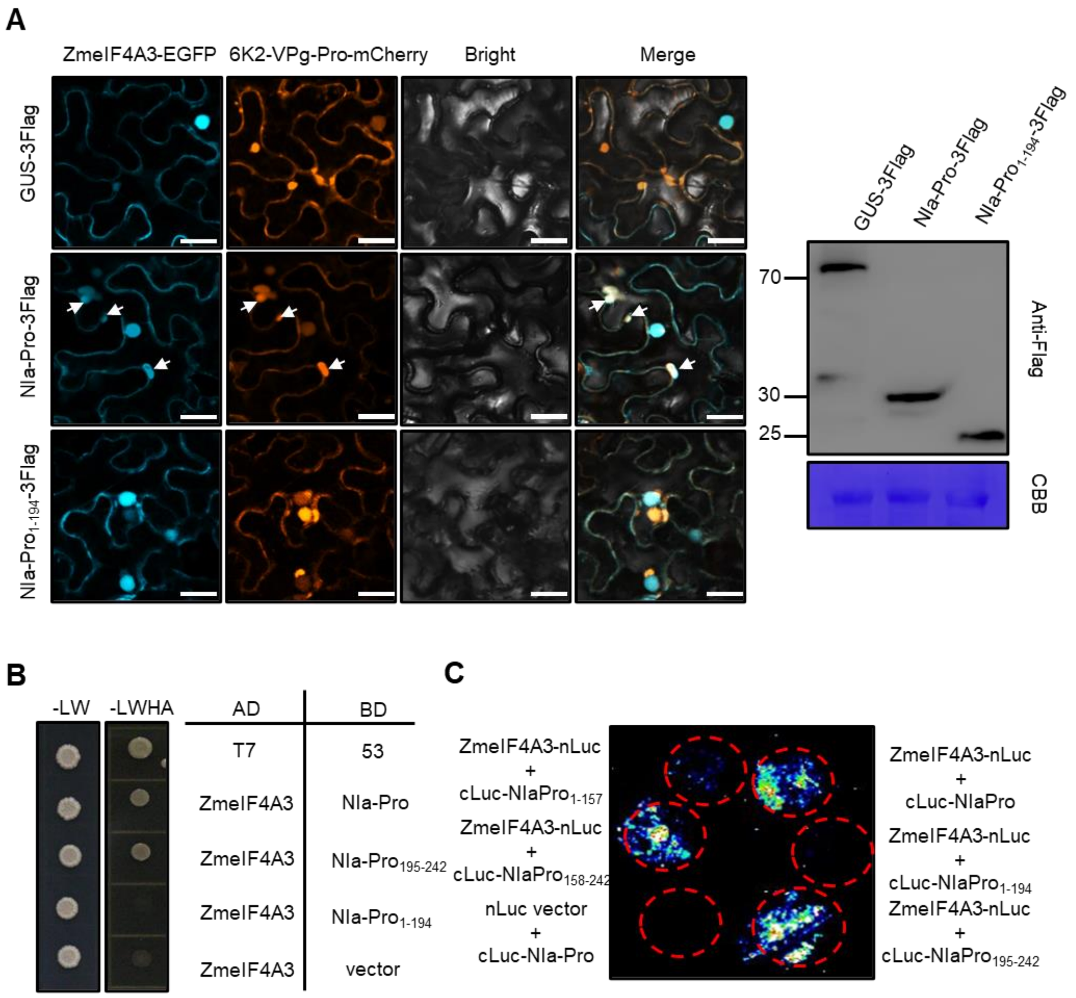


**Figure S14. NIa-Pro recruits ZmeIF4A3 into VRCs via their interaction.** (A) NIa-Pro recruited ZmeIF4A3 into VRCs via its C-terminal residues 195-242. ZmeIF4A3-EGFP and 6K2-VPg-Pro-mCherry were co-expressed with GUS-3Flag, NIa-Pro-3Flag, or NIa-Pro$_{1-194}$-3Flag in *N. benthamiana* leaves via *Agrobacterium* infiltration. 3 days post-infiltration, the infiltrated leaf tissues were selected for confocal microscopy analysis. White arrows in middle panel indicate co-localization of ZmeIF4A3-EGFP and 6K2-VPg-Pro-mCherry. Scale bars, 20 μm. Immunoblotting confirmed the expression of GUS-3Flag, NIa-Pro-3Flag, and NIa-Pro$_{1-194}$-3Flag. The blot was probed by anti-Flag antibody. The CBB-stained RbcL served as the loading control. (B) ZmeIF4A3 interacted with the 195-242 amino acid region of NIa-Pro in yeast. (C) LCI assays showed that ZmeIF4A3 specifically interacted with the C-terminal 195-242 amino acid region of NIa-Pro.

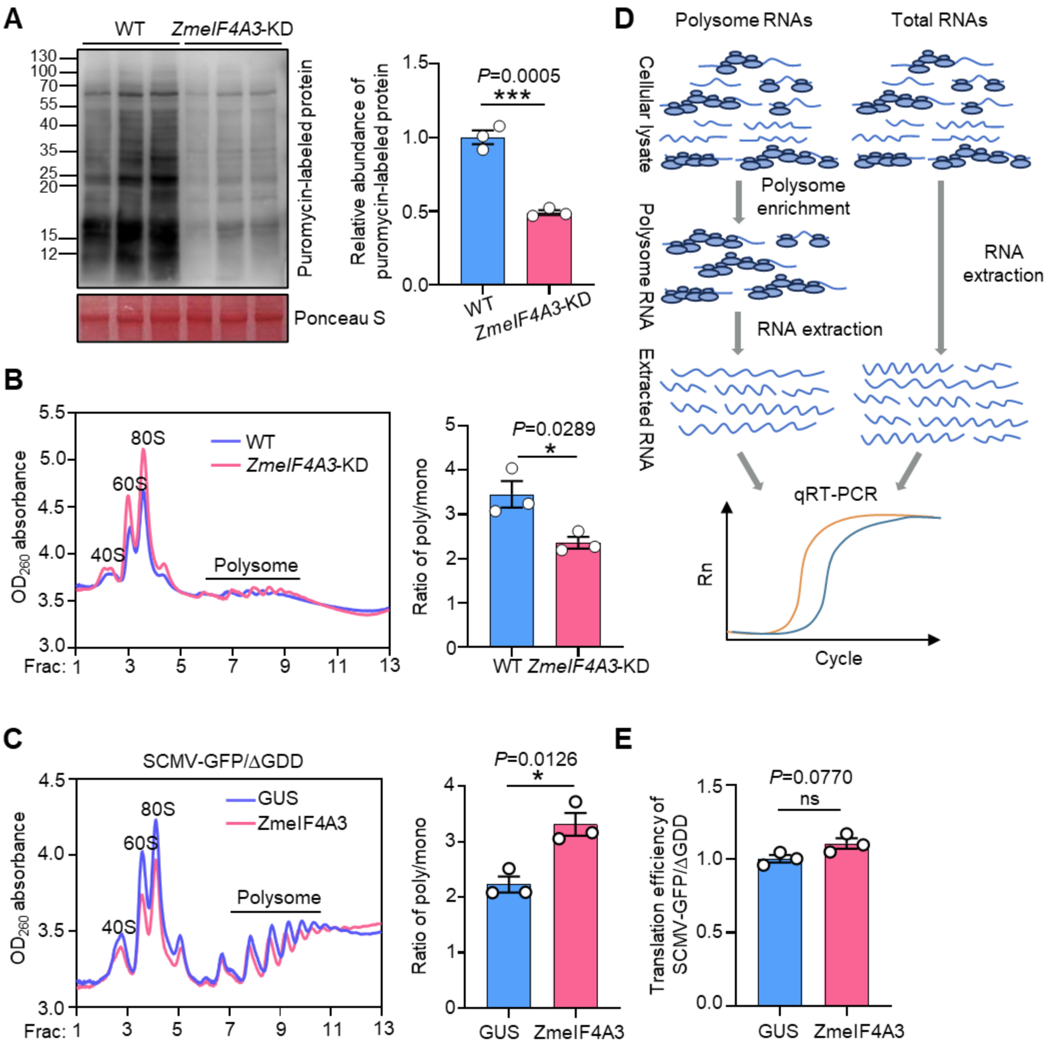


**Figure S15. ZmeIF4A3 does not affect SCMV RNA translation.** (A) Surface Sensing of Translation (SUnSET) assay detecting new synthesized proteins in WT and *ZmeIF4A3*-KD plants, quantification of the relative abundance of puromycin-labeled protein was performed using Ponceau S–stained blots as the loading control. (B) Representative absorbance plot in polysome profiling of WT and *ZmeIF4A3*-KD. Comparison of quantified polysome/monosome (poly/mono) ratios between WT and *ZmeIF4A3*-KD on the right. Data are presented as mean ± SE (two-tailed Student's *t* test, n = 3 independent experiments). (C) Polysome profiling absorbance traces of samples co-expressing GUS with SCMV-GFP/ΔGDD or ZmeIF4A3 with SCMV-GFP/ΔGDD. Quantified polysome/monosome (poly/mono) ratios for GUS versus ZmeIF4A3 conditions are shown on the right. Data represent mean ± SE from three independent experiments. (D) Brief workflow for the analysis of translation efficiency. (E) Translation efficiency of SCMV-GFP/ΔGDD upon express of GUS or ZmeIF4A3. Translation efficiency was expressed as the abundance ratio of mRNA in the polysomal RNA versus the total RNA. Data are presented as mean ± SE (two-tailed Student's *t* test, n = 3 independent experiments).

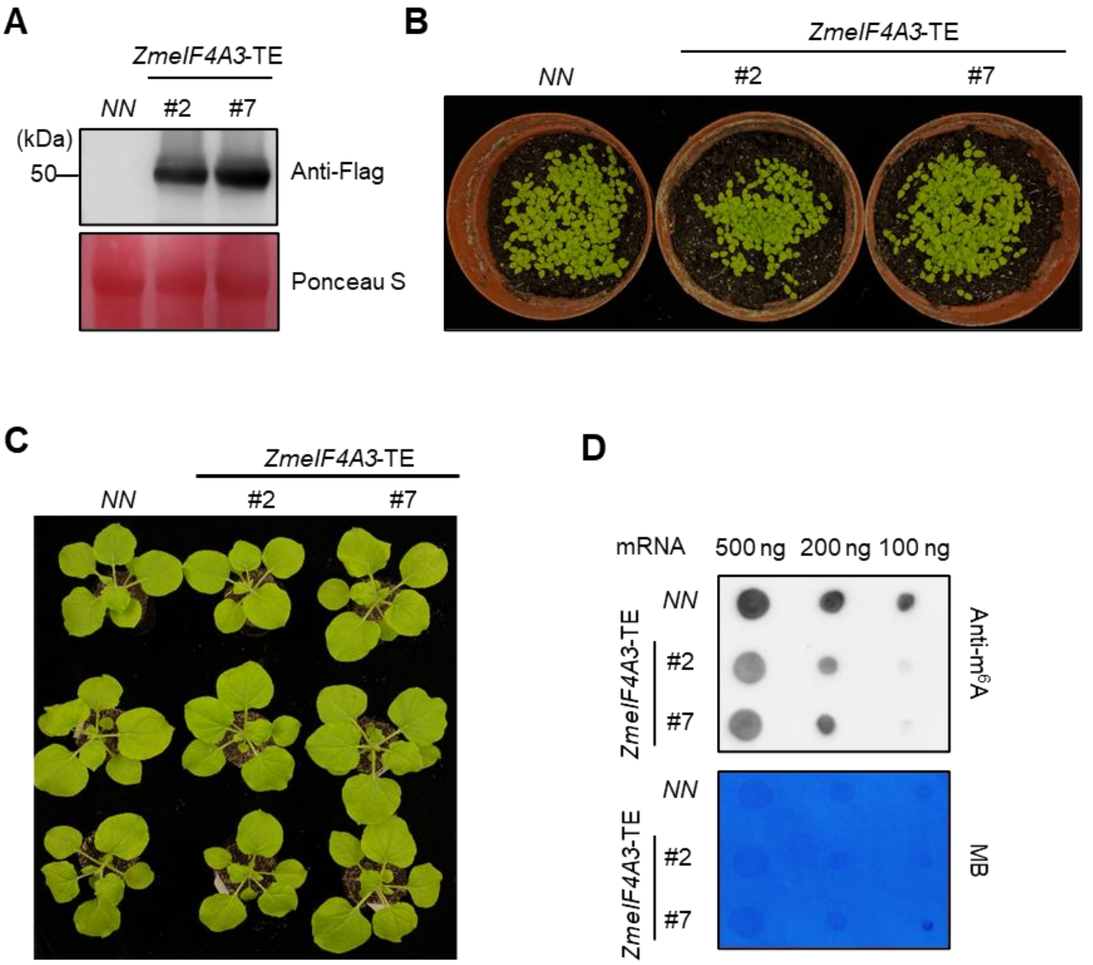


**Figure S16. Generation of *ZmeIF4A3* heterologous expression transgenic *Nicotiana benthamiana* lines.** (A) Immunoblotting confirmed the expression of ZmeIF4A3-3Flag in the *ZmeIF4A3*-TE lines with anti-Flag antibody. The Ponceau S–stained blots indicated sample loadings. (B) The phenotype of *NN* and *ZmeIF4A3* transgenic *N. benthamiana* at 10 days after planting. (C) Phenotype of *NN* and *ZmeIF4A3* transgenic *N. benthamiana* lines at 4 weeks after planting. (D) Dot blot assays showed that the $m^6A$ level was reduced in *ZmeIF4A3* transgenic lines *#2* and *#7* compared to *NN* plants. MB staining showed equal RNA loading.

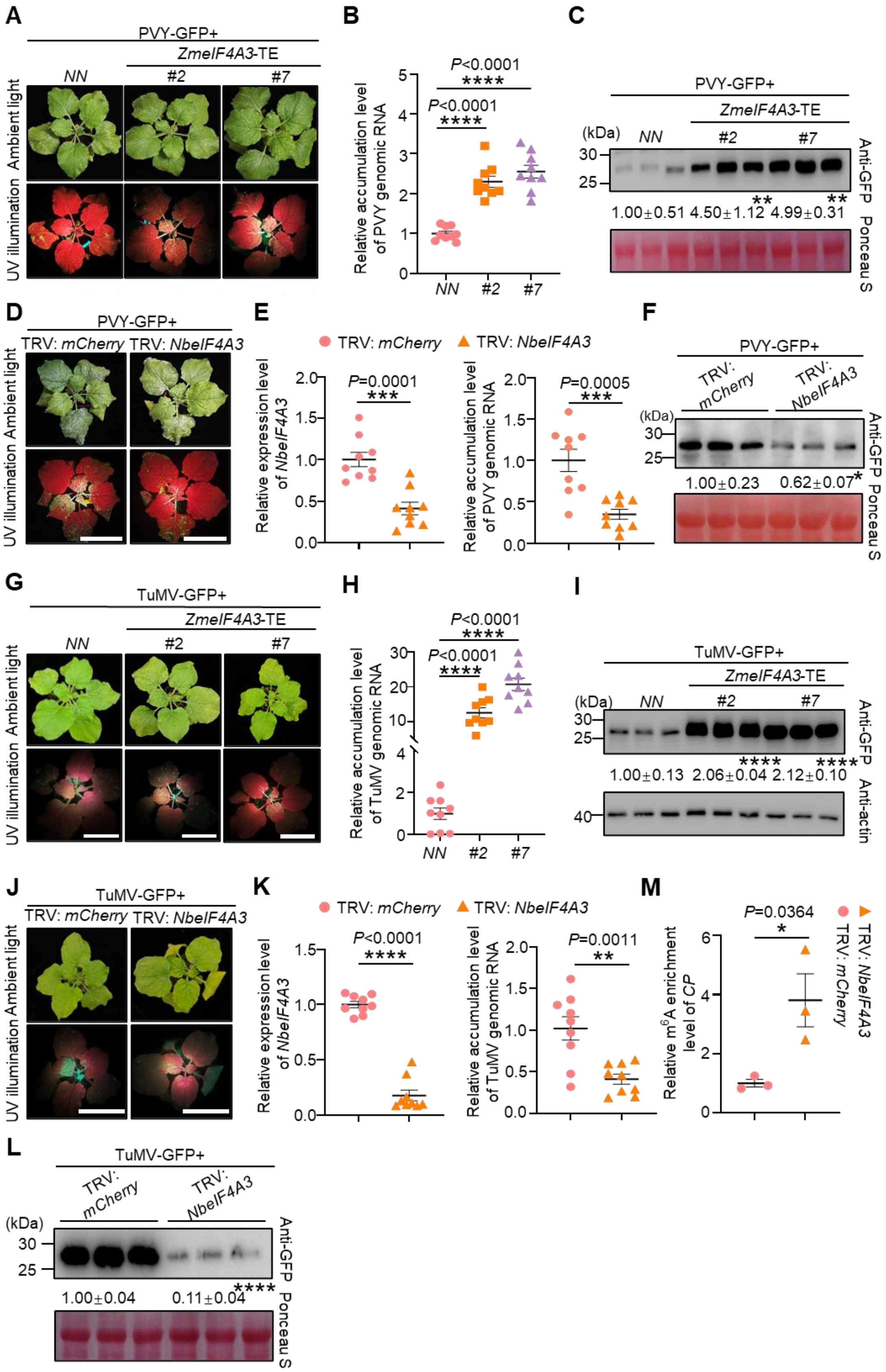


**Figure S17. EIF4A3 facilitates potyviruses infection by suppressing m$^6$A modification.** (A) Stronger GFP fluorescence in upper leaves of PVY-GFP-infected transgenic lines *#2* and *#7* compared to *NN* plants. Photographs were taken at 4 dpi. Scale bars, 5 cm. (B–C) qRT-PCR and

immunoblot analyses confirmed significantly elevated PVY genomic RNA (B) and GFP protein (C) levels in transgenic lines (n = 9 plants from 3 independent experiments). Ponceau S staining verified equal loading. (D) GFP fluorescence in upper leaves of PVY-GFP-infected plants was milder in *NbeIF4A3* silenced plants than in control plants. Leaves of TRV:*NbeIF4A3* and TRV:*mCherry*-treated plants at 8 dpi were then inoculated with PVY-GFP. Representative images of upper leaves under UV light at 4 dpi are shown. Scale bars, 5 cm. (E–F) qRT-PCR and immunoblot showed decreased expression of *NbeIF4A3* and PVY genomic RNA (E) and GFP protein (F) in upper leaves of TRV:*NbeIF4A3*-infected plants compared to TRV:*mCherry* control plants at 12 dpi. (G) Transgenic lines *#2* and *#7* displayed increased GFP fluorescence in upper leaves at 4 dpi. Scale bars, 5 cm. (H–I) TuMV genomic RNA and GFP protein accumulation levels were significantly increased in upper leaves of transgenic lines *#2* and *#7* than *NN* plants, as determined by qRT-PCR and immunoblotting analyses. Actin blots were used as the loading control. (J) GFP fluorescence in upper leaves of TuMV-GFP-infected plants were milder in *NbeIF4A3* silenced plants than in control plants. (K–L) qRT-PCR and immunoblot analyses showed decreased expression of *NbeIF4A3* and TuMV genomic RNA (K) and GFP protein (L) in TRV:*NbeIF4A3*-infected plants compared to TRV:*mCherry* control plants at 12 dpi. Ponceau S-stained Rubisco was used as the loading control. (M) MeRIP-qPCR analysis revealed increased $m^6A$ levels in the *CP* coding region of TuMV in TRV:*NbeIF4A3*-treated plants compared to the control plants. Data are mean ± SE (n = 9 or 3 plants from 3 independent experiments). One-way ANOVA for B, C, H, I; two-tailed Student's *t*-test for E–F and K–M ($*P < 0.05$, $**P < 0.01$, $***P < 0.001$, $****P < 0.0001$).

Supplementary Table S1. Primers and probes used in this study.

| Primers or probes name | sequence (5′ to 3′) | Usage for PCR/qPCR or plasmid construction |
|---|---|---|
| SCMV-CP-qRT-F | GGCGAGACTCAGGAGAATACA | qRT-PCR detection of SCMV RNA |
| SCMV-CP-qRT-R | ACACGCTACACCAGAAGACAC T | |
| PVY CP-RT-F | TCGTAATCTGCGCGATGGAA | qRT-PCR detection of PVY RNA |
| PVY CP-RT-R | TACTGATGCCACCGTCCAAC | |
| ZmUbi-qRT-F | GGAAAAACCATAACCCTGGA | qRT-PCR detection of *Ubiquitin*, as normalization of qRT-PCR data in maize |
| ZmUbi-qRT-R | ATATGGAGAGAGGGCACCAG | |
| Actin-qF | TGCCATTCTCCGTCTTGACT | qRT-PCR detection of *Actin* as internal control in *N. benthamiana* |
| Actin-qR | TGCAGTCTCGAGTTCCTGTT | |
| ZmeIF4A3-qF | TCGTGAGCTTTACATCCATC | qRT-PCR detection of *ZmeIF4A3* in maize |
| ZmeIF4A3-qR | TGTGGTTCGGTTTGGAACGCT CCT | |
| ZmeIF4A3-like-qF | TAGCTCGCTCCAACTCATGT | qRT-PCR detection of *ZmeIF4A3-like* in maize |
| ZmeIF4A3-like-qR | AGGCACAGTAACAGTGTTATT | |
| qF-ZmMTA | CGTCGCATCGCAGAAGACAG | qRT-PCR detection of *ZmMTA* in maize |
| qR-ZmMTA | GTGGAGTTGGTGGAGGTTGGA | |
| qF-ZmECT23 | ATACACCAAGCAACAGAACTC | qRT-PCR detection of *ZmECT23* in maize |
| qR-ZmECT23 | TTCACCTCATCCACACCAAT | |
| qF-NIa-Pro | ggtgtgcttagtcggagtgaat | MeRIP-qRT-PCR detection of SCMV peak5 m$^6$A level in maize |
| qR-NIa-Pro | tcaacgctcatatcaaggtcttct | |
| qF-24857 | CCGCAGTATCAGTCATTGTAA | MeRIP-qRT-PCR detection of *Zm00001d024857* m$^6$A level in WT and *ZmeIF4A3*-KD plants |
| qR-24857 | AACCAGGCATTCAAGTAACG | |
| qF-13599 | ACAGAGCCATTAGCCATACA | MeRIP-qRT-PCR detection of *Zm00001d013599* m$^6$A level in WT and *ZmeIF4A3*-KD plants |
| qR-13599 | GAAGATTGAGGTTGACGACTT | |
| qF-TuMV-m6A | tggctgattacgaactgacg | MeRIP-qRT-PCR detection of TuMV *CP* m$^6$A level in TRV-*mCherry* or TRV-*NbeIF4A3*-inoculated *N. benthamiana* plants |
| qR-TuMV-m6A | atgcctctccgtgttctctacc | |
| NbeIF4A3-qF | TGGAGATATGCCACAGAAGGA GAG | qRT-PCR detection of *NbeIF4A3* in *N. benthamiana* |
| NbeIF4A3-qR | AGAGCGACCAATGCGATGAAT G | |
| TuMV-CP-qRT-F | tggctgattacgaactgacg | qRT-PCR detection of SCMV RNA |
| TuMV-CP-qRT-R | ctgcctaaatgtgggtttgg | |
| qF-Zm00001d002601 | CCGCCTTACTTCGAGATGA | qRT-PCR detection the expression of *Zm00001d002601* in WT and *ZmeIF4A3*-KD plants |
| qR-Zm00001d002601 | CTTCCGTGCCTTCTTGGA | |
| qF-Zm00001d013066 | GCTTTGCTATTGCCAAGTTCTT | qRT-PCR detection the expression of *Zm00001d013066* in WT and *ZmeIF4A3*-KD plants |
| qR-Zm00001d013066 | GCTTGTACGAGTTCTTCACCTT | |
| qF-Zm00001d013481 | AGCATGGAAGAGGAGGTCAAT | qRT-PCR detection the expression of *Zm00001d013481* in WT and *ZmeIF4A3*-KD plants |
| qR-Zm00001d013481 | AGCAGAACCAGCCAATATAAG TC | |
| qF-Zm00001d033961 | TTGCTGGTGTTGAGGTTATGC | qRT-PCR detection the expression of *Zm00001d033961* in WT and *ZmeIF4A3*-KD plants |
| qR-Zm00001d033961 | CCACTCTGCCTTCACAATGTC | |
| qF-Zm00001d046210 | ACTTCTCGCTTGGATGGTTCA | qRT-PCR detection the expression of *Zm00001d046210* in WT and *ZmeIF4A3*-KD plants |
| qR-Zm00001d046210 | ATGTCGCCGTAGTAGTCTCTG | |
| qF-Zm00001d029087 | CTGAGGATGCTGCTGGTGAA | qRT-PCR detection the expression of *Zm00001d029087* in WT and *ZmeIF4A3*-KD plants |
| qR-Zm00001d029087 | CTTGGTATGTGCTGGTGATGAT AA | |

| | | |
|---|---|---|
| qF-Zm00001d025140 | CTGACGGTGAACCACAAGATT | qRT-PCR detection the expression of *Zm00001d025140* in WT and *ZmeIF4A3*-KD plants |
| qR-Zm00001d025140 | TCTGACGAAGTTGAGGACGAT | |
| qF-Zm00001d032090 | CGTGCGATTGTGGAGATTGT | qRT-PCR detection the expression of *Zm00001d032090* in WT and *ZmeIF4A3*-KD plants |
| qR-Zm00001d032090 | GCTCTTGGAGTCTTGTTGAATTG | |
| F-222668229 | TTCTTACTACCATCCGTCCTTCCT | PCR detection of the mutant site in *ZmeIF4A3*-EMS plant |
| R-222668229 | CCGCAGTGTCCTCCTCTTGAT | |
| F-222667549 | AGAGACTGATGATGATGAGAC | PCR detection of the mutator insertion in *ZmeIF4A3*-KD mutant |
| R-222667549 | AGGAAGGACGGATGGTAG | |
| Mu67 | GAAGCCAACGCCAWCGCCTCYATTTCGTCGAAT | |
| selectqF | ATGCAGCGACTCAGCCTCTG | qRT-PCR for SELECT |
| selectqR | TAGCCAGTACCGTAGTGCGTG | |
| select1941down | 5phos/CTTTTGCGGATTCCTCATTGACcagaggctgagtcgctgcat | SELECT assays for $m^6A$ level at position 1941 of the SCMV genomic RNA |
| select1941up | tagccagtaccgtagtgcgtgGTCTCTAAGGAACTTCGTGTAG | |
| control1941down | 5phos/TCGTGTAGTCTTTTGCGGATTCcagaggctgagtcgctgcat | |
| control1941up | tagccagtaccgtagtgcgtgCACCAACTCGTCTCTAAGGAAC | |
| select1996down | 5phos/TTAGGCCACTTGCCGAGCCGTTcagaggctgagtcgctgcat | SELECT assays for $m^6A$ level at position 1996 of the SCMV genomic RNA |
| select1996up | tagccagtaccgtagtgcgtgATGCTGTTGCTACATCTTTCAG | |
| control1996down | 5phos/TGCCGAGCCGTTCCACCAACcagaggctgagtcgctgcat | |
| control1996up | tagccagtaccgtagtgcgtgCATCTTTCAGTTTAGGCCAC | |
| select2966down | 5phos/CCTCACTGCGCTAACCCTCGCTcagaggctgagtcgctgcat | SELECT assays for $m^6A$ level at position 2966 of the SCMV genomic RNA |
| select2966up | tagccagtaccgtagtgcgtgTCGGGTATGAACCAGTATACAG | |
| control2966down | 5phos/GAACCAGTATACAGTCCTCAcagaggctgagtcgctgcat | |
| control2966up | tagccagtaccgtagtgcgtgTAGTCTAAATATATCGGGTA | |
| select3543down | 5phos/CTTGATTTGTATCAAAATCTATcagaggctgagtcgctgcat | SELECT assays for $m^6A$ level at position 3543 of the SCMV genomic RNA |
| select3543up | tagccagtaccgtagtgcgtgACTAAATGTATCCGCTGGCAAG | |
| control3543down | 5phos/ATCAAAATCTATAGTCAGGTcagaggctgagtcgctgcat | |
| control3543up | tagccagtaccgtagtgcgtgCGCTGGCAAGTCTTGATTTG | |
| select3617down | 5phos/TCTGTTGTTGTTAATCTGATTAcagaggctgagtcgctgcat | SELECT assays for $m^6A$ level at position 3617 of the SCMV genomic RNA |
| select3617up | tagccagtaccgtagtgcgtgCCAAGTCGGTAGTGTGGCACTG | |
| control3617down | 5phos/GTGGCACTGTTCTGTTGTTGcagaggctgagtcgctgcat | |
| control3617up | tagccagtaccgtagtgcgtgATTTTCCCCCAAGTCGGTAG | |
| select5413down | 5phos/CCAATACAAGCAGCCGTAGCGTcagaggctgagtcgctgcat | SELECT assays for $m^6A$ level at position 5413 of the SCMV genomic RNA |
| select5413up | tagccagtaccgtagtgcgtgAAGCGTTCCAGCGCCCTTGCAG | |
| control5413down | 5phos/GCCCTTGCAGTCCAATACAAcagaggctgagtcgctgcat | |
| control5413up | tagccagtaccgtagtgcgtgGAATAAGCGAAGCGTTCCAG | |

| | | |
|---|---|---|
| select5651down | 5phos/TCCGAAATTCTCACCAAGAACTcagaggctgagtcgctgcat | SELECT assays for m$^6$A level at position 5651 of the SCMV genomic RNA |
| select5651up | tagccagtaccgtagtgcgtgTTTCCTTTCTTAGTGTATGCAG | |
| control5651down | 5phos/AGTGTATGCAGTTCCGAAATcagaggctgagtcgctgcat | |
| control5651up | tagccagtaccgtagtgcgtgCCCCTTTCCTTTTCCTTTCT | |
| select6465down | 5phos/TCTGCTGAAAATTCACTCCGACTAcagaggctgagtcgctgcat | SELECT assays for m$^6$A level at position 6465 of the SCMV genomic RNA |
| select6465up | tagccagtaccgtagtgcgtgTCCGATACGATACATGTGCTATAG | |
| control6465down | 5phos/CCGATACGATACATGTGCTAcagaggctgagtcgctgcat | |
| control6465up | tagccagtaccgtagtgcgtgTTGGTGCTGTGACGCTACTC | |
| control6556down | 5phos/CTTGACATCTACTAATGGGAcagaggctgagtcgctgcat | SELECT assays for m$^6$A level at position 6556 of the SCMV genomic RNA |
| control6556up | tagccagtaccgtagtgcgtgTATTCCAACTATGTGCTTAC | |
| select6556up | tagccagtaccgtagtgcgtgCATCTACTAATGGGAGACCGCATTG | |
| select6556down | 5phos/CCGTCTATTGTGGATATCCAGTGTcagaggctgagtcgctgcat | |
| select8474down | 5phos/TCCACTACCTGTTGCTGGTGGCcagaggctgagtcgctgcat | SELECT assays for m$^6$A level at position 8474 or 8501 of the SCMV genomic RNA |
| select8474up | tagccagtaccgtagtgcgtgGTAGATGGTGTTCTTGTTCCTG | |
| control8474/8501down | 5phos/CTACCTGTTGCTGGTGGCGTcagaggctgagtcgctgcat | |
| control8474/8501up | tagccagtaccgtagtgcgtgGGTGTTCTTGTTCCTGTTCC | |
| select8501down | 5phos/TCCAGTAGATGGTGTTCTTGTTcagaggctgagtcgctgcat | |
| select8501up | tagccagtaccgtagtgcgtgGGTGTGTTGCCTTGTGCTGGAG | |
| Biotin SCMV Probe | 5′-Biotin-CACAACUGGAGGACAAUGCGGUCUC | RNA Probe for dot blot and EMSA |
| F-AD-ZmUPF3 | GCCATGGAGGCCAGTGAATTCATGAAGGACCCGGCGCACCG | AD-ZmUPF3 |
| R-AD-ZmUPF3 | cagctcgagctcgatggatcccCGAGCCTGAACTTGACTTCT | |
| F-AD-ZmCCR4 | GCCATGGAGGCCAGTGAATTCatgctgagcgtggttcgggt | AD-ZmCCR4 |
| R-AD-ZmCCR4 | cagctcgagctcgatggatcccacgtctgattcttggcttgc | |
| F-AD-ZmUPF2 | GCCATGGAGGCCAGTGAATTCATGGACAATGCTCAGAGTGA | AD-ZmUPF2 |
| R-AD-ZmUPF2 | cagctcgagctcgatggatcccTCTTCTCCTGCCATAACTGT | |
| F-AD-ZmeIF4A3 | CCATGGAGGCCAGTGAATTCATGGCGGCGCCCACCACCTC | AD-ZmeIF4A3 |
| R-AD-ZmeIF4A3 | gctcgagctcgatggatcccAATAAGATCAGCAACATTCATT | |
| F-BD-NIa-Pro | GAGGCCGAATTCatgTCGAAATCGATGATGGCAGG | BD-NIa-Pro |
| R-BD-NIa-Pro | ATGCGGCCGCTGCAGTTGTTCTTCAACGCTCATATCAA | |
| F-BD-ZmECT23 | GGCCATGGAGGCCGAATTCatgATGGCGGCTGTAGGCAGCTT | BD-ZmECT23 |
| R-BD-ZmECT23 | GCTAGTTATGCGGCCGCTGCAGACAGCCATTTAGACCCGCCA | |
| F-BD-NIa-Pro1-194 | GCCATGGAGGCCGAATTCatgTCGAAATCGATGATGGCAGG | BD-NIa-Pro1-194 |

| | | |
|---|---|---|
| R-BD-NIa-Pro1-194 | GCTAGTTATGCGGCCGCTGCAGCACAAGATTATTGATGTAGTC | |
| F-BD-NIa-Pro195-242 | TGGCCATGGAGGCCGAATTCatgCAAACCAACAAGTGGGAAA | BD-NIa-Pro195-242 |
| R-BD-NIa-Pro195-242 | ATGCGGCCGCTGCAGTTGTTCTTCAACGCTCATATCAA | |
| F-pCold-3Flag-CCR4 | gaaggtaggcatatggagctcggtaccATGGACTACAAAGACCATGA | pCold-TF-3Flag-CCR4 |
| R-pCold-3Flag-CCR4 | gattacctatctagactgcagacgtctgattcttggcttgca | |
| F-pCold-GFP-ECT23 | aggcatatggagctcggtaccATGGTGAGCAAGGGCGAGGAGCT | pCold-TF-GFP-ECT23 |
| R-pCold-GFP-ECT23 | gattacctatctagactgcagACAGCCATTTAGACCCGCCAA | |
| F-pCold-GFP | gcatatggagctcggtaccATGGTGAGCAAGGGCGAGGAGCT | pCold-TF-GFP |
| R-pCold-GFP | cagagattacctatctagactgcagCTTGTACAGCTCGTCCATGC | |
| F-pCold-TF-ZmeIF4A3-3myc | gaaggtaggcatatggagctcggtaccATGGCGGCGCCCACCACCTC | pCold-ZmeIF4A3-3myc |
| R-pCold-TF-ZmeIF4A3-3myc | agattacctatctagactgcagTCCGGTGGATCCAAGATCCTCC | |
| F-pCold-TF-3Flag-ZmMTA | gaaggtaggcatatggagctcggtaccATGGACTACAAAGACCATGA | pCold-3Flag-ZmMTA |
| R-pCold-TF-3Flag-ZmMTA | attacctatctagactgcagGGCGGGCCTCTCGCCACCATCT | |
| F-ZmMTA-DsRed | CACCACCTGTTCCTGGGGCCCATGGAAGCGCAGACCGACTC | pGD-ZmMTA-DsRed |
| R-ZmMTA-DsRed | TTATCTAGATCCGGTGGATCCCGGCGGGCCTCTCGCCACCATC | |
| F-pGEX-NIa-Pro | ATGAAAACCTGTATTTTCAGGGATCCATGTCGAAATCGATGATGGCAGG | pGEX-NIa-Pro |
| R-pGEX-NIa-Pro | GCCGCTTAGTGGTGGTGGTGGTGGTGCTCGAGTTGTTCTTCAACGCTCATATC | |
| F-NLuc-ZmeIF4A3 | GAGCTCGGTACCCGGGATCCATGGCGGCGCCCACCACCTC | NLuc-ZmeIF4A3 and NLuc-ZmeIF4A3-like |
| R-NLuc-ZmeIF4A3 | GCGTACGAGATCTGGTCGACAATAAGATCAGCAACATTCATT | |
| F-Cluc-NIa-Pro | ACGCGTCCCGGGGCGGTACCTCGAAATCGATGATGGCAGG | Cluc-NIa-Pro |
| R-Cluc-NIa-Pro | ACGAAAGCTCTGCAGGTCGATTGTTCTTCAACGCTCATATCA | |
| F-NLuc-ZmeIF4A3 | GAGCTCGGTACCCGGGATCCATGGCGGCGCCCACCACCTC | NLuc-ZmeIF4A3del287-316 |
| R-ZmeIF4A3-delect287-368 | CGCCACACCCTTGCGCACCTTTCTCTTAGTATTGC | |
| F-ZmeIF4A3-delect287-368 | ACTAAGAGAAAGGTGCGCAAGGGTGTGGCGATCAA | |
| R-NLuc-ZmeIF4A3 | GCGTACGAGATCTGGTCGACAATAAGATCAGCAACATTCATT | |
| F-NLuc-ZmeIF4A3 | GAGCTCGGTACCCGGGATCCATGGCGGCGCCCACCACCTC | NLuc-ZmeIF4A3del53-250 |
| R-ZmeIF4A3-delect53-250 | CTCAACAGCAACAAAGTAGCCGTAGATGCCGCGGA | |
| F-ZmeIF4A3-delect53-250 | GGCATCTACGGCTACTTTGTTGCTGTTGAGAAAGA | |
| R-NLuc-ZmeIF4A3 | GCGTACGAGATCTGGTCGACAATAAGATCAGCAACATTCATT | |
| F-Nluc-ZmeIF4A3-287-342 | GAGCTCGGTACCCGGGATCCGATTGGCTCACTGAAAAAAT | Nluc-ZmeIF4A3-287-342 |

| | | |
|---|---|---|
| R-Nluc-ZmeIF4A3-287-342 | GCGTACGAGATCTGGTCGACA ACCTGCTGAACGTCCAGTC | |
| F-Nluc-ZmeIF4A3-343-368 | GAGCTCGGTACCCGGGATCCT CACTTGTCATAAATTATGA | Nluc-ZmeIF4A3-343-368 |
| R-Nluc-ZmeIF4A3-343-368 | GCGTACGAGATCTGGTCGACC CCAAAACGACCAGAACGAC | |
| F-Nluc-ZmeIF4A3-287-296 | GAGCTCGGTACCCGGGATCCG ATTGGCTCACTGAAAAAAT | Nluc-ZmeIF4A3-287-296 |
| R-Nluc-ZmeIF4A3-287-296 | CGCGTACGAGATCTGGTCGAC ATTTGTACGCATTTT | |
| F-Nluc-ZmeIF4A3-297-306 | GAGCTCGGTACCCGGGATCCA ACTTCACCGTATCA | Nluc-ZmeIF4A3-297-306 |
| R-Nluc-ZmeIF4A3-297-306 | CGCGTACGAGATCTGGTCGAC GTCACCGTGCATAGC | |
| F-Nluc-ZmeIF4A3-307-316 | GAGCTCGGTACCCGGGATCCA TGCCCCAACAAGAA | Nluc-ZmeIF4A3-307-316 |
| R-Nluc-ZmeIF4A3-307-316 | CGCGTACGAGATCTGGTCGAC CATAATGGCATCCCT | |
| F-Nluc-ZmeIF4A3-317-326 | GAGCTCGGTACCCGGGATCCG GTGAGTTCAGGTCT | Nluc-ZmeIF4A3-317-326 |
| R-Nluc-ZmeIF4A3-317-326 | CGCGTACGAGATCTGGTCGAC CACACGAGTTGCGCC | |
| F-Nluc-ZmeIF4A3-327-342 | GAGCTCGGTACCCGGGATCCC TAATAACTACAGATGTTT | Nluc-ZmeIF4A3-327-342 |
| R-Nluc-ZmeIF4A3-327-342 | GCGTACGAGATCTGGTCGACA ACCTGCTGAACGTCCAGTC | |
| F-NLuc-ZmeIF4A3 | GAGCTCGGTACCCGGGATCCA TGGCGGCGCCCACCACCTC | Nluc-ZmeIF4A3-del287-316 |
| R-nluc-ZmeIF4A3-del287-316 | GCGCCAGACCTGAACTCACCC ACCTTTCTCTTAGT | |
| F-nluc-ZmeIF4A3-del287-316 | GCAATACTAAGAGAAAGGTGG GTGAGTTCAGGTCT | |
| R-NLuc-ZmeIF4A3 | GCGTACGAGATCTGGTCGACA ATAAGATCAGCAACATTCATT | |
| F-Cluc-NIa-Pro1-157 | ACGCGTCCCGGGGCGGTACCT CGAAATCGATGATGGCAGG | Cluc-NIa-Pro1-157 |
| R-Cluc-NIa-Pro1-157 | ATACGAACGAAAGCTCTGCAG GTCGAATCTACTAATGGGAGA CCG | |
| F-Cluc-NIa-Pro158-242 | CTCGTACGCGTCCCGGGGCGG TACCGTCAAGAGTAAGCACAT AGT | Cluc-NIa-Pro158-242 |
| R-Cluc-NIa-Pro158-242 | ACGAACGAAAGCTCTGCAGGT CGATTGTTCTTCAACGCTCATA T | |
| F-Cluc-NIa-Pro1-194 | CTCGTACGCGTCCCGGGGCGG TACCTCGAAATCGATGATGGC AGG | Cluc-NIa-Pro1-194 |
| R-Cluc-NIa-Pro1-194 | TACGAACGAAAGCTCTGCAGG TCGACACAAGATTATTGATGTA GT | |
| F-Cluc-NIa-Pro195-242 | TCGTACGCGTCCCGGGGCGGT ACCCAAACCAACAAGTGGGA AAAAG | Cluc-NIa-Pro195-242 |
| R-Cluc-NIa-Pro195-242 | TACGAACGAAAGCTCTGCAGG TCGATTGTTCTTCAACGCTCAT AT | |
| F-nLUC-ZmECT23 | GAGCTCGGTACCCGGGATCCA TGGCGGCTGTAGGCAGCTT | NLUC-ZmECT23 |
| R-nLUC-ZmECT23 | CGCGTACGAGATCTGGTCGAC ACAGCCATTTAGACCCGCCA | |
| F-Cluc-ZmCCR4 | TACGCGTCCCGGGGCGGTACC atgctgagcgtggttcgggt | Cluc-ZmCCR4 |

| | | |
|---|---|---|
| R-Cluc-ZmCCR4 | GAACGAAAGCTCTGCAGGTCG Aacgtctgattcttggcttgc | |
| F-ZmeIF4A3-3Flag | CTCTACAAGATCTCGAGCTCA AGCTTcgATGGCGGCGCCCACC ACCTC | pGD-ZmeIF4A3-3Flag and pGD-ZmeIF4A3-like-3Flag |
| R-ZmeIF4A3-3Flag | CGTCTTTGTAGTCCATGTCGAC TGCAGaAATAAGATCAGCAAC ATTC | |
| F-pGD-ZmeIF4A3-3Flag | AAGATCTCGAGCTCAAGCTTcg ATGGCGGCGCCCACCACCTC | pGD-ZmeIF4A3del53-250-3Flag |
| R-ZmeIF4A3delect53-250 | CTCAACAGCAACAAAGTAGCC GTAGATGCCGCGGA | |
| F-ZmeIF4A3delect53-250 | GGCATCTACGGCTACTTTGTTG CTGTTGAGAAAGA | |
| R-pGD-ZmeIF4A3-3Flag | CGTCTTTGTAGTCCATGTCGAC TGCAGaAATAAGATCAGCAAC ATTCATT | |
| F-pGD-ZmeIF4A3-3Flag | AAGATCTCGAGCTCAAGCTTcg ATGGCGGCGCCCACCACCTC | pGD-ZmeIF4A3del287-318-3Flag |
| R-nluc-ZmeIF4A3-del287-316 | GCGCCAGACCTGAACTCACCC ACCTTTCTCTTAGT | |
| F-nluc-ZmeIF4A3-del287-316 | GCAATACTAAGAGAAAGGTGG GTGAGTTCAGGTCT | |
| R-pGD-ZmeIF4A3-3Flag | CGTCTTTGTAGTCCATGTCGAC TGCAGaAATAAGATCAGCAAC ATTCATT | |
| F-pGD-ZmeIF4A3-3Flag | AAGATCTCGAGCTCAAGCTTcg ATGGCGGCGCCCACCACCTC | pGD-ZmeIF4A3del287-368-3Flag |
| F-ZmeIF4A3delect287-368 | ACTAAGAGAAAGGTGCGCAA GGGTGTGGCGATCAA | |
| R-ZmeIF4A3delect287-368 | CGCCACACCCTTGCGCACCTT TCTCTTAGTATTGC | |
| R-pGD-ZmeIF4A3-3Flag | CGTCTTTGTAGTCCATGTCGAC TGCAGaAATAAGATCAGCAAC ATTCATT | |
| F-in-EGFP-NIa-Pro | GCTGTACAAGtCTGCAGTCGA CTCGAAATCGATGATGGCAGG | pGD-EGFP-NIa-Pro |
| R-in-EGFP-NIa-Pro | TATCTAGATCCGGTGGATCCTT GTTCTTCAACGCTCATATCA | |
| F-EGFP-ZmeIF4A3 | CTGTACAAGtCTGCAGTCGAC ATGGCGGCGCCCACCACCTC | pGD-EGFP-ZmeIF4A3 and pGD-EGFP-ZmeIF4A3-like |
| R-EGFP-ZmeIF4A3 | TTATCTAGATCCGGTGGATCCA ATAAGATCAGCAACATTCATT | |
| F-EGFP-ZmeIF4A3 | CTGTACAAGtCTGCAGTCGAC ATGGCGGCGCCCACCACCTC | pGD-ZmeIF4A3del287-316-EGFP |
| R-nluc-ZmeIF4A3-del287-316 | GCGCCAGACCTGAACTCACCC ACCTTTCTCTTAGT | |
| F-nluc-ZmeIF4A3-del287-316 | GCAATACTAAGAGAAAGGTGG GTGAGTTCAGGTCT | |
| R-EGFP-ZmeIF4A3 | TTATCTAGATCCGGTGGATCCA ATAAGATCAGCAACATTCATT | |
| F-GUS-3Flag | CAAGATCTCGAGCTCAAGCTTc gATGTTACGTCCTGTAGAAAC | pGD-GUS-3Flag |
| R-GUS-3Flag | CGTCTTTGTAGTCCATGTCGAC TGCAGaTTGTTTGCCTCCCTGC TGCG | |
| F-in-*ZmECT23*-vigs | AAATGTCCGAGTCTGAGgtaccT ATGCTCCATATGGTGTTGC | pCMV-N81-VIGS-*ZmECT23* |

| | | |
|---|---|---|
| R-in-*ZmECT23*-vigs | ATTAGGAAGGGGAGGTTCTAG aTTTTTCAAACCTTTCACCTC | |
| F-vigs-*ZmMTA* | AAATGTCCGAGTCTGAGgtaccT TACAAGCGTGTTGAAGAAA | pCMV-N81-VIGS-*ZmMTA* |
| R-vigs-*ZmMTA* | TTAGGAAGGGGAGGTTCTAGa TCAGGTTTTCTGCTTGTTTC | |
| F-vigs-*ZmeIF4A3/like* | AAATGTCCGAGTCTGAGgtaccC ACTGCGGACCAGAGCCATC | pCMV-N81-VIGS-*ZmeIF4A3/ZmeIF4A3-like* |
| R-vigs-*ZmeIF4A3/like* | TTAGGAAGGGGAGGTTCTAGa CACAAGGATCCTAACCGGTTC | |
| F-vigs-*ZmeIF4A3-like* | AAATGTCCGAGTCTGAGgtaccgt ttactttttggtgacggct | pCMV-N81-VIGS-*ZmeIF4A3-like* |
| R-vigs-*ZmeIF4A3-like* | TTAGGAAGGGGAGGTTCTAGa GTTATATTCTTAGTGGTTAA | |
| F-NIa-Pro-3Flag | GAAGCTTCGAATTCTGCAGTC GACGTCGAAATCGATGATGGC AGG | pGD-NIa-Pro-3Flag |
| R-NIa-Pro-3Flag | TATCTAGATCCGGTGGATCCTT GTTCTTCAACGCTCATATCA | |
| F-NIa-Pro1-194-3Flag | GCTTCGAATTCTGCAGTCGAC GATGTCGAAATCGATGATGGC AGG | pGD-NIa-Pro1-194-3Flag |
| R-NIa-Pro1-194-3Flag | TTATCTAGATCCGGTGGATCCC ACAAGATTATTGATGTAGTC | |
| F-SCMV-GUS-3Flag | CAATCCGGACCTGGGCCCATG TTACGTCCTGTAGAAAC | pSCMV-GUS-3Flag |
| R-SCMV-GUS-3Flag | GGATTGATGGAAAACATCTTC CCCGGGTTTGTCGTCG | |
| F-SCMV-ZmeIF4A3-3Flag | CATCAATCCGGACCTGGGCCC ATGGCGGCGCCCACCACCTCC | pSCMV-ZmeIF4A3-3Flag |
| R-SCMV-ZmeIF4A3-3Flag | GGATTGATGGAAAACATCTTC CCCGGGTTTGTCGTCG | |
| F-pSCMV-$G^{6556}$-GFP | AAAACCATCTAGAGAGTCAAG GGTGTGCTTAGTCGG | pSCMV-$G^{6556}$-GFP |
| F-over-NIaA-G | ATCCACAATAGACGGgCAATG CGGTCTCCCA | |
| R-over-NIaA-G | TAATGGGAGACCGCATTGcCC GTCTATTGTG | |
| R-pSCMV-$G^{6556}$-GFP | AAGTTGAGACCTAGGTTTCCA AAGTGATTTTGAAAT | |
| F-SCMV-NIa | AAGTTTTCAAAACCATCTAGA GAGTCAAGGGTGTGCTTAGT | pSCMV-$G^{6556}$-GFP/ΔGDD and pSCMV-GFP/ΔGDD |
| F-delGDD | CGAATGTTCGCCAATTTGCTAC TTGCAGTG | |
| R-delGDD | CACTGCAAGTAGCAAATTGGC GAACATTCG | |
| R-SCMV-NIb | AACTCAAAGTTGAGACCTAGG TTTCCAAAGTGATTTTGAAA | |
| F-TRV-NbeIF4A3 | AAGGTTACCGAATTCTCTAGA GCAGCTCAAACAGAAAAGGT G | pTRV2:NbeIF4A3 |
| R-TRV-NbeIF4A3 | GAGACGCGTGAGCTCGGTACC ATCAATAATTTTATACCCCT | |
| F-pGD-3Flag-ZmMTA | GCTTCGAATTCTGCAGTCGAC GATGGAAGCGCAGACCGACTC | pGD-3Flag-ZmMTA |
| R-pGD-3Flag-ZmMTA | TTATCTAGATCCGGTGGATCCG GCGGGCCTCTCGCCACCATC | |
| F-pGD-ZmeIF4A3-3Myc | CTCTACAAGATCTCGAGCTCA AGCTTCGATGGCGGCGCCCAC CACCTC | pGD-ZmeIF4A3-3Myc |
| R-pGD-ZmeIF4A3-3Myc | TGCTCCATGTCGACTGCAGAA ATAAGATCAGCAACATTCATT | |
| F-his-ZmeIF4A3-like-3Flag | ACGACGACGACAAGGCCATG | Pet30a-His-ZmeIF4A3-like-3Flag |

| | | |
|---|---|---|
| | GCTATGGCGGCGCCCACCACC TC | |
| R-his-ZmeIF4A3-like-3Flag | AGTGCGGCCGCAAGCTTGTCG ACTTTGTCGT | |
| F-BD-ZmeIF4A3-like | ATGGCCATGGAGGCCGAATTC ATGGCGGCGCCCACCACCTCC | BD-ZmeIF4A3-like |
| R-BD-ZmeIF4A3-like | CTAGTTATGCGGCCGCTGCAG AATAAGATCAGCAACATTCAT | |

Supplementary Table S2. The m$^6$A modification sites of the SCMV genome RNA were predicted using SRAMP and marked in red text with an underline for emphasis.

| Position | Sequence context | Annotation | Score | Decision |
|---|---|---|---|---|
| 1941 | AAUCCGCAAAAGACUACACGAAGUU | CDS | 0.657 | High confidence |
| 1996 | CAAGUGGCCUAAACUGAAAGAUGUA | CDS | 0.577 | Moderate confidence |
| 2966 | AGCGCAGUGAGGACUGUAUAUUGGU | CDS | 0.607 | High confidence |
| 3543 | AUACAAAUCAAGACUUGCCAGCGGA | CDS | 0.608 | High confidence |
| 3617 | AACAACAACAGAACAGUGCCACACU | CDS | 0.602 | High confidence |
| 5413 | UGCUUGUAUUGGACUGCAAGGGCGC | CDS | 0.684 | Very High confidence |
| 5651 | GAGAAUUUCGGAACUGCAUACACUA | CDS | 0.675 | Very High confidence |
| 6465 | AUUUUCAGCAGAACUAUAGCACAUG | CDS | 0.616 | High confidence |
| 6556 | CACAACUGGAGGACAAUGCGGUCUC | CDS | 0.665 | High confidence |
| 8474 | ACAGGUAGUGGAACAGGAACAAGAA | CDS | 0.564 | Moderate confidence |
| 8501 | CCAUCCACUGGAACUCCAGCACAAG | CDS | 0.608 | High confidence |
